# Examining a Dutch short form of The Balanced Inventory of Desirable Responding Version 6: comparing polytomous and dichotomous scoring methods in a multidimensional framework

**DOI:** 10.3389/fpsyg.2025.1532969

**Published:** 2025-06-13

**Authors:** Mirthe G. C. Noteborn, Martin Hildebrand, Jelle J. Sijtsema, Stefan Bogaerts, Jaap J. A. Denissen

**Affiliations:** ^1^Department of Developmental Psychology, Tilburg University, Tilburg, Netherlands; ^2^Private Practice, De Bilt, Netherlands; ^3^Lelystad Prison, Lelystad, Netherlands; ^4^Department of Educational Sciences/GION, University of Groningen, Groningen, Netherlands; ^5^Fivoor Science and Treatment Innovation, Rotterdam, Netherlands; ^6^Department of Developmental Psychology, Utrecht University, Utrecht, Netherlands

**Keywords:** socially desirable responding, BIDR, impression management, self-deceptive enhancement, multidimensional item response theory

## Abstract

**Introduction:**

The 40-item Balanced Inventory of Desirable Responding Version 6 (BIDR) is a widely used tool to measure two components of social desirability: Self-Deceptive Enhancement (SDE) and Impression Management (IM). In three studies, we aimed to create and validate a short form of the Dutch language version of the 40-item BIDR.

**Methods:**

In Study 1 (general population sample *N* = 577), item properties were examined using (Multidimensional) Item Response Theory (IRT) for both dichotomous and polytomous scoring methods to create a short form. In Study 2 (general population sample *N* = 719), IRT analyses of Study 1 were replicated, and the nomological network of the short form was examined by investigating its relation with the Big Five personality traits and deviant traits and thoughts. Study 3 (men from the general population *N* = 100) investigated whether SDE and IM could detect response bias in self-reported aggression. All samples consisted of individuals volunteering to participate in scientific research (recruited in various ways) in a low-stake condition.

**Results:**

This yielded a short form containing 10 SDE and 10 IM dichotomously scored items (BIDR-D20). While results indicated a loss of information compared to the original version, the overall psychometric qualities were equal to or sometimes better compared to the BIDR (Version 6). Across studies, dichotomous scoring was generally better than polytomous scoring in terms of model fit, estimated IRT parameters, and internal consistency. Both forms correlated with self-reported aggression, but SDE and IM failed to detect response bias in the current sample.

**Conclusion:**

The BIDR-D20 could be a worthy replacement for the 40-item BIDR (Version 6), with the same properties and less time-consuming. However, more research is needed to establish the short measure’s predictive validity as a response bias.

## Introduction

The 40-item Balanced Inventory of Desirable Responding Version 6 (BIDR-6) (Paulhus, 1994) is one of the most widely used self-report questionnaires to measure socially desirable responding (SDR). Although the BIDR has several good psychometric properties (i.e., satisfactory reliability, convergent, and discriminant validity), the instrument has been criticized for its equivocal factor structure and lack of unidimensionality. Also, the model fit has been found to be suboptimal, leading researchers to remove items to increase fit (Lanyon and Carle, 2007; Li and Li, 2008; Li et al., 2015). In addition, scholars have differing views on which scoring method of the BIDR (i.e., dichotomous or polytomous) is preferable. Some researchers favor dichotomous scoring because it singles out abnormal responses (Paulhus, 1994), whereas others found that polytomous scoring is more reliable (higher alpha, test–retest reliability) and valid (convergent validity; Cervellione et al., 2009; Vispoel and Kim, 2014; Vispoel and Tao, 2013). Finally, although this may seem as an irrelevant point for a questionnaire that takes up to 5–10 min to complete, filling out the relatively long BIDR-6 may limit its utility when used in conjunction with other self-reports, which is often the case in clinical (forensic) assessments. Longer questionnaires are more time-consuming and “may increase transient measurement errors, as respondents may become frustrated or respond carelessly due to boredom or fatigue” (Hart et al., 2015, p. 2; also Schmidt et al., 2003; Stanton et al., 2002). This may be especially apparent in clinical forensic settings where concentration, cooperation, and response rates are often low (Moser et al., 2004; Young and Cocallis, 2019). Hence, several short forms have been developed, in different languages (e.g., Asgeirsdottir et al., 2016; Hart et al., 2015; Subotić et al., 2016). In line with the advocacy to consider cultural impact on SDR questionnaires, Lalwani et al. (2006) demonstrated in their study that national culture and ethnicity predicts distinct patterns of SDR. Thereby, one could argue that the item selection of the short forms is (partly) driven by these distinct patterns of SDR. The little overlap between the included items in these short forms could be evidence of this cultural impact.

Therefore, the purpose of the current study is to create and validate a Dutch short form of the original 40-item BIDR (Version 6), which currently does not exist. Using Item Response Theory (IRT), we focused on the multidimensionality of the BIDR as well as the appropriateness of scoring methods. Moreover, we investigated whether this short form outperforms the 40-item BIDR-6 in terms of psychometric properties, nomological network, and validity.

### Social desirable responding

SDR can be described as the inclination to provide biased, distorted, or excessively positive self-descriptions to present oneself in a way that creates a favorable impression on others (Paulhus, 2002; see also Furnham, 1986; Nederhof, 1985). SDR has long been recognized as a potential confounder when using self-report measures, especially in a context with a high possibility of secondary gains or losses, where there are potential gains or losses, such as in personnel selection or forensic and clinical settings, where individuals may have a strong motivation to portray themselves in a positive light (Hanson and Bussière, 1998; Hildebrand et al., 2018; Tan and Grace, 2008; Tatman et al., 2009). Banse et al. (2010), for example, discussed the problems associated with self-report measures of sexual (e.g., pedophilic) interest and concluded that “their validity is jeopardized by impression management and deliberate faking. The general problem of transparency in direct measures is all the more critical if disclosure of personal information is highly embarrassing, socially undesirable, or has legal implications, as it is commonly the case in forensic contexts” (p. 320).

The most common strategy for addressing socially desirable response bias is to use measures designed to assess individuals’ SDR tendencies. SDR scales typically contain descriptions of desirable behaviors or traits (e.g., “I never hesitate to go out of my way to help someone in trouble”; Crowne and Marlowe, 1960). Scores on these instruments are sometimes used to flag possible invalid responses that may be discarded or to control for a desirability response bias statistically. In addition, SDR measures are utilized to assess convergent and/or divergent validity; demonstrating that scores from the SDR scale align with other measures as expected and using them as dependent variables in controlled experiments to emphasize situations most likely to prompt (Hildebrand et al., 2018; Vispoel and Kim, 2014; Vispoel and Tao, 2013; see also Tan and Grace, 2008). Although several ways to handle SDR have been proposed, discussion on the use of SDR measures is still ongoing. Whereas exclusion of flagged responses and statistically controlling for desirable responses are encouraged by some researchers (Martínez-Catena et al., 2017; van de Mortel, 2008), others stress that this might remove valid variance in personality differences (McCrae and Costa, 1983; Pauls and Stemmler, 2003; Uziel, 2010).

Several instruments have been created and validated to uncover SDR. Most of these measures operationalized SDR as a unidimensional construct (e.g., Edwards, 1957; Crowne and Marlowe, 1960; Crowne and Marlowe, 1964; Eysenck and Eysenck, 1964; Hofstee, 2003). Conversely, due to low correlations between different SDR measures and the results of factor analyses, some researchers have questioned the one-dimensional approach to understanding SDR (Edwards et al., 1962; Messick, 1962; Wiggins, 1964). Recognizing the empirical divergence of SDR, Paulhus (1984, 1991) proposed that measures of social desirability evaluate two distinct components, which he called Impression Management (IM) and Self-Deceptive Enhancement (SDE). IM involves intentionally distorting responses to create a positive impression on others (Paulhus, 1984; Stöber et al., 2002) and is sometimes called lying or faking. Alternatively, SDE involves portraying oneself in a positive light to maintain a positive self-image. SDE is associated with self-deceptive overconfidence and is closely linked to narcissism. SDE is a sincerely believed self-deception (Paulhus, 1984) and is deeply ingrained in one’s belief system to the extent that individuals may not be aware of it. Researchers believe that IM poses a more significant threat to the accuracy of questionnaire results than SDE, as it involves a deliberate distortion of information (Paulhus, 1984, 2002; Paulhus and Vazire, 2007; Vispoel and Tao, 2013).

### The Balanced Inventory of Desirable Responding

Paulhus (1984, 1988, 1991, 1994, 1998) developed the 40-item Balanced Inventory of Desirable Responding (BIDR) with the aim to measure the two distinct dimensions of SDR, IM (20 items) and SDE (20 items). Both the IM and SDE scales have 10 positively keyed items and 10 negatively keyed items that are reverse-scored before calculating the overall score. The IM scale items represent desirable but implausible statements (e.g., “I never take things that do not belong to me”; “I have [not] done things that I do not tell other people about”). Endorsing a high number of these statements may indicate intentional tailoring of responses. SDE items (e.g., “Once I’ve made up my mind, other people can seldom change my opinion”; “I am fully in control of my own fate”) represent a level of overconfidence that does not match levels of the actual abilities. Individuals scoring high on this scale are thought to report unrealistic yet honestly believed positive self-descriptions. Thus, the distinction between these two dimensions is that IM involves intentional manipulation of one’s image to deceive others, whereas SDE involves unconsciously attempting to maintain a positive self-image.

Respondents rate their agreement level with the items on a 5-point (1 = not true, 5 = very true) or a 7-point (1 = totally disagree, 4 = neutral, 7 = totally agree) Likert-type scale. Paulhus suggested a polytomous (continuous) and a dichotomous (i.e., only scores on the end of the scale are counted) scoring method. However, he recommended the use of dichotomous scoring of the 7-point scale (with scores of 6 and 7 being coded as 1 and all other answers coded as 0). These responses are likely to be of the most interest since only extremely high levels of self-deception and impression management are assumed to be abnormal (Paulhus, 1994). Indeed, most research conducted with the BIDR has used the dichotomous scoring method (e.g., Li and Bagger, 2007). However, while limited in number, studies in which dichotomous and polytomous scoring have been compared generally support a polytomous scoring method (Cervellione et al., 2009; Kam, 2013; Stöber et al., 2002; Vispoel and Kim, 2014; but see Asgeirsdottir et al., 2016; Gignac, 2013; Leite and Beretvas, 2005).

Studies with the BIDR have been conducted in a wide variety of community and clinical samples and settings, and the measure is currently one of the most widely used instruments to assess SDR (Leite and Cooper, 2010; Steenkamp et al., 2010). Generally speaking, the scale is a robust measure in forensic/correctional settings as well as in the general population, showing satisfactory reliability (internal consistency; test–retest stability) and convergent and discriminant validity of both IM and SDE (e.g., Lanyon and Carle, 2007; Li and Bagger, 2006; Littrell et al., 2021; Mathie and Wakeling, 2011; Stöber et al., 2002; Vispoel and Tao, 2013; but see also Li and Bagger, 2007). That is, in line with other studies (e.g., Mathie and Wakeling, 2011; Littrell et al., 2021). Paulhus (1994, 1998) reported that BIDR scores have good reliability, with internal consistency estimates ranging from .72 to .75 for SDE and .81 to .84 for IM. Paulhus considered these values highly satisfactory, but some researchers disagree. For instance, in their meta-analysis, Li and Bagger (2007) showed an average reliability of .74 for IM and .68 for SDE, suggesting weaker reliability than Paulhus claimed.

However, results on the factor structure of the BIDR are equivocal (Gignac, 2013; Lanyon and Carle, 2007; Leite and Beretvas, 2005). Although it is acknowledged that the BIDR captures IM and SDE as separate constructs, studies have indicated a two-factor structure of SDE in which SDE can be further divided into the attribution of positive outcomes (Enhancement) and the denial of negative attributes (Denial) (Kroner and Weekes, 1996; Paulhus and Reid, 1991). Moreover, others indicate that both IM and SDE can be split into Enhancement and Denial (Li and Li, 2008; Li et al., 2015).

### Short forms of the Balanced Inventory of Desirable Responding

Due to the non-optimal model fit and the resulting need to remove items as well as the wish to shorten the time needed to administer the instrument to increase its usefulness, several short forms of the BIDR have been developed in recent years. Whereas some short forms have been created using Classical Test Theory (CTT) (Bobbio and Manganelli, 2011; Hart et al., 2015), other attempts have been made using Item Response Theory (IRT) (Asgeirsdottir et al., 2016; Subotić et al., 2016). IRT allows for examining the possibility of multidimensionality on item level to consider how each item is related to SDE and IM. IRT also allows for the instantaneous consideration of the number of endorsed items and item properties (e.g., discrimination, difficulty) when estimating each respondent’s score on SDE and IM (for more information on the difference between CTT and IRT, see Hambleton et al., 1991).

Although the studies using IRT provided valuable information about the performance of a BIDR short form, no study simultaneously considered aspects of multidimensionality and various scoring methods (dichotomous versus polytomous). In addition, IRT analyses can be used to graphically depict the expected item score over the full range of the IM and SDE (i.e., Item Characteristic Curves; ICC) and the amount of information that each item (Item Information Curve; IIC) or the full scale (Test Information Curve; TIC) provides across varying ability levels of IM and SDE (Baker and Kim, 2017; Reeve and Fayers, 2005). ICCs give more detailed insight into possible scoring patterns, especially with regard to polytomous scoring. In addition, for IIC and TIC, the higher the information at specific ability levels of SDE or IM, the lower the associated error (Baker and Kim, 2017). Also, low item information can indicate poorly functioning items (see Reeve and Fayers, 2005). By examining these sources of information, a more informed item selection can be made to create a short form.

Additionally, while several short forms have been developed in various countries (e.g., Asgeirsdottir et al., 2016; Hart et al., 2015; Subotić et al., 2016), only four of the BIDR- items are included in at least seven of the eight short forms that are developed (i.e., Item 11 “I never regret my decisions.,” Item 15 “I am a completely rational person.,” Item 17 “I am very confident of my judgments” (SDE) and Item 37 “I have taken sick-leave from work or school even though I wasn’t really sick” (IM)). Finally, as social desirable behavior is per definition determined by the norms and values within a society, it is advocated to consider the cultural impact on social desirability questionnaires when used in different cultural backgrounds. For instance, research has shown that different cultural values such as individualism/collectivism, masculinity/femininity, and uncertainty avoidance have a diverse influence on SDR (e.g., Bernardi, 2006; Keillor et al., 2001; Middleton and Jones, 2000; see also Li and Reb, 2009; Shulruf et al., 2011).

### The present study

This study was designed to create a short form of the Dutch language version of the BIDR (Version 6) using IRT analysis, aiming to select items with optimal measurement properties suitable for both dichotomous and polytomous scoring methods. In Study 1, we investigated the BIDR’s item qualities using IRT, taking both multidimensionality and scoring method into account, in a community sample. To examine whether the short form is equal to, or even outperforms, the 40-item BIDR (for both scoring methods), we compared the psychometric properties (i.e., internal consistency, test–retest reliability, test information) of the short form and the 40-item BIDR in Study 1. To examine the extent to which the results from Study 1 could be replicated, a second IRT analysis in another community sample was conducted in Study 2. In Study 2 we also investigated the nomological network of the BIDR using both the short form and original version by examining the associations between basic personality features and deviant traits and thoughts and SDE and IM. To establish the validity of our short form, we tested the utility of the short form and the 40-item BIDR as validity scales by investigating whether these scales moderate the convergence between self-reported aggression with informant reports of aggressive behavior in Study 3.

## Study 1

### Methods

#### Participants and procedure

Five-hundred and seventy-seven community participants volunteered to take part in the study between 2016 and 2018. Participants were acquaintances (e.g., neighbors, colleagues, friends) of 30 university psychology students. Participants had to be at least 18 years old and be proficient in the Dutch language to be included in the study. After being introduced to the general aim of the study—Do questionnaires measure what they intend to measure?—participants signed an informed consent form. Participants completed a battery of questionnaires, including topics such as antisocial behavior, psychopathy, major life events, narcissism, and the BIDR. After completion, participants were instructed to send the questionnaires in a sealed envelope to the first author to guarantee anonymity. Participants were also asked to indicate in writing if they would like to participate for a second time. If participants indicated that they were willing to participated a second time, a questionnaire was send by regular mail including a return envelope. Participants were informed that they could withdraw from the study at any time without providing a reason and that their responses would be removed from the database upon request. The study was approved by the School of Social and Behavioral Sciences Ethics Review Board of Tilburg University (ED-2015.70).

Regarding missing values, the total percentage of randomly missing data on the BIDR was 5.7%, with a maximum of two missing values for six participants. Missing values were handled using Full Information Maximum Likelihood. The sample consisted of 577 participants (57.9% male; three participants did not indicate their sex) with an average age of 32.9 years (*SD* = 14.6; range 18–77 years; 26 participants did not report their age). Almost all participants had the Dutch nationality (96.6%; 1.4% missing). Regarding the level of education (0.5% missing), few participants (1.4%) only completed elementary school, 27.4% held a high school degree, 52.1% had a lower or higher vocational education, and 18.5% held a university degree. In total, 10.3% (*n* = 59) indicated having been in contact with the law for various crimes (e.g., traffic violations, vandalism, arson, (sexual) assault). Additionally, 155 participants (26.8%) indicated having received treatment for psychiatric complaints/symptoms.

To examine test–retest reliability, 87 participants (57.5% female) with an average age of 39.3 years (*SD* = 16.4; range 18–77 years) filled out the BIDR for a second time. A sample size of 87 can be considered to be sufficient for test–retest reliability (Kennedy, 2022). Almost all participants reported being of Dutch nationality (97.7%). Regarding the highest education received, 25.3% finished high school, 48.2% had lower or higher vocational education, and 26.4% had a university diploma. The time between the first and second assessments was *M* = 51 days (*SD* = 18.6; range 15–169 days).

#### Measures

For the purpose of this study, the 40-item BIDR Version 6 (Paulhus, 1991, 1994) was translated into Dutch by the first and second author. Two independent bilingual translators conducted a backward translation. Both forward, backward, and final translations were discussed among the authors to reach a consensus when necessary. Translation was done in accordance with the International Test Commission Guidelines for translation and adapting tests (International Test Commission, 2017). Each item was rated on a 7-point Likert-type scale (1 = totally disagree, true, 4 = neutral, 7 = totally agree). When appropriate, items were reverse coded (half of the items). For polytomous scoring, items remained on a 7-point scale, with higher scores indicating higher levels of SDE or IM items. For dichotomous scoring, a score of 1 was assigned to each response of 6 or 7, and a score of 0 was assigned to other responses on the 7-point Likert-type scale.

#### Analytic approach

As stated, IRT is a well-validated method to shorten scales, but it requires a fitting model. The model fit was established for both dichotomous and polytomous scoring. Following previous studies (Asgeirsdottir et al., 2016; Vispoel and Kim, 2014) and recommendations (Paek and Cole, 2020), we used a two-parameter logistic model (2-PL) (Birnbaum, 1968; Lord, 1997) for dichotomous scoring and a graded response model (GRM) (Samejima, 1969, 1997) for polytomous scoring. We wanted to take the possible multidimensionality of both IM and SDE into account (i.e., Denial and Enhancement), hence, in line with previous research (e.g., Asgeirsdottir et al., 2016), we examined whether a one- (IM and SDE separately, respectively) or a two-factor model (further division into Denial and Enhancement for IM and SDE) fit the data best. The two factor model was conducted using an ordinal confirmatory factor model. Additionally, the fit of individual items was evaluated using S-X2 statistics (Kang and Chen, 2008; Orlando and Thissen, 2000, 2003), with resulting *p*-values adjusted for false discovery rates (FDR) (Benjamini and Hachberg, 1995). Furthermore, Yen (1984) Q3 LD statistic was used to check for local independence. The Q3 statistic measures the correlation between performances on two items, after accounting for performance on the overall assessment of SDE or IM (for more information, see Chen and Thissen, 1997).

IRT analyses were used to identify the strongest items for the SDE and IM subscales. First, Factor loadings (e.g., factor analysis parameters) were examined to see how well the items represent the underlying construct. Factor models for ordinal data share a strong connection with Item Response Theory (IRT) models and, in certain cases, can be considered equivalent (Takane and De Leeuw, 1987). This equivalence indicates that the parameters of an IRT model can be transformed into those of a factor model, and vice versa, without loss of information. When the two models are closely aligned, their reparametrized parameters tend to be nearly identical. However, results in our analysis sometimes indicated that the factor loadings and discriminant parameter did classify in different categories when interpreting the findings. To maintain consistency with prior research on the BIDR, we incorporated factor loadings as a key informative component (Asgeirsdottir et al., 2016; Subotić et al., 2016). Second, IRT parameters were examined. Item discrimination parameter (a) estimates were obtained to determine how well each item could identify people at various levels of SDE and IM. Highly discriminating items can discriminate between respondents with subtly different levels of SDE or IM, whereas items that do not discriminate well are only able to discriminate between persons with very different levels of SDE and IM (Reise and Henson, 2003). Complementary to a, the difficulty parameter estimates b (or item location) were examined. Item difficulty describes how high a person typically scores on SDE or IM before an item is endorsed (Reise and Henson, 2003). Finally, the Item Characteristic Curve (ICC) and the Item Information Curve (IIC) were visually inspected for each item. For specific selection criteria, see the result section.

After selecting the items for the short form based on the IRT results (see the result section for specific criteria), Test Information Curves (TIC; i.e., equal to item information) will be interpreted and compared to examine whether the short form performs equally the full 40-item BIDR scale. The TIC is the graphic depiction of the sum of probabilities of endorsing the correct answer for all the items in the measure and therefore estimates the expected test score. A trade-off between the amount of information and the information range is desired: A selection of items that together give a relatively high amount of information over the full range is preferred.

McDonald’s Omega was calculated to measure the internal consistency for each (sub)scale. Test–retest reliability was assessed using Spearman correlations. As the time period between the first and second assessments varied largely (*M* = 51 days, *SD* = 18.6; Range = 15–169 days), moderation analyses were performed to investigate if the time between measurements influences the association between the time points.

All analyses were conducted using R version 3.5.3 (R Core Team, 2017). IRT analyses (including both factor analysis parameters and IRT parameters) were performed using the package Multidimensional Item Response Theory (MIRT; Version 1.3) (Chalmers, 2012). Coefficient Omega was calculated using the package “userfriendlyscience” (Version 0.7.2) (Peters, 2018). Correlational analyses and moderation analyses were conducted using the package Psych (Version, 1.8.12) (Revelle, 2018) and Lavaan (Version 6.3) (Rosseel, 2012), respectively.

### Results

#### Measurement properties at item level using item response theory

Model and Item Fit. The overall model fit of SDE and IM was assessed as follows: absolute, overall model-data fit M2 *p* > .05 for exact fit; bivariate Root Mean Square Error of Approximation (RMSEA2) ≤ .089 for adequate fit, ≤ .050 for close fit, and .050/(number of categories – 1) for excellent fit; and Standard Root Mean Squared Residual (SRMSR) ≤ .060 for adequate fit, ≤ .027 for close fit, and .027/(number of categories −1) for excellent fit (Hu and Bentler, 1999; Maydeu-Olivares and Joe, 2014). To compare relative model fit across different solutions, the nested log-likelihood test (LR) (Hambleton et al., 1991), the Akaike Information Criterion (AIC) (Akaike, 1974), Bayesian Information Criterion (BIC) (Schwarz, 1978), and the sample size adjusted BIC (saBIC) (Sclove, 1987) were used with the lowest values on a specific information criterion being indicative of the better model. As an indication of local dependence, a cut-off of .30 minus the average correlation was used as a critical value as the Q3 is dependent on the sample, the number of items, and scoring method (see Christensen et al., 2017). Additionally, significant S-X2 statistics (Kang and Chen, 2008; Orlando and Thissen, 2000, 2003) indicated item misfit (S-X2, *p* < .05).

Fit statistics indicated that for both SDE and IM, a two-factor solution (i.e., SDE and IM subdivided into Denial and Enhancement) fit the data best for dichotomous scoring (Table 1), with an adequate fit for RMSEA and SRMSR and information-based fit indices indicating the two-factor solution as the better fit. In addition, no indications of item misfit or local dependence were found. For the polytomous scoring method, a two-factor model fit was also considered adequate for both SDE and IM (Table 2) with adequate fit for RMSEA, close to adequate fit for SMRS and information-based fit indices indicating the two-factor solution as the better fit. However, for SDE, item pair 3 (“I do not care to know what other people really think of me”) and 17 (“I am very confident of my judgments”) gave an indication of local dependence for polytomous scoring (Q3 = −.42), suggesting a residual correlation between this item pair beyond the overall construct. The local dependence was possibly the result of item content (for S-X2 statistics and LD Q3 matrix, see Supplementary material S1). In fact, the model fit did improve to an acceptable fit after items 3 and 17 were removed, and no other cases of local dependence were identified. Due to improvement in model fit with all fit indices dropping and information-based fit indices indicating the modified version as best fit for the data, we used the modified two-factor version (excluding items 3 and 17) of the SDE when polytomously scored. Additionally, individual item fit statistics indicated that for IM the model could be improved by removing items 31, 36, and 39 (S-X2, *p* < .05). Therefore, these items were not included in the short version. However, the model fit of the two-factor model did not improve when these items were removed (fit indices and information-based fit indices increased).

**Table 1:** Fit statistics for the IRT models for SDE and IM dichotomous scoring.

Model	Dichotomous 2PL			
	SDE		IM	
	One factor	Two factors	One factor	Two factors
−2LL	−6160.042	**−6119.080**	−6088.515	**−6068.787**
M2 (df)	441.965353 (170)*	315.047632 (169)*	356.40856 (170)*	321.025991 (169)*
RMSEA2 (CI.90%)	.053 (.047, .059)	**.039 (.032, .045)**	.044 (.037, .050)	**.040 (.032, .046)**
SRMSR	.059	**.052**	.052	**.049**
AIC	12400.08	**12320.16**	12257.03	**12219.57**
BIC	12574.40	**12498.83**	12431.34	**12398.25**
saBIC	12447.41	**12368.67**	12304.36	**12268.09**
Estimated parameters	40	41	40	41
−2LLchange	40.962 (1), *p* < .001		19.728 (1), *p* < .001	

−2LL = log-likelihood test. RMSEA2, bivariate root mean square error of approximation; SRMSR, Standardized Root Mean Square Residual; AIC, Akaike information criterion, BIC, Bayesian information criterion; saBIC, sample size adjusted BIC. The one-factor scale represents the unidimensional construct SDE or IM, two factors indicate items of SDE and IM divided in Denial and Enhancement based on reversed and non-reversed items, Modified two-factor model is a two-factor model after correcting for possible local dependence and item misfit (S-X2). *Statistically significant model misfit at *p* < .05. Bold indicates when model fit index indicates the better fitting model.

**Table 2:** Fit statistics for the IRT Models for SDE and IM polytomous scoring.

Model	Polytomous GRM					
	SDE			IM		
	One factor	Two factors	Modified two factor	One factor	Two factors	Modified two factor
−2LL	−20222.100	−20085.330	**−18215.880**	−19840.54	**−19819.54**	−19921.85
M2 (df)	234.855973 (70)*	192.4555328 (69)*	96.093184 (44)*	234.855973 (70)*	192.4555328 (69)*	311.5418137 (72)*
RMSEA2 (CI.90%)	.072 (.064, .081)	.055 (.045, .064)	**.045 (.033, .058)**	.064 (.055, .073)	**.056 (.046, .065)**	.076 (.067, .085)
SRMSR	.067	.063	**.059**	.067	**.063**	.098
AIC	40724.19	40452.67	**36685.77**	39961.09	**39921.08**	40119.7
BIC	41334.29	41067.12	**37239.21**	40571.18	**40535.53**	40721.08
saBIC	40889.85	40619.51	**36836.00**	40126.74	**40087.92**	40282.99
Estimated parameters	140	141	127	140	141	138
−2LLchange	136.77 (1), *p* < .001			21.00 (1), *p* < .001		
		1869.450 (14), *p* < .001			102.31 (3), *p* < .001	

−2LL = log-likelihood test. RMSEA2, bivariate root mean square error of approximation; SRMSR, Standardized Root Mean Square Residual; AIC, Akaike information criterion; BIC, Bayesian information criterion; saBIC, sample size adjusted BIC. Bold indicates when model fit index indicates the better fitting model. The one-factor scale represents the unidimensional construct SDE or IM, two factors indicate items of SDE and IM divided in Denial and Enhancement based on reversed and non-reversed items, Modified two-factor model is a two-factor model after correcting for possible local dependence and item misfit (S-X2). *Statistically significant model misfit at *p* < .05. Bold indicates when model fit index indicates the better fitting model.

#### Item pool selection based on item properties of the item response methods

To identify the strongest SDE and IM items for inclusion in our short form that worked well for both dichotomous and polytomous scoring, we investigated factor loadings, item discrimination parameter estimates (a), and item difficulty parameter estimates (b). Standardized factor loadings < .40 were considered to be low (Floyd and Widaman, 1995), factor loadings .40–.55 to be adequate and factor loadings > .55 were considered to be good in terms of linking to the underlying construct (Comrey and Lee, 1992). Baker and Kim’s guidelines (2017) were used to interpret the item discrimination parameter estimates (a), with values close to 0 indicating no discrimination, values ≤ 0.34 very low discrimination, values 0.35–0.64 low discrimination, values 0.65–1.34 moderate discrimination—which is considered the minimum threshold for discriminating between respondents—and values ≥ 1.35 indicating a high to very high discrimination. For the item difficulty parameters estimates (b) of dichotomous items, it is desirable to create a social desirability measure with a variety of difficulty levels within the higher SDE/IM range, with people higher on the trait having a higher probability of answering affirmative (i.e., no extremely negative b values). For polytomous scoring, six b values are given, indicating the threshold between the seven possible scoring options. These threshold values indicate how high an individuals’ SDE or IM trait level needs to be to have a .50 probability of endorsing this category or a higher response category (Baker and Kim, 2017). It is desirable to obtain items with difficulties spread across the full normal range of SDE and IM, with a lower-bound threshold of one and a higher-bound threshold of six. We aimed at selecting items matching at least the minimum required properties for both scoring methods for IM and SDE.

The following criteria were used for the visual inspection of the ICC and IIC. For polytomous scoring, it is desirable that ICCs are well distributed spread out peaked distributions across response categories (e.g., the probability of giving a score of 5 is on the higher side of the trait, whereas the probability of scoring a 2 is on the lower side of the trait). For dichotomous scoring, the ICC depicts the relationship between the probability of endorsing an item and the level of SDE/IM. The desired item is less likely to be endorsed when a person has a low SDE or IM level than a person with a high trait level and vice versa. In terms of desirable ICC for dichotomous scoring, an S shaped form is preferred with a steep increase at moderate levels of SDE and IM. For IIC, in selecting items for SDE and IM, a trade-off between the amount of information and the information range is desired. A selection of items that together give a relatively high amount of information over the full range is preferred.

##### Self-Deceptive Enhancement

**Figure 1:**
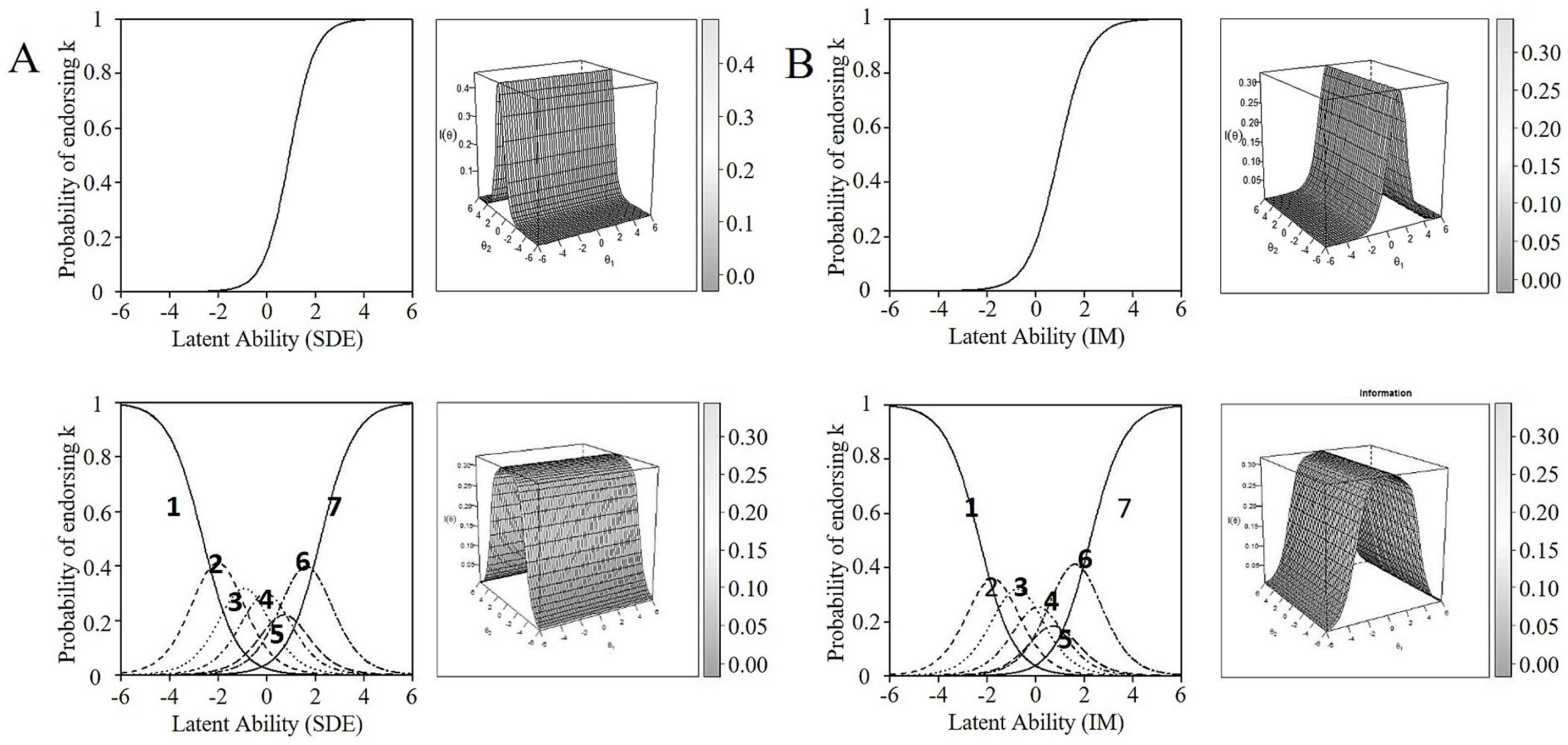
Item Characteristic Curve (ICC) and Item Information Curve (IIC) for item 20 **(A)** and Item 21 **(B)** dichotomous (upper rows) and polytomous scoring (lower rows) of the BIDR 40-item original version.

SDE items with the best trade-off between item properties (see explanation above), for both dichotomous and polytomous scoring, were items 2, 4, 6, 10, 18, 20 (Denial), and 5 (Enhancement) (see Table 3 for item labels and parameter estimator values). That is, these items had factor loadings ranging from .51 to .75, discriminated well between low- and high-levels of SDE with moderate to high discrimination estimates (a range = 1.01–1.91), and demonstrated difficulty estimates in a broad range without the items being too easy or too difficult (Table 3). All items provided relatively higher levels of information at the middle/higher side of the SDE trait. Overall, response categories were endorsed at the appropriate underlying trait level (see, for example, the ICC and IIC of item 20 in Figure 1A; ICCs and IICs of the other SDE items can be found in Supplementary material S1).

**Table 3:** Factor loadings, discrimination, threshold, and difficulty parameters for SDE for dichotomous and polytomous scoring.

Items		Dichotomous scoring					Polytomous scoring									
		Factor loading		*a1*	*a2*	*b*	Factor loading		*a1*	*a2*	Difficulty (*b*)					
		1	2				1	2			1	2	3	4	5	6
Self-deceptive enhancement																
1	My first impressions of people usually turn out to be right.	.32		0.57		1.28	.33		0.60		−7.87	−6.43	−4.57	−1.55	1.28	4.80
3	I do not care to know what other people really think of me.	.63		1.39		1.78										
5	I always know why I like things.	.60		1.27		0.34	.60		1.27		−4.17	−2.61	−1.65	−0.71	0.34	2.00
7	Once I’ve made up my mind, other people can seldom change my opinion.	.33		0.59		1.83	.22		0.38		−9.53	−5.30	−2.34	0.15	2.73	6.85
9	I am fully in control of my own fate.	.61		1.31		−1.20	.47		0.91		−3.17	−2.26	−1.29	0.28	1.50	3.13
11	I never regret my decisions.	.58		1.22		2.21	.36		0.66		−3.34	−0.98	0.65	2.30	3.50	6.26
13	The reason I vote is because my vote can make a difference.	.32		0.58		1.42	.16		0.28		−6.75	−4.88	−3.08	−0.23	2.75	7.21
15	I am a completely rational person.	.62		1.33		1.41	.44		0.83		−2.96	−1.84	−0.87	0.65	1.92	3.90
17	I am very confident of my judgments	.74		1.86		0.95										
19	It is all right with me if some people happen to dislike me.	.40		0.75		1.08	.23		0.40		−7.02	−4.04	−2.29	−0.17	1.86	5.09
2	It would be hard for me to break any of my bad habits.		.51		1.01	1.35		.44		0.82	−4.61	−2.40	−0.79	0.49	1.55	3.05
4	I have not always been honest with myself.		.63		1.51	0.89		.59		1.25	−2.82	−1.62	−0.50	0.34	0.92	2.30
6	When my emotions are aroused, it biases my thinking.		.59		1.23	1.97		.48		0.94	−2.74	−0.95	0.29	1.56	2.34	3.75
8	I am not a safe driver when I exceed the speed limit.		.35		0.63	0.22		.17		0.30	−8.71	−6.11	−3.97	−1.70	0.40	4.14
10	It is hard for me to shut off a disturbing thought.		.59		1.24	1.62		.56		1.15	−2.51	−1.05	0.13	1.00	1.67	3.24
12	I sometimes lose out […] because I cannot make up my mind soon enough.		.53		1.07	0.79		.47		0.89	−4.43	−2.37	−1.03	−0.14	0.87	2.63
14	My parents were not always fair when they punished me.		.43		0.80	−0.27		.36		0.67	−4.99	−3.67	−2.45	−1.16	−0.33	1.37
16	I rarely appreciate criticism.		.31		0.56	0.78		.23		0.40	−14.28	−6.97	−4.16	−1.88	1.03	5.33
18	I have sometimes doubted my ability as a lover.		.54		1.08	0.80		.48		0.92	−3.85	−1.98	−0.83	0.18	0.89	2.19
20	I do not always know the reasons why I do the things I do.		.75		1.91	0.92		.65		1.44	−2.61	−1.39	−0.47	0.39	1.02	2.19

a is the discrimination parameter (or slope). For polytomous scoring, given that each item has 7 item categories, there are six thresholds (i.e., difficulty parameter, b) creating these seven categories. The mirt package in R only provides estimates for intercepts which can be transformed into threshold values for each item using the following formula (−d/a), where d is the intercept value for the corresponding response category and a is the slope for the item. The intercept together with confidence intervals can be found in Supplementary material S1. Darker notation indicate greater factor loadings (< .40; .40–.55; ≥ .55) and discrimination parameters (< 0.65; 0.65–1.34; > 1.34). Items 3 and 17 indicated local dependence for polytomous scoring method.

##### Impression Management

The IM items with the best trade-off between properties for both scoring options (see explanation above) were items 21, 23, 25, 27 (Denial), and 28 (Enhancement) options (Table 4). Factor loadings of these items ranged from .52 to .69 for dichotomous scoring, and from .47 to .64 for polytomous scoring. Items had moderate to high discrimination (a between 0.90 and 1.61), and difficulty levels were in a broader range without being extremely easy or difficult. IICs indicated high(er) levels of information, together covering a relatively broad range of IM (see, for example, the ICC and IIC of item 21 in Figure 1B; ICCs and IICs of the other items can be found in Supplementary material S1). Also, these items showed an overall pattern of a probability of endorsing most response options at the corresponding IM level. In sum, the IRT analyses resulted in a 12-item short form (seven SDE and five IM items) of the BIDR that is suitable for both dichotomous (D) and polytomous (P) scoring. In the following, we will refer to this short form as the BIDR-DP12.

**Table 4:** Factor loadings, discrimination, threshold, and difficulty parameters for IM for dichotomous and polytomous scoring.

Items		Dichotomous scoring					Polytomous scoring									
		Factor loading		*a1*	*a2*	*b*	Factor loading		*a1*	*a2*	Difficulty (*b*)					
		1	2				1	2			1	2	3	4	5	6
Impression management																
21	I sometimes tell lies if I have to.		.69		1.61	0.94		.64		1.41	−2.30	−1.23	−0.28	0.46	0.99	2.24
23	There have been occasions when I have taken advantage of someone.		.69		1.61	0.46		.53		1.06	−4.16	−3.17	−2.03	−1.19	−0.56	0.86
25	I sometimes try to get even rather than forgive and forget.		.60		1.27	−0.11		.49		0.94	−4.55	−2.80	−1.74	−0.81	−0.12	1.25
27	I have said something bad about a friend behind his/her back.		.52		1.05	−1.50		.47		0.91	−2.34	−1.13	0.06	1.04	1.68	3.07
29	I have received too much change from a salesperson without telling him or her.		.51		1.07	1.10		.50		0.99	−1.05	−0.22	0.48	0.90	1.18	1.77
**31**	When I was young I sometimes stole things.		.44		0.83	−0.16		.47		0.91	−1.63	−0.91	−0.42	−0.05	0.21	0.87
33	I sometimes drive faster than the speed limit.		.21		0.37	5.41		.32		0.57	−0.53	0.81	2.12	3.02	3.64	4.47
35	I have done things that I do not tell other people about.		.53		1.05	−1.29		.58		1.21	−1.24	−0.25	0.31	0.85	1.19	1.91
37	I have taken sick-leave from work or school even though I wasn’t really sick.		.43		0.81	1.09		.52		1.05	−0.86	−0.10	0.36	0.75	0.94	1.46
**39**	I have some pretty awful habits.		.47		0.90	−1.81		.45		0.87	−2.27	−1.00	0.13	1.20	1.87	3.47
22	I never cover up my mistakes.	.51		1.01		2.03	.34		0.62		−4.66	−1.89	0.06	1.72	2.98	5.16
24	I never swear.	.35		0.63		4.22	.50		0.99		0.18	1.11	1.74	2.32	2.89	4.43
26	I always obey laws, even if I’m unlikely to get caught.	.46		0.88		1.05	.49		0.95		−2.74	−1.28	−0.39	0.24	0.97	2.73
28	When I hear people talking privately, I avoid listening.	.52		1.03		1.72	.47		0.90		−2.48	−0.77	0.16	1.15	1.87	3.24
30	I always declare everything at customs.	.31		0.55		0.04	.26		0.46		−4.15	−2.86	−2.13	−0.82	0.10	1.48
32	I have never dropped litter on the street.	.52		1.03		0.20	.53		1.06		−0.30	0.56	1.09	1.54	1.94	2.96
34	I never read sexy books or magazines.	.40		0.74		0.76	.35		0.63		−2.09	−0.91	−0.25	0.55	0.88	2.04
**36**	I never take things that do not belong to me.	.63		1.39		−0.24	.45		0.86		−3.16	−2.17	−1.42	−0.77	−0.34	0.77
38	I have never damaged a library book or […] without reporting it.	.60		1.27		−0.33	.40		0.75		−2.64	−1.97	−1.43	−1.06	−0.48	0.47
40	I do not gossip about other people’s business.	.44		0.83		1.93	.38		0.70		−3.52	−1.35	0.02	1.35	2.24	4.62

a is the discrimination parameter (or slope). For polytomous scoring, given that each item has 7 item categories, there are six thresholds (i.e., difficulty parameter, b) creating these seven categories. The mirt package in R only provides estimates for intercepts which can be transformed into threshold values for each item using the following formula (−d/a), where d is the intercept value for the corresponding response category and a is the slope for the item. The intercept together with confidence intervals can be found in Supplementary material S1. Darker notation indicate greater factor loadings (< .40; .40–.55; ≥ .55) and discrimination parameters (<0.65; 0.65–1.34; > 1.34); Items **31, 36, 39** where indicated as misfitting (sign. S-X2 statistics) for polytomous scoring.

#### Dichotomous versus polytomous scoring method

**Figure 2:**
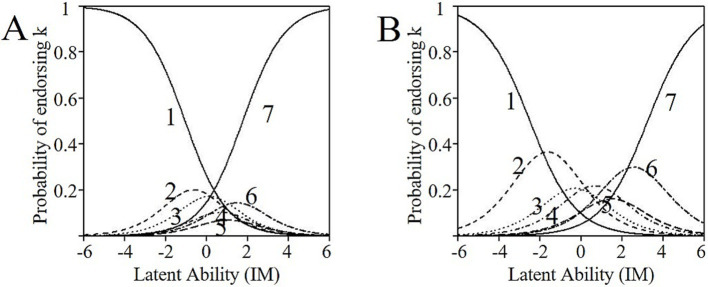
Item Information Curve (IIC) for item 29 **(A)** and item 29 **(B)** polytomous scoring of the BIDR 40-item original version.

Although the primary goal of the study was to create a short form of the BIDR for both dichotomous and polytomous scoring, which led to the proposed BIDR-DP12, our results indicated that the dichotomous scoring method was better than the polytomous scoring in terms of model fit, factor loadings, and IRT item properties (i.e., a, b, ICC, IIC). Moreover, for the polytomous scoring method, all BIDR items were often more or less answered with a more skewed answering tendency (i.e., most of the time, a score of 1–2 or 6–7 was given). Especially for IM, although the items with the best parameter trade-off were selected, ICCs indicated a skewed answering tendency for more than half of the items (see item 29 in Figure 2A, for an example). Besides, the probability of selecting some answering categories was extremely low as indicated by flat and overlapping ICCs (i.e., the ICC of some answer categories was below all other answer category ICCs; see item 28 in Figure 2B for an example). This could indicate that a 7-point scale may not be the most suitable solution. Furthermore, factor loadings and discrimination estimates appeared to be lower and sometimes more unfavorable for the polytomous scoring method, which resulted in the elimination of some items with good item parameter properties when dichotomously scored (e.g., item 22, see Table 4). Regarding SDE, some items with good item parameter properties were not selected due to more unfavorable parameters for polytomous scoring method (see, for example, item 11 in Table 3).

Thus, our 12-item short form that is suitable for both dichotomous and polytomous scoring resulted in a loss of items when used polytomously. Moreover, ICCs indicated that the polytomous scoring method resulted in a strongly skewed distribution leading to a comparison of participants in one tail of the distribution versus the others. This might indicate a more or less dichotomous answering tendency. Therefore, we also decided to create a short form for dichotomous scoring only. Based on factor loadings, estimator parameters, ICCs, and IICs, we included the SDE items 4, 6, 10, 18, 20 (Denial) and 3, 5, 9, 15, 17 (Enhancement) and the IM items 21, 23, 25, 29, 35 (Denial), and 22, 28, 32, 36, 38 (Enhancement) in this 20-item ‘dichotomous only’ short form, which we named the BIDR-D20.

#### Test information, internal consistency, and test–retest reliability

**Figure 3:**
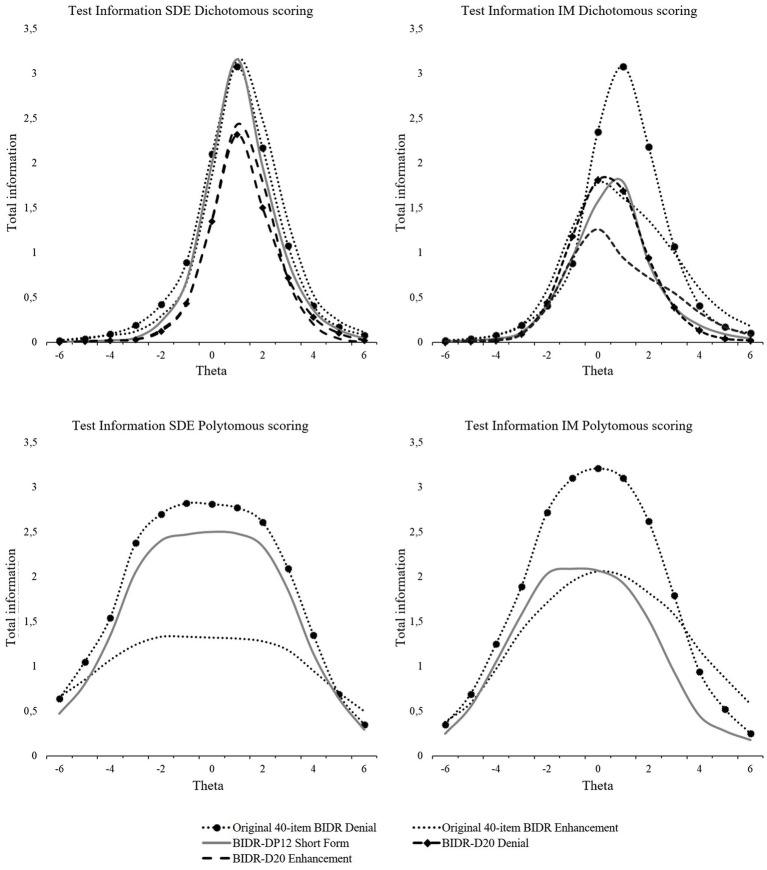
Test information functions for SDE and IM dichotomous and polytomous scoring.

Test information indicated that the original BIDR provided the most information for both the subscales Denial and Enhancement (Figure 3). This is not surprising as the test information is generated by aggregating the item information, and therefore the original 40-item BIDR was expected to have the highest test information. The dichotomous scoring method provided the strongest information at medium-to-high levels of SDE and IM, but weak to no information for discriminating among respondents at low levels. Polytomous scoring provided information over a broader range of SDE and IM than dichotomous scoring, but information levels were generally lower than the peak level information of the dichotomous scoring method.

Although analyses indicated that, within the IRT framework, both SDE and IM had the best model fit as a two-factor model (i.e., Denial and Enhancement), in clinical practice and research, IM and SDE are rarely further divided into Denial and Enhancement scales. For this reason, all further analyses were also conducted using IM and SDE as one-factor constructs. Internal consistency was considered adequate for most versions; five of the 22 different versions of the BIDR (sub)scales (original both polytomous, and dichotomous, for denial and enhancement; the BIDR-DP12 both versions and BIDR-D20 denial and enhancement; all for IM and SDE) had an McDonald’s Omega between .60 and .70 on both time measures. Omega was lower for polytomous scoring than for dichotomous scoring. The same held for Denial and Enhancement, respectively. Test–retest correlations for the SDE and IM short form(s) and the original BIDR ranged from .61 to .85, which is in line with previous research (e.g., Paulhus, 1991; Hart et al., 2015). After correcting for multiple testing, moderation analyses showed that the time between the Wave 1 and Wave 2 assessment did not affect BIDR scoring.

Correlations across the different versions of SDE or IM at Wave 1 and Wave 2 were moderate to high for SDE (*r* = .62–.88) and for IM (*r* = .62–.90), except for the correlations across the Denial and Enhancement subscales of SDE (*r* = .22–.39) and IM (*r* = .33–.47). Correlations between SDE and IM for the same BIDR version (e.g., BIDR-D12 SDE and BIDR-D12 IM) were small to medium (r between .07 and .45). Omega, test–retest and correlations between IM and SDE, together with the mean and standard deviations of all studies, can be found in Supplementary material S2.

### Discussion

The goal of Study 1 was to create a Dutch short form of the BIDR Version 6. Based on the results of IRT analyses, a short form containing seven SDE and five IM items that can be used for both dichotomous and polytomous scoring methods was proposed, the so-called BIDR-DP12. However, the IRT analyses indicated that responses in the polytomous scoring method tended to be skewed, implying that participants often responded in a more dichotomous fashion rather than utilizing the full range of options. While such skewness is not inherently problematic as it may simply reflect items that are particularly easy or difficult—our objective was to design a questionnaire that made full use of a 7-point Likert scale. Ideally, Item Characteristic Curves (ICCs) would show a balanced distribution across response categories, with higher scores more likely at the upper end of the trait and lower scores at the lower end. In addition, analyses indicated that dichotomous scoring resulted in a better fit in terms of model fit and factor loadings and was more suitable for distinguishing between participants scoring high versus low on SDE and IM. Further, the BIDR-DP12 consisted mostly of items that deny negative attributes (both SDE and IM included only one item of the Enhancement subscale). Subsequently, this resulted in a one-factor model for both SDE and IM. To overcome the aforementioned limitations and create a balanced scale, we also created a ‘dichotomous only’ short form, which we named the BIDR-D20, that includes the best SDE and IM items in terms of IRT parameters, with an equal number of items for Denial and Enhancement.

Contrary to previous research (Stöber et al., 2002; Vispoel and Tao, 2013), dichotomous scoring was better or equal in terms of internal consistency for all BIDR forms. In terms of test–retest reliabilities, however, polytomous scoring produced somewhat better results. This is in line with previous outcomes (Schnapp et al., 2017; Stöber et al., 2002). Comparing our short forms with the 40-item BIDR, it can be concluded that the dichotomously scored BIDR and the BIDR-D20 are comparable in terms of internal consistency and test–retest reliability. The BIDR-D20, however, provided less information over the same range as the full BIDR, which is not unexpected. In general, as the TIC is generated by aggregating the item information, the original version (i.e., longer test) can test an examiner’s ability with greater precision than the shortened versions. To confirm the item and test psychometric properties of the short forms, further examination and replication of the results is needed.

## Study 2

In Study 2, we further examined the psychometric properties of the BIDR-DP12 and BIDR-D20 short forms. First, we repeated IRT analyses in a new community sample to investigate to what extent the item properties of both short forms could be replicated. Second, we aimed to replicate prior research that outlined the nomological network of SDR by testing the associations between SDE, IM, and basic personality traits and deviant traits and thoughts. Research has indicated that higher levels of SDE are associated with lower levels of wself-reported negative emotionality and higher levels of extraversion, open-mindedness, and emotional stability. More specifically, positive correlations have been reported between the SDE Enhancement subscale, self-reported extraversion, and open-mindedness, whereas SDE Denial has been positively associated with self-reported emotional stability, conscientiousness, and agreeableness (for a meta-analysis see Li and Bagger, 2006).

Conversely, higher levels of IM have been associated with higher self-reported agreeableness, conscientiousness, and emotional stability (Holden and Passey, 2010; Li and Bagger, 2006; Meston et al., 1998; Paulhus, 1988, 2002). Research has also suggested that honesty-humility was the most prominent personality factor in explaining SDR (De Vries et al., 2014; Zettler et al., 2015). Regarding the scoring method, Stöber et al. (2002) found in a student population sample that continuous SDE scores demonstrated significantly higher correlations with conscientiousness [*r* = −.51 vs. r = −.31, *z*(diff) = −3.54, *p* < .001], extraversion [*r* = .17 vs. *r* = .03, *z*(diff) = 2.21, *p* < .05], and negative emotionality [*r* = .41 vs. *r* = .27, *z*(diff) = 2.28, *p* < .05] than dichotomous scoring.

In addition, a recent meta-analysis found a significant, albeit small, negative association between IM and SDE on the one hand and self-reports measuring antisocial cognitions (e.g., entitlement to sex) and antisocial personality patterns/traits (e.g., psychopathy traits, antisocial behavior), suggesting that higher scores on IM and SDE are associated with lower scores on self-reports measuring these dynamic risk factors in samples of men who have offended (Hildebrand et al., 2018). Since both IM and SDE seem to be positively correlated with age, and women tend to score higher on IM, whereas men score higher on SDE (Bobbio and Manganelli, 2011; Kroner and Weekes, 1996; Li et al., 2011; but see Hildebrand et al., 2018; Mathie and Wakeling, 2011), both age and sex will be taken into account in the analyses. Expectations of the correlations (i.e., positive or negative) can be found in Table 5 in the result section.

**Table 5:** Correlations for gender, age, personality, risk factors for deviant traits and thoughts and all forms of the BIDR.

Self-deceptive enhancement													
Variables	Expectations				BIDR original-D	BIDR original-D Denial	BIDR original-D Enhanc.	BIDR original-P	BIDR original-P Denial	BIDR original-P Enhanc.	BIDR-D20	BIDR-D20 Denial	BIDR-D20 Enhanc.
	General	Denial	Enhance-ment	ω [95% CI]	.83 [.82, .85]	.57 [.53, .62]	.65 [.61, .69]	.64 [.60, .68]	.61 [.57, .66]	.67 [.63, .70]	.79 [.77, .81]	.71 [.68, .75]	.73 [.70, .77]
Age	+				**.17**	**.14**	**.16**	**.15**	**.11**	**.13**	**.22**	**.25**	**.14**
Gender	−				**−.21**	**−.11**	**−.22**	**−.21**	**−.10**	**−.23**	**−.26**	**−.12**	**−.27**
Extraversion	+		+		**.23**	**.21**	**.17**	**.34**	**.26**	**.25**	**.16**	**.16**	**.11**
Agreeableness		+			**.15**	**.20**	.07	.08	**.13**	−.01	**.12**	**.12**	**.10**
Conscientiousness	+	+			**.23**	**.28**	**.12**	**.29**	**.30**	**.13**	**.20**	**.24**	**.12**
Negative emotionality	−	−			**−.46**	**−.45**	**−.32**	**−.59**	**−.49**	**−.39**	**−.49**	**−.47**	**−.35**
Open-mindedness	+		+		**.22**	**.12**	**.24**	**.15**	.05	**.18**	**.21**	**.13**	**.20**
Honesty humility					**.20**	**.25**	**.10**	**.17**	**.21**	.06	**.16**	**.22**	**.08**
Antisocial behavior	−				−.02	−.06	.02	−.03	**−.09**	.05	−.02	−.03	−.01
Primary psychopathy	−				**−.06**	**−.12**	.00	.01	−.07	**.08**	−.04	**−.11**	.01
Secundaire psychopathy	−				**−.25**	**−.33**	**−.11**	**−.28**	**−.34**	**−.09**	**−.24**	**−.28**	**−.16**
Sexual entitlement	−				**−.11**	**−.14**	−.05	−.04	**−.09**	.03	**−.10**	**−.12**	−.06
Impression management													
	General	Denial	Enhance-ment	ω [95% CI]	.89 [.88, .90]	.82 [.81, .84]	.83 [.81, .85]	.78 [.76, .81]	.72 [.69, .75]	.68 [.64, .71]	.83 [.82, .85]	.76 [.73, .79]	.74 [.71, .77]
Age	+				**.24**	**.22**	**.19**	**.22**	**.18**	**.19**	**.29**	**.26**	**.19**
Gender	+				**.12**	**.11**	**.11**	**.17**	**.16**	**.14**	**.10**	**.14**	**.11**
Extraversion					−.01	−.03	.01	**−.03**	−.04	−.01	.02	.01	.01
Agreeableness	+				**.35**	**.28**	**.34**	**.33**	**.24**	**.33**	**.32**	**.29**	**.34**
Conscientiousness	+				**.34**	**.30**	**.31**	**.37**	**.32**	**.33**	**.32**	**.27**	**.31**
Negative emotionality	−				**−.15**	**−.17**	**−.10**	**−.13**	**−.09**	**−.12**	**−.17**	**−.15**	**−.10**
Open-mindedness					**.16**	**.10**	**.19**	**.08**	.04	**.12**	**.17**	**.10**	**.19**
Honesty humility	+				**.46**	**.43**	**.38**	**.44**	**.38**	**.37**	**.44**	**.40**	**.38**
Antisocial behavior	−				**−.42**	**−.38**	**−.35**	**−.49**	**−.45**	**−.39**	**−.36**	**−.31**	**−.35**
Primary psychopathy	−				**−.47**	**−.39**	**−.44**	**−.44**	**−.33**	**−.43**	**−.44**	**−.36**	**−.44**
Secundaire psychopathy	−				**−.33**	**−.30**	**−.27**	**−.31**	**−.24**	**−.29**	**−.30**	**−.28**	**−.27**
Sexual entitlement	−				**−.24**	**−.22**	**−.20**	**−.18**	**−.17**	**−.15**	**−.22**	**−.21**	**−.20**

Gender is a dummy variable with being female serving as the reference group. Negative emotionality (i.e., former Neuroticism), and Open-Mindedness (i.e., former Openness to experiences). Enhanc., Enhancement; D, Dichotomous scoring; P, Polytomous scoring; BIDR-D20, 20 item short version that can only be used Dichotomous. Significant values (*p* ≤ .05) are in bold. N ranges between 685 and 719.

### Methods

#### Participants and procedure

An anonymous questionnaire link was distributed to recruit participants from the general population via social media platforms (Linked-In, Facebook, Instagram) in 2018–2019. The sample consisted of 946 participants from the general community. Of those, 719 completed the BIDR; 37.8% of them were male, and the average age was 31.4 years (*SD* = 14.4; 5 missing). Regarding the highest educational background of the participants who completed the BIDR, 1% finished elementary school, 28.4% finished high school, 48.4% lower or higher vocational education, and 22.3% finished a university degree. Almost all participants had the Dutch nationality (94.5%). Participants who completed the BIDR (*n* = 719) did not differ from participants who did not fill out the BIDR (*n* = 227; ranging from 44 to 226 in following analyses due to missing values in the other self-report measures) on their scores on the Big Five Inventory-2 domains (*p*s ≥ .132), psychopathy scale scores (*p*s ≥ .343), antisocial behavior (*p* = .350) or sexual entitlement (*p* = .234). However, those who completed the BIDR were slightly older (*M* = 31.40, *SD* = 14.4) than participants who did not (*M* = 28.8, *SD* = 13.3), *t*(2.502) = 405.39, *p* = .013, and had higher scores on the honesty-humility personality scale (*M* = 35.4, *SD* = 6.0 versus *M* = 33.4, *SD* = 4.2), *t*(3.010) = 54.82, *p* = .004. A weak association was found between filling out the BIDR (37.8% males) and gender [BIDR χ^2^ (1, *N* = 946) = 4.68, *p* = .031, ø = −.070], with more male participants (80%) completing the BIDR than females (74%).

Data collection was conducted via the Qualtrics Internet survey platform. Participants volunteered to participate and signed an informed consent form. The study was approved by the School of Social and Behavioral Sciences Ethics Review Board of Tilburg University (ED-2015.70).

#### Measures

##### BIDR

The items of the BIDR-40 (Version 6) were used and subsequently both short forms (BIDR-DP12 and BIDR-D20), described and investigated in Study 1, were derived from these items.

##### Big Five Inventory-2

The BFI-2 (Soto and John, 2017) is a measure of the Big Five personality domains: Extraversion, Agreeableness, Conscientiousness, Negative Emotionality, and Open-Mindedness. The BFI-2 consists of 60 items equally distributed over the five domains, which are scored on a 5-point Likert scale (1 = strongly disagree, 5 = strongly agree). Research by Soto and John (2017) indicated that the internal consistency of the scales is good, ranging from *α* = .83 (Agreeableness) to α = .90 (Negative Emotionality). The Dutch version of the BFI-2 has comparable internal consistencies (Denissen et al., 2019). The same holds for the current study (α ≥ .80).

##### Honesty-Humility subscale of the HEXACO

The HEXACO (Ashton and Lee, 2008; Dutch translation: De Vries et al., 2008) is a questionnaire consisting of 60 items measuring six personality dimensions. Items are scored on a 5-point Likert scale (1 = completely do not agree; 5 = completely agree). In the current study, only the dimension Honesty-Humility (10 items) was used. Higher scores on this dimension indicate unwillingness to manipulation or take advantage of others, feeling little temptation to break the rules, a relative lack of interest in gaining or feeling entitled to status, whether social of monetary. Internal consistency of the Honesty-Humility scales ranged from .74 to .92 in previous studies (Ashton and Lee, 2008; Ashton et al., 2014; De Vries et al., 2008). In the current study, internal consistency was adequate (α = .70).

##### The Antisocial Behavior Questionnaire

The ASBQ (based on Moffitt and Silva, 1988) is a 29-item self-report questionnaire measuring antisocial behavior. Participants indicated whether they had partaken in antisocial activities such as fighting, stealing, or selling drugs since the age of 18. The items were scored as (0) no, never, (1) 1 time, (2) two or three times, (3) four to six times, and (4) seven or more. A higher score indicates more antisocial behavior. The ASBQ in the current study consisted of 26 items because items regarding whether someone performed antisocial behavior alone or with others were not included due to the purpose of the study. The internal consistency in the current study was .87, which is comparable with other studies (e.g., Sijtsema et al., 2015).

##### Levenson Psychopathy Scale

The LSRP (Levenson et al., 1995; Dutch translation: Uzieblo et al., 2006) is a 26-item self-report questionnaire used to measure psychopathic traits in non-institutionalized samples. Sixteen items (e.g., callous, manipulative, selfish, and deceitful) measure primary psychopathy traits and 10 items (e.g., impulsive, hostile, neurotic) secondary psychopathy. Items were scored on a 4-point Likert scale (1 = completely disagree, 4 = completely agree). Higher scores indicate a higher level of primary or secondary psychopathic traits. Seven items are reversed to control for response sets. Internal consistency was comparable with previous studies (e.g., Levenson et al., 1995; Hicklin and Widiger, 2005) with an internal consistency of α = .83 for primary psychopathy and α = .66 for secondary psychopathy.

##### Sexual Entitlement subscale of the Sexual Narcissism Scale

For measuring sexual entitlement, the 5-item Sexual Entitlement subscale of the SNS (Widman and McNulty, 2010) was used. The SNS is a self-report questionnaire containing 20 items, which can be scored on a 5-point Likert scale (1 = strongly disagree, 5 = strongly agree). The items in the subscale Sexual Entitlement measure the degree of an individual’s sense of sexual entitlement and the idea that meeting one’s sexual desires is a right (e.g., “I am entitled to sex on a regular basis”). The higher the score on the subscale, the greater the level of sexual entitlement. All items were translated into Dutch by the first and third authors, and an independent person did a back translation to confirm that the Dutch translations corresponded with the original English items. Internal consistency of the Sexual Entitlement subscale in this study was satisfactory (α = .77) and comparable with previous research (α = > .76; Widman and McNulty, 2010).

#### Analytic approach

IRT analyses were performed to investigate to what extent the item properties of the BIDR-DP12 and the BIDR-D20 were replicable. We followed the same procedures as in Study 1. Regarding model fit, for the BIDR-DP12 SDE and IM scales, a one-factor solution was used as both scales include only one item of the Enhancement scale. For the BIDR-D20, we compared a one and two-factor solution.

Bivariate correlation analyses using listwise deletions were conducted to examine the associations between the 40-item BIDR, both BIDR short forms, BFI-2 domains, Honesty-Humility, antisocial behavior, psychopathy (primary and secondary), and sexual entitlement. Due to the non-normality of the dichotomous scoring of the short forms and the risk factors for deviant behavior, Spearman correlations were calculated.

Mean scores for the BFI-2 domains, Honesty-Humility, ASBQ, LSRP subscales, and sexual entitlement were calculated when at least 50% of the items were answered (0.7% of the data had missing values, maximum two missing’s per scale/domain). In total, 8.2% of the participants had missing data on more than 50% of the items on one or more of these scales. For this reason, the number of participants varies across analyses.

All analyses were conducted using R version 3.3.3 (R Core Team, 2017). IRT analyses were done using the package MIRT Version 1.3 (Chalmers, 2012), and correlation analyses were conducted using the package Psych (Version, 1.8.12) (Revelle, 2018).

### Results

#### Measurement properties at item level of the short forms

##### Model and item fit

Regarding the BIDR-DP12, analysis indicated that LD was detected for polytomous scoring method and one item indicated misfit for dichotomous scoring. For the polytomous scoring method, removing the items did not result in a better solution. In addition, ICCs of the BIDR-P12 again indicated that some answer options never had the highest probability of being chosen by participants with certain latent trait scores, as indicated by flat, non-distinct and overlapping response category curves. Due to the fact that results from IRT analyses in both Study 1 and 2 indicated that polytomous scoring of the BIDR might not be the most suitable method, as indicated by poor(er) model fit, lower parameter estimates, and ICC that indicate a skewed possibly more dichotomous answering tendency or at least not a 7-point answering inclination, we decided to continue analyses only for the BIDR-D20 short form. For readers interested in the further results of the BIDR-DP12, can contact the first author.

##### BIDR-D20

For both the BIDR-D20 SDE and IM scale, a two-factor model for both SDE (Denial and Enhancement) and IM (Denial and Enhancement) fit the data best with an adequate absolute fit (RMSEA and SRMSR < .50). There were no indications of local dependence or item misfit (see Supplementary material S3 for model and item fit statistics, and the local dependence matrix). For both SDE and IM factor loadings ranged from .41 to .71. Items had satisfactory to good abilities to discriminate (a) between people high and low on both traits (average a SDE = 1.29, IM = 1.42), and displayed a variety of difficulty levels without extreme items, with IM items being somewhat easier, and relatively high(er) information levels in the middle to higher range. The probability of endorsing the items (i.e., a score of 1) increased for most of the items around the average level of theta, with some items having a higher probability at lower levels and some at higher levels (for factor loadings, parameter estimates, ICCs and IICs; see Supplementary material S3).

**Figure 4:**
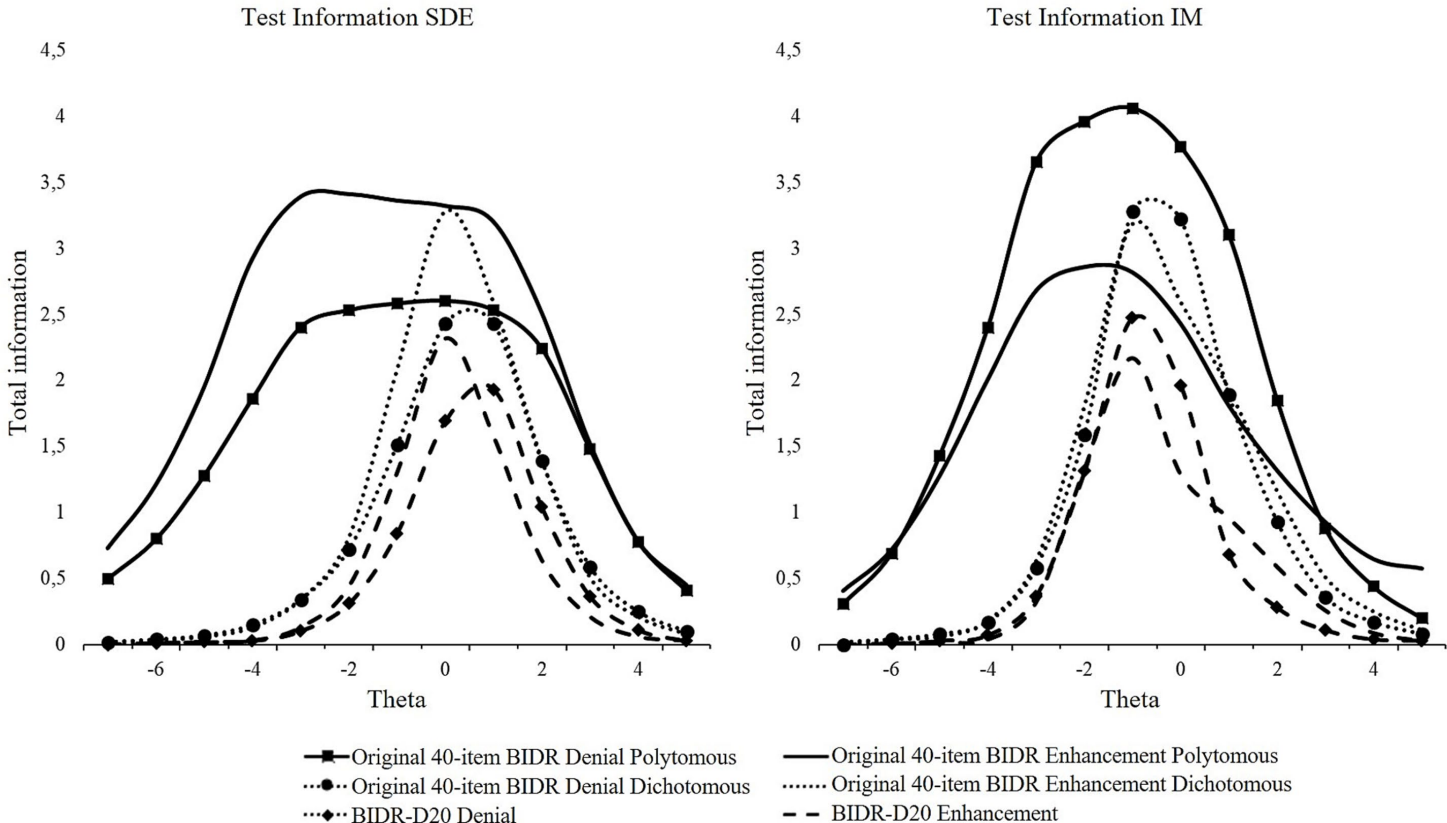
Test information curves for SDE and IM dichotomous scoring.

Coefficient Omega can be found in Table 5. The BIDR-D20 performed equal to and, in some occasions, even better than the original BIDR when dichotomously scored. With regard to the test information, as expected the BIDR-D20 provided less information than both scoring methods of the 40-item BIDR. Similarly as for the dichotomous scoring of the 40-item BIDR, the dichotomous BIDR-D20 provided information at the middle-high range of SDE and IM (see Figure 4).

#### Nomological network of the BIDR-D20 and the BIDR (Version 6)

Overall, the nomological network of the short form did not diverge from that of the 40-item BIDR (Table 5). In line with our expectations, older people scored higher on IM and SDE on both BIDR forms. Moreover, women scored lower on SDE, but overall higher on IM. For personality traits, all expectations were met. In general, higher levels of SDE were associated with lower levels of negative emotionality and higher levels of extraversion and open-mindedness. Though higher levels of IM were associated with higher agreeableness, conscientiousness, and honesty-humility, and lower levels of negative emotionality, most associations were similar for SDE Denial and Enhancement subscales. However, Denial had a stronger positive association with conscientiousness and negative with negative emotionality, whereas Enhancement was more positively associated with open-mindedness. Both subscales were positively associated with extraversion. The unexpected association between SDE and agreeableness can be explained by the positive association with Denial. Though not hypothesized, SDE was associated with higher levels of honesty-humility. Moreover, IM was associated with higher levels of open-mindedness (except the IM Denial subscale, polytomous scored).

Mixed results were found for the association between SDE and the risk factors for deviant behavior. For antisocial behavior, only a significant negative association was found for the Denial subscale of the BIDR when polytomously scored. All associations with secondary psychopathy were significantly negative as expected, yet for primary psychopathy, only a significant association was found with lower levels of the Denial subscales when scored dichotomously, and higher levels of the polytomously scored Enhancement subscale. For sexual entitlement, a significant negative association was found with the full and short dichotomously scored BIDR and the dichotomously scored Denial subscales.

For IM, all expectations were met. Higher levels of IM were associated with lower levels of self-reported antisocial behavior, primary and secondary psychopathy, and sexual entitlement.

### Discussion

The goals of Study 2 were to replicate the findings from Study 1 with regard to the IRT analyses and to investigate whether the 40-item BIDR and our newly created short form(s) performed equally well with regard to their nomological network. Results indicated that the model fit for the polytomous scoring method of the BIDR-DP12 was not satisfactory. In addition, ICCs indicated that a 7-point scale was not the most suitable scoring method. Some answer categories could be dropped because nowhere along the continuum were these categories more likely to be chosen than others. Other ICCs were characterized by flat and non-distinct response category curves. However, for the dichotomous-only short form (BIDR-D20), replicable satisfactory parameter estimates, ICCs, and IICs were found. In addition, the BIDR-D20 performed equal to, and sometimes even outperformed, the scoring methods of the 40-item BIDR in terms of Omega and nomological network. Associations with Big Five personality traits and the deviant traits or thoughts were of the same magnitude and mostly in the expected direction for both scoring methods of the 40-item BIDR as well as the dichotomous BIDR-D20.

To conclude, the BIDR-D20 seems to be a good alternative for the 40-item BIDR in terms of test characteristics, psychometric properties and nomological network. However, for a social desirability measure to be of real value in practice, it should be clear how the measure should be used and what is actually measured: A response bias or a more enduring quality.

## Study 3

In Study 3, we examined the utility of IM and SDE to improve the validity of measures of socially deviant behavior, such as aggression. By using an undesirable behavior that could be rated by others, e.g., observable aggressive behavior, a criterion independent of response bias can be investigated (e.g., informant reports). Using a criterion independent of response bias is critical and often overlooked (see Burchett and Ben-Porath, 2019) in the debate about the role of SDR in assessment and research.

Although SDR is considered importance in (clinical) assessment, the question of how SDR measures should be used is still open for discussion. Some researchers consider SDR to be a short-term response set influenced by situational factors, making it a problematic source of bias (e.g., Edwards, 1957; Nederhof, 1985). Others view SDR as a more enduring trait (e.g., Paulhus, 2002). In support of this, Uziel (2010) proposed that SDR represents the ability to adapt to social situations and seek approval from others (i.e., “interpersonally oriented self-control”). Some researchers advocate adjusting individual scores based on SDR scores (e.g., van de Mortel, 2008), whereas others indicate that this would remove significant variance (e.g., Uziel, 2010).

Theoretically, to be considered a tendency to deceive in self-reports—whether deliberate or unconscious—IM and SDE should affect the correlation between self and informant scores (McCrae and Costa, 1983). That is, someone with a high IM or SDE score can be expected to have low levels of self-reported aggression; yet for many of these cases, informant-reported aggression would be higher than self-reported aggression. Statistically, controlling for the variance associated with IM and SDE should then unveil a more accurate self-report and informant-report correlation (Uziel, 2010). Hence, the association between self-reported aggression and informant-reported aggression is expected to increase when controlling for IM and SDE. By that same logic, a decrease would indicate that IM and SDE have substantive meaning and are not to be considered a response bias. In addition, if SDE and IM are a response bias, it is expected that lower levels of self-reported aggression are related to higher levels of informant-reported aggression, but only at higher levels of SDE or IM. Hence, we would expect a stronger association between self- and informant-reported aggression at low levels of SDE and IM.

### Methods

#### Participants and procedure

As part of a more extensive study, participants were invited to the lab at the university to complete a variety of questionnaires and to conduct several computer tasks about several topics, including aggression. Data collection took place in 2019. Participants were also asked to list the email addresses of family members, friends, or other meaningful members of their social network (with a maximum of five informants per participant), who were subsequently invited to fill out an online version of the Aggression Questionnaire (AQ) (Buss and Perry, 1992) about the participant. All informants received a personalized email referring to the participant by name, asking to rate the participant’s aggression using the AQ. After being introduced to the aim of the study, participants signed an informed consent form and participated voluntarily. Participants were informed that they could withdraw from the study at any time without providing a reason and that their responses would be removed from the database upon request. The study was approved by the School of Social and Behavioral Sciences Ethics Review Board of Tilburg University (EC-2016.39).

Of the total sample of 111 male participants, 100 participants had one or more informant reports with an average of 2.8 reports (SD = 1.4; range 1–5). The average age of these 100 participants was 31.3 years (*SD* = 14.5; range 18–65; 1 missing), and almost all reported having Dutch nationality (94%). The highest education was as follows: 1% completed elementary school as the highest education, 36% high school, 45% low or high vocational education, and 18% completed a university degree. Regarding the informant reports, a total of 282 informant reports were administered. Informants (45.7% male) included a variety of people (e.g., grandfather, father, neighbor, friend, partner), with an average age of 34.6 years (*SD* = 16.5; 17–87 years).

#### Measures

##### BIDR

The items of the BIDR-40 (Version 6) were used and subsequently the items from the BIDR-D20 were derived from this version. Coefficient Omega can be found in Table 6.

**Table 6:** Correlational analyses between aggression (self-reported, informant-reported), SDE and IM original BIDR and BIDR-D20 (*N* = 100).

Variables		ω [95%CI]	Self-reported aggression	Informant-reported aggression
	Informant reported aggression		.54**	
SDE	BIDR original dichotomous	.77 [.71, .83]	−.03	.06
	Denial	.76 [.70, .83]	−.17	−.04
	Enhancement	.63 [.52, .73]	.13	.16
	BIDR original polytomous	.66 [.56, .75]	−.14	−.02
	Denial	.63 [.52, .73]	−.24*	−.10
	Enhancement	.44 [.27, .61]	.04	.09
	BIDR-D20	.76 [.69, .83]	−.09	.03
	Denial	.76 [.69, .83]	−.23*	−.06
	Enhancement	.61 [.49, .73]	.08	.11*
IM	BIDR original dichotomous	.87 [.83, .91]	−.33**	−.20*
	Denial	.76 [.69, .83]	−.44**	−.25*
	Enhancement	.83 [.79, .88]	−.14	−.09*
	BIDR original polytomous	.79 [.73, .85]	−.39**	−.21*
	Denial	.64 [.54, .75]	−.50**	−.26*
	Enhancement	.67 [.57, .77]	−.18	−.10*
	BIDR-D20	.83 [.79, .88]	−.30**	−.14
	Denial	.73 [.65, .81]	−.40**	−.22*
	Enhancement	.76 [.68, .83]	−.12	−.02

SDE, Self-Deceptive Enhancement; IM, Impression Management; BIDR-D20, 20 item short version that can only be used Dichotomous. Omega of BIDR-D20 was calculated with *n* = 99. *p ≤ .05, **p ≤ .001.

##### Aggression Questionnaire

To assess self-reported aggression and informant reports of aggression, an adapted version of the AQ (Buss and Perry, 1992; Dutch translation: Meesters et al., 1996) was administered. As informants could not answer all questions as they refer to non-observable behavior, only the 17 items that describe observable aggressive behavior were included to assess general levels of more explicit aggressive behavior (cf. Banse et al., 2014). Each item was answered on a 5-point Likert scale (1 = extremely like me, 5 = extremely unlike me). This 17-item AQ scale showed good internal consistency (*α* = .81). For the informant ratings, the internal consistency of the aggregate ratings was α = .91, which is similar to the Banse et al. (2014) study.

#### Analytic approach

Associations between study variables were examined using Spearman’s rho correlations because of non-normal distributions (e.g., Field, 2009). We conducted multiple hierarchical regression analyses to predict informant-reported aggression by self-reported aggression while controlling for IM and SDE. In all analyses, we included the main effects of age and self-reported aggression in the first step. In step two, we added all the main effects of SDE and IM. In step 3, two-way interactions between self-reported aggression and IM, and self-reported aggression and SDE were added to the model. All independent variables were mean-centered to reduce problems with multicollinearity (Kraemer and Blasey, 2004). Bootstrapping (*n* = 1,000) was used to compute confidence intervals and robust estimates of standard errors. Coefficients were deemed significant when zero was not included in the 95% confidence intervals. Analyses were conducted using R version 3.3.3 (R Core Team, 2017) package Lavaan (Version 6.3) (Rosseel, 2012).

### Results

Correlational analyses showed that self-reported aggression was highly correlated with informant-reported aggression (Table 6). For SDE, higher scores on the polytomously scored BIDR Denial subscales and the BIDR-D20 were associated with lower levels of self-reported aggression. Also, higher levels of IM on both the 40-item BIDR and short BIDR-D20 were correlated moderately with lower levels of self-reported aggression, except for the Enhancement subscales. This indicates that, overall, higher levels of IM and its deliberate denial of negative attributes were associated with lower levels of self-reported aggression. All significant correlations with self-reported aggression were small/medium to large (rs between −.23 and −.50).

Regarding informant-reported aggression, a higher score of the SDE Enhancement subscale of the BIDR-D20 was associated with higher scores of informant-reported aggression. For IM, significant negative associations were found, ranging from small to medium, except for the BIDR-D20 and the BIDR-D20 Enhancement subscale.

To investigate the effect of SDE and IM on the relationship between self-reported aggression and informant-reported aggression, moderation analyses were performed. Analyses (see Table 7, Model 1.1, and 2.1) indicated that there was no significant interaction between self-reported aggression and SDE and IM in predicting informant-reported aggression. In addition, the association between self-reported aggression and informant-reported aggression did not change when controlling for IM and SDE. The main effect of self-reported aggression indicated that higher levels of self-reported aggression were associated with higher levels of informant-reported aggression. No main effects for SDE or IM were found.

**Table 7:** Regression analyses for aggression, informant-reported aggression, SDE, IM, original BIDR and BIDR-D20 (*N* = 100).

Model	Peer-reported aggression														
	Estimate (SE)		95% CI												
Model 0															
R^2^	0.336														
Constant	2.05	(0.04)	1.96, 2.16												
Age	0.00	(0.00)	−0.01, 0.00												
Aggression	**0.55**	**(0.08)**	**0.35, 0.72**												

Model	Peer-reported aggression								
	BIDR original dichotomous			BIDR original polytomous			BIDR-D20		
	Estimate (SE)		95% CI	Estimate (SE)		95% CI	Estimate (SE)		95% CI
Model 1									
R^2^	0.34			0.34			0.34		
Constant	2.05	(0.04)	1.98, 2.12	2.05	(0.04)	1.98, 2.12	2.05	(0.04)	1.98, 2.12
Age	0.00	(0.00)	−0.01, 0.01	0.00	(0.00)	−0.01, 0.01	0.00	(0.00)	−0.01, 0.01
Aggression	**0.56**	**(0.09)**	**0.31, 0.79**	**0.58**	**(0.09)**	**0.29, 0.84**	**0.57**	**(0.08)**	**0.34, 0.79**
SDE	0.14	(0.25)	−0.49, 0.99	0.02	(0.07)	−0.16, 0.24	0.14	(0.21)	−0.45, 0.74
IM	0.03	(0.28)	−0.82, 0.82	0.04	(0.07)	−0.16, 0.23	0.06	(0.21)	−0.50, 0.69
Model 1.1									
R^2^	0.34			0.34			0.34		
Constant	2.05	(−0.05)	1.97, 2.13	2.04	(0.05)	1.98, 2.13	2.06	(0.04)	1.97, 2.12
Age	0.00	(0.00)	−0.01, 0.01	0.00	(0.00)	−0.01, 0.01	0.00	(0.00)	−0.01, 0.01
Aggression	**0.56**	**(−0.10)**	**0.20, 0.82**	**0.58**	**(0.10)**	**0.21, 0.86**	**0.58**	**(0.09)**	**0.29, 0.85**
SDE	0.14	(−0.27)	−0.70, 0.97	0.01	(0.07)	−0.19, 0.26	0.13	(0.22)	−0.45, 0.87
IM	0.03	(−0.31)	−0.96, 0.91	0.04	(0.07)	−0.18, 0.21	0.07	(0.23)	−0.63, 0.78
SDE*Agg.	−0.01	(−0.59)	−1.70, 1.87	−0.06	(0.16)	−0.50, 0.40	−0.12	(0.48)	−1.76, 1.34
IM*Agg.	0.03	(−0.053)	−1.49, 1.71	−0.04	(0.12)	−0.41, 0.31	0.10	(0.44)	−1.17, 1.54
Model 2									
R^2^	0.34			0.34			0.35		
Constant	2.05	(0.04)	1.97, 2.12	2.05	(0.04)	1.97, 2.12	2.05	(0.04)	1.97, 2.12
Age	0.00	(0.00)	−0.01, 0.01	0.00	(0.00)	−0.01, 0.01	0.00	(0.00)	−0.01, 0.01
Aggression	**0.55**	**(0.10)**	**0.28, 0.81**	**0.58**	**(0.10)**	**0.29, 0.90**	**0.55**	**(0.09)**	**0.32, 0.89**
SDE Denial	0.06	(0.23)	−0.61, 0.76	0.01	(0.06)	−0.17, 0.18	0.08	(0.18)	−0.45, 0.66
SDE Enhanc.	0.08	(0.24)	−0.62, 0.81	0.02	(0.07)	−0.16, 0.23	0.07	(0.18)	−0.49, 0.62
IM Denial	−0.07	(0.26)	−0.85, 0.73	0.02	(0.07)	−0.19, 0.20	−0.11	(0.20)	−0.74, 0.47
IM Enhanc.	0.10	(0.27)	−0.74, 0.91	0.02	(0.06)	−0.18, 0.19	0.15	(0.18)	−0.43, 0.67
Model 2.2									
R^2^	0.36			0.38			0.36		
Constant	2.04	(0.05)	1.82, 2.14	2.04	(0.05)	1.82, 2.14	2.04	(0.05)	1.82, 2.14
Age	0.00	(0.00)	−0.01, 0.01	0.00	(0.00)	−0.01, 0.01	0.00	(0.00)	−0.01, 0.01
Aggression	**0.52**	**(0.12)**	**0.09, 0.86**	**0.54**	**(0.12)**	**0.14, 0.88**	**0.55**	**(0.10)**	**0.26, 0.93**
SDE Denial	0.04	(0.26)	−0.68, 0.95	−0.04	(0.06)	−0.24, 0.17	0.05	(0.20)	−0.55, 0.75
SDE Enhanc.	0.09	(0.24)	−0.69, 0.80	0.04	(0.07)	−0.15, 0.27	0.04	(0.21)	−0.62, 0.68
IM Denial	−0.08	(0.31)	−1.14, 0.75	0.02	(0.08)	−0.22, 0.25	−0.10	(0.22)	−0.77, 0.62
IM Enhanc.	0.07	(0.32)	−0.85, 1.22	0.00	(0.07)	−0.19, 0.24	0.16	(0.22)	−0.50, 0.90
SDE Denial*Agg.	−0.55	(0.52)	−2.28, 0.88	−0.31	(0.15)	−0.82, 0.11	−0.25	(0.42)	−1.69, 0.87
SDE Enhanc.*Agg.	0.54	(0.57)	−1.16, 2.19	0.31	(0.18)	−0.20, 0.85	0.07	(0.40)	−1.16, 1.26
IM Denial*Agg.	0.05	(0.45)	−1.77, 1.28	0.12	(0.12)	−0.25, 0.51	−0.16	(0.38)	−1.48, 0.91
IM Enhanc.*Agg.	0.27	(0.69)	−1.64, 3.14	−0.09	(0.14)	−0.48, 0.41	0.31	(0.44)	−1.04, 1.88

IM, Impression Management; SDE, Self-Deceptive Enhancement; Agg., Aggression; Enhanc., Enhancement; BIDR-D20, 20 item short version that can only be used Dichotomous. CI, intervals were corrected for multiple testing. Significant values (*p* ≤ .05) are in bold.

### Discussion

The aim of study 3 was to investigate the utility of the 40-item BIDR and the BIDR-D20 short form as a validity scale by comparing self-reported aggressive behavior with informant reports of aggressive behavior. Results indicated that all versions correlated with self-reported aggression, but there was no evidence for SDE or IM as a response bias for which one should statistically control. One of the most interesting aspects of Study 3 was the strength of associations between IM and informant-reported aggression. If IM is a measure of intentional impression management, there is no reason why it should be strongly associated with informant-reported aggression scores in an anonymous research context. This seems to suggest that IM is serving as a personality measure, at least in a context without specific motivation for distortion. Since no clear motivation for socially desirable responding (SDR) was provided, consideration of both personality traits and response tendencies is required. Some scholars propose that SDR scales reflect a stable individual characteristic (e.g., Pauls and Stemmler, 2003; Smith and Ellingson, 2002; Uziel, 2010), while others suggest these measures capture both inherent traits and situational response patterns (see Connelly and Chang, 2016, for a meta-analysis). Historically, SDR has been regarded as a deliberate attempt to distort responses, particularly in forensic assessments. However, more recent findings indicate that SDR may function as a lasting personality trait, with lower levels being linked to higher recidivism rates (Mills and Kroner, 2005, 2006; Mills et al., 2003). This viewpoint is consistent with Uziel (2010) concept of impression management (IM) as a form of socially driven self-regulation. According to this perspective, individuals who score highly on IM are adept at modifying their behavior to meet social norms, especially in contexts where consequences or incentives are significant. This interpretation aligns with the results of the present study, where informant assessments highlight a negative correlation between IM and reported aggression.

## General discussion

The goal of the current study was to create and validate a short form version of the Dutch language version of the 40-item BIDR Version 6, taking its multidimensionality and scoring method into account. Specifically, we investigated (a) the BIDR item qualities using IRT for dichotomous and polytomous scoring methods in a multidimensional framework, (b) the performance of the short form compared to the 40-item BIDR, and (c) the ability of the BIDR SDE and IM scales to improve convergence between self- and other-ratings. Based on Studies 1 and 2, a short form version of the BIDR was developed, the BIDR-D20, containing 20 dichotomously scored items (10 IM, 10 SDE). Overall, the BIDR-D20 short form performed equal to or even outperformed the 40-item BIDR in terms of internal consistency, test–retest reliability, and nomological network. Compared to short forms from previous studies, the BIDR-D20 performed similarly with regard to the overlap in items (i.e., agreement between 31–81%). However, looking at the item overlap across short-forms, items 15 and 17 were also included in the BIDR-D20.

An important finding was that generally speaking the dichotomous method performed better than the polytomous scoring method (see also Asgeirsdottir et al., 2016). Although our intention was to create a short form that could be scored dichotomously as well as polytomously, ICCs showed that polytomous scoring included superfluous response options and, in some cases, a skewed answering distribution indicating a possible dichotomous answering pattern. Dichotomous scoring was found to fit better in terms of factor loadings and was more suitable for distinguishing between participants scoring high versus low on SDE/IM. This contrasts with studies that favor polytomous scoring (Stöber et al., 2002; Vispoel and Kim, 2014; Vispoel and Tao, 2013). However, the downside of using dichotomous scoring is that it ignores individuals with a tendency for desirable responding but who also avoid extreme answers, and the loss of information over the full trait of SDE or IM by singling out extreme responses on the higher end of the trait (e.g., Stöber et al., 2002). This could indicate that dichotomous scoring is better for flagging faking good, but misses faking bad (always completely denying the social option, indicating a more antisocial impression) and intermediate levels of social desirable responding. However, in an applied context “flagging faking good” is likely a more important goal for the BIDR than are missing fake bad approaches or identifying intermediate levels of faking good. Vispoel et al. (2019) proposed an alternative scoring method; only exaggerated denial of these behaviors would receive a score of 0 (Likert scale 1–2 answers receive 0), whereas all other responses would receive a score of 1. They argued that this new scoring system offered improved sensitivity at lower construct levels and provided more reliable cut-off scores for identifying faked-bad responses than the original scoring method. Additionally, as argued by Vispoel et al. (2023), using polytomous BIDR scores when measuring individual differences in SDE and IM does not dismiss the use of dichotomous scores for detecting faking.

The results of Study 3 indicated that, for both the 40-item BIDR and the short BIDR-D20, SDE and IM did not behave as response styles for which it is needed to statistically control. This is in line with previous studies indicating that controlling for SDR did not change or reduce the relationship between risk factors and recidivism (Mills and Kroner, 2006; Stevens et al., 2016) or the relationship between self-reported personality traits and informant-reported personality traits (e.g., Borkenau and Ostendorf, 1992; Pauls and Stemmler, 2003). Some studies have indicated that the scales assess some degree of bias, but the general consensus seems to be that the BIDR assesses substance in the form of a more enduring personality characteristic over responding style (e.g., Pauls and Stemmler, 2003; Schwartz et al., 1997).

However, as social desirability is viewed as a more personality characteristic, which is more manifested in some situations, social desirability could be more likely to affect self-reports with more sensitive topics (Johnson and Van de Vijver, 2003).

### Limitations

This study has several limitations. First, we only used general population samples. Although past research suggests that the BIDR’s reliability and validity are supported in both clinical and nonclinical populations, replication of the psychometric properties of the BIDR-D20 in clinical samples is recommended. This is relevant because in the current study participants did not gain anything by answering socially desirable. In forensic or correctional settings, for instance, there is often a secondary gain in portraying oneself in a favorable light (e.g., reduced sentence, child custody cases). In line with this, one could argue that the lack of provided motivation for social desirable answering is a limitation of Study 3. We recognize that providing a clear motivation to answer in a socially desirable manner could have increased the likelihood of a moderation effect.

Second, Study 2 focused exclusively on the association between self-report variables, raising concerns that shared reporter variance may inflate the magnitude of associations between the BIDR and Big Five personality dimensions and risk factors for deviant behavior. Partly to address the concerns about shared reporter variance, we used informant reports instead of merely self-reports in Study 3. However, though the sample size of this study was big enough to detect large effect sizes (*d* = .80), the power (1- *β*) to detect medium effect sizes was = .71, which means that the chance of making a type two error (b) was .29. Additionally, although the samples in Studies 1 and 2 were large and diverse, they are not representative of the Dutch population. While network sampling is a quick and cost-effective method (Etikan and Bala, 2017), this method has some weaknesses, namely its sampling is non-random and there is no ability to know how the study sample resembles the target population, leading to limited generalizability (Etikan and Bala, 2017). According to Stratton (2021), there are steps that can improve the credibility of this popular and simple method, for example: Recruit as many participants as possible and collect data in a diversified manner. To improve representativeness and participation, use different modes for gaining participants (online environments, e-mail/LinkedIn/Facebook/…). Although we took one or both of these steps in the current studies, the possibility of limited generalizability should be considered. Furthermore, study 3 included only male participants indicating that the results could not be generalized to the general population.

A third limitation is that in Study 2 and Study 3, participants completed the BIDR-40, from which the BIDR-DP12 and BIDR-D20 scores were derived. Since the item order in the BIDR-40 differs from the shorter versions, this may introduce a context effect that would not occur if, for example, the BIDR-D20 was administered independently. Context effects arise when earlier questions influence how respondents interpret or answer later ones, potentially affecting study results (e.g., Tourangeau et al., 2003). However, administering all three versions separately would have led to repeated exposure to certain items.

Lastly, though translation of the BIDR was done with care using backward and forward translations and consulting bilingual translators, translation of questionnaires do not always succeed in maintaining the intended meaning (Harkness et al., 2004). However, overall, results were in line with previous studies, which suggests that our translation seems adequate.

### Future research

The findings provide several leads for future research. First, more research is needed to explore differences between scoring option and its implications for research and clinical practice. In addition, Paulhus (1994) indicated that the BIDR could be administered using either a 7-point or a 5-point scale. However, currently there are no studies using the 5-point scale when comparing the polytomous and dichotomous scoring methods. Perhaps the 5-point scale would have produced different results (see also Stöber et al., 2002). In addition, future research could examine whether changing the response choices to Yes/No vs. scoring the extremes of the existing 7-point Likert scale would change the findings, given the benefit of dichotomous scoring in this line of research.

Moreover, further research could attempt to examine the effects of the cross-cultural phenomenon of the BIDR. That is, the current study assumes prima facie that the translation method employed was sufficient as due diligence was taken in the translation process. However, we did not examine language invariance of the Dutch translation of the BIDR (i.e., Dutch vs. English). The translation may be fine but culturally do the constructs being assessed mean the same thing (i.e., Netherlands vs. the U.S.)?

In addition, admitting to flaws and weaknesses about more general topics (e.g., gossiping, taking sick leave without being sick) does not automatically reflect the tendency to answer items that are more sensitive in nature, especially on an individual level (Keown et al., 2010). Therefore, a social desirability questionnaire or items related to the topic of self-reports may thus be more suitable to detect socially desirable responses. For example, including questions concerning common though socially sensitive sexual behavior in a self-report assessing pedophilic feelings.

## Conclusion and implications

Overall, our results indicate that the BIDR-D20 is a worthy replacement of the 40-item BIDR Version 6, with the same psychometric properties but being less-time-consuming. Although the overall results do not indicate that IM or SDE should be considered a response bias for which to statistically control, this does not mean that these measures should not be used. The use of the BIDR can provide valuable information, at least on self-presentation, socially approved relational skills and the need for social conformity (Mills et al., 2003).

## Data Availability

The raw data supporting the conclusions of this article will be made available by the authors, without undue reservation.
