# Supplementary material for: Examining a Dutch short form of The Balanced Inventory of Desirable Responding Version 6: comparing polytomous and dichotomous scoring methods in a multidimensional framework
Source: Front Psychol. 2025 Jun 13;16:1532969. doi: 10.3389/fpsyg.2025.1532969 (PMC12202607; doi:10.3389/fpsyg.2025.1532969)

Guide to the Online Supplementary Material

"Validation of a Dutch Short Form of the Balanced Inventory of Desirable Responding (BIDR-6): Comparing Polytomous and Dichotomous Scoring Methods in a Multidimensional Framework"

The Appendix is meant to supplement the material presented in the full paper. The goal of this information is to supply interested readers with more details surrounding the results presented in the full paper. Below the sections included in the Appendix are described.

2.1. Local dependence and item fit, estimator parameters, ICCs and IICs Study 1.

This file is supplementary to the main text in the full article and contains tables and figures regarding the local dependence, item fit, estimator parameters, ICCs and IICs of Study 1.

2.2. Omega coefficient, Test-Retest Correlations and Correlations between BIDR forms Study 1. Mean and standard deviations of the Original BIDR (Version 6) and short forms Studies 1-3.

This file is supplementary to the main text, and it contains the exact statistics regarding psychometric properties of the original BIDR (Version 6) and the short forms BIDR-DP12 and BIDR-D20. In addition it provides the reader with complementary information regarding the mean and standard deviations of the original BIDR (Version 6), and the BIDR-DP12 and BIDR-D20 of Studies 1-3.

2.3. IRT results of the BIDR-D20 Study 2.

This file contains the exact output for the IRT analyses (i.e. model fit, local dependence and item fit, estimator parameters, ICCs and IICs) of Study 2.

All files are meant to be helpful for properly interpreting the output, understand and evaluate the conclusions made in the full paper as well as making our results reproducible.

2.1. Local dependence and item fit, estimator parameters, ICCs and IICs Study 1.

This file is supplementary to the main text in the full article and contains tables and figures regarding the local dependence, item fit, estimator parameters, ICCs and IICs of Study 1.

Table S1

BIDR Item Labels

Nr	Items
1	My first impressions of people usually turn out to be right.
2	It would be hard for me to break any of my bad habits.(r)
3	I don't care to know what other people really think of me.
4	I have not always been honest with myself.(r)
5	I always know why I like things.
6	When my emotions are aroused, it biases my thinking.(r)
7	Once I've made up my mind, other people can seldom change my opinion.
8	I am not a safe driver when I exceed the speed limit.(r)
9	I am fully in control of my own fate.
10	It's hard for me to shut off a disturbing thought.(r)
11	I never regret my decisions.
12	I sometimes lose out on things because I can't make up my mind soon enough.(r)
13	The reason I vote is because my vote can make a difference.
14	My parents were not always fair when they punished me.(r)
15	I am a completely rational person.
16	I rarely appreciate criticism.(r)
17	I am very confident of my judgments
18	I have sometimes doubted my ability as a lover.(r)
19	It's all right with me if some people happen to dislike me.
20	I don't always know the reasons why I do the things I do.(r)
21	I sometimes tell lies if I have to.(r)
22	I never cover up my mistakes.
23	There have been occasions when I have taken advantage of someone.(r)
24	I never swear.
25	I sometimes try to get even rather than forgive and forget.(r)
26	I always obey laws, even if I'm unlikely to get caught.
27	I have said something bad about a friend behind his/her back.(r)
28	When I hear people talking privately, I avoid listening.
29	I have received too much change from a salesperson without telling him or her.(r
30	I always declare everything at customs.
31	When I was young I sometimes stole things.(r)
32	I have never dropped litter on the street.
33	I sometimes drive faster than the speed limit.(r)
34	I never read sexy books or magazines.
35	I have done things that I don't tell other people about.(r)
36	I never take things that don't belong to me.
37	I have taken sick-leave from work or school even though I wasn't really sick.(r)
38	I have never damaged a library book or store merchandise without reporting it.
39	I have some pretty awful habits.(r)
40	I don't gossip about other people's business.

Note. (r) indicates reversed coding.

Table S2

Local Independence Matrix for Dichotomous (Upper Triangle) and Polytomous (Lower Triangle) Scoring and Item Fit Statistics for SDE

																					S-X2 (df, *p*)	
Item	1	2	3	4	5	6	7	8	9	10	11	12	13	14	15	16	17	18	19	20	Dichotomous	Polytomous
1		0.15	-0.15	-0.02	-0.21	-0.06	-0.10	0.07	-0.20	-0.11	-0.17	0.02	-0.16	0.03	-0.15	-0.11	-0.06	-0.02	-0.18	0.08	08.957 (12, .819)	109.045 (102.000, .547)
2	-0.05		-0.03	-0.09	0.01	-0.12	-0.04	-0.01	-0.04	-0.07	-0.04	-0.10	-0.05	-0.16	-0.01	-0.22	0.03	-0.11	-0.07	-0.01	15.481 (12, .711)	190.683 (168.900, .547)
3	-0.18	0.11		-0.02	-0.26	-0.05	-0.17	-0.03	-0.18	0.03	-0.14	-0.01	-0.07	-0.01	-0.14	0.08	-0.20	0.02	-0.05	-0.02	11.905 (11, .711)	193.251 (171.100, .547)
4	0.02	-0.09	-0.10		0.01	-0.04	0.05	-0.04	-0.05	-0.07	0.01	0.05	-0.01	0.03	-0.05	-0.05	0.10	-0.19	0.02	0.00	11.168 (10, .711)	178.362 (162.300, .547)
5	0.02	0.02	-0.16	0.03		0.03	-0.11	-0.07	-0.09	0.06	-0.13	0.03	0.04	0.02	-0.09	-0.04	-0.17	0.03	-0.08	-0.06	10.077 (11, .750)	193.389 (153.200, .340)
6	0.00	-0.16	0.02	-0.08	-0.13		-0.13	-0.14	0.01	0.04	0.10	-0.07	0.10	-0.10	0.08	-0.18	0.04	-0.16	-0.02	-0.21	17.608 (11, .633)	175.903 (163.100, .547)
7	-0.01	0.01	-0.04	0.04	-0.05	0.00		0.08	-0.12	-0.07	-0.14	-0.01	-0.05	0.01	-0.14	-0.09	-0.01	0.03	-0.05	0.23	08.143 (12, .819)	185.733 (169.000, .547)
8	0.07	0.02	-0.05	-0.08	0.03	-0.05	0.00		0.08	-0.05	-0.13	-0.15	-0.02	-0.15	-0.03	-0.24	0.00	-0.18	0.02	0.07	10.072 (11, .750)	185.574 (180.100, .547)
9	-0.15	0.05	-0.10	-0.03	-0.01	-0.01	-0.08	-0.03		0.01	0.00	-0.12	-0.02	-0.01	-0.10	0.12	-0.15	-0.03	-0.05	-0.06	12.912 (11, .711)	173.841 (166.900, .547)
10	-0.05	-0.13	0.04	-0.28	-0.14	0.06	-0.09	-0.04	0.07		0.09	-0.06	-0.01	-0.05	0.03	-0.14	-0.01	-0.11	0.00	-0.22	08.332 (11, .819)	181.729 (165.900, .547)
11	-0.09	-0.02	0.01	0.05	-0.02	0.03	0.05	-0.05	-0.01	0.03		0.02	-0.01	-0.01	-0.11	0.13	-0.02	-0.09	-0.12	-0.11	11.517 (12, .750)	175.778 (172.100, .547)
12	-0.08	-0.09	0.00	-0.15	0.02	-0.11	0.09	-0.03	-0.02	-0.08	-0.01		0.05	-0.09	0.00	-0.13	0.03	-0.17	0.02	-0.09	09.421 (11, .777)	171.975 (172.700, .629)
13	0.03	-0.05	-0.12	-0.03	-0.02	0.06	-0.04	-0.07	-0.01	0.07	0.01	0.00		0.03	0.06	-0.06	0.00	-0.04	-0.01	0.04	21.355 (12, .630)	196.440 (191.100, .547)
14	-0.02	-0.08	-0.01	-0.01	0.01	-0.10	0.03	0.03	-0.01	-0.11	0.02	-0.04	-0.03		0.02	-0.13	-0.07	-0.07	-0.01	-0.16	18.982 (11, .630)	184.705 (156.000, .547)
15	-0.08	-0.03	-0.19	0.06	-0.13	0.10	-0.11	0.01	-0.13	0.07	-0.16	-0.10	-0.08	0.04		0.03	-0.06	-0.01	-0.13	-0.11	13.463 (11, .711)	156.606 (167.700, .756)
16	-0.05	0.05	-0.01	-0.10	0.03	-0.13	-0.11	-0.02	0.07	-0.02	-0.06	-0.07	0.10	0.02	-0.12		-0.12	-0.27	0.01	-0.07	15.863 (12, .711)	150.172 (158.200, .732)
17	0.01	-0.07	-0.42	-0.02	-0.22	-0.05	-0.13	0.07	-0.24	-0.07	-0.16	0.06	0.00	0.03	-0.12	-0.02		0.10	-0.06	-0.05	03.553 (10, .964)	112.881 (124.700, .764)
18	0.03	-0.07	0.03	-0.13	-0.04	-0.10	0.08	0.03	-0.01	-0.14	-0.01	-0.09	-0.12	-0.12	-0.03	-0.12	-0.01		-0.04	-0.15	11.680 (11, .711)	172.390 (165.700, .547)
19	-0.19	0.01	0.13	-0.01	-0.24	0.04	-0.09	-0.04	-0.12	0.13	-0.19	0.04	-0.01	-0.08	-0.14	0.15	-0.14	0.05		0.09	15.359 (12, .711)	179.046 (185.900, .732)
20	0.10	-0.14	-0.03	-0.24	0.13	-0.25	-0.03	-0.06	-0.02	-0.27	-0.03	-0.15	0.05	-0.12	-0.04	-0.01	0.10	-0.13	-0.18		06.416 (10, .819)	170.205 (162.600, .547)

Note. As an indication of local dependence, a cut-off of .30 minus the average correlation was used as a critical value for $Q3$ (for more information see Christensen et al., 2017). p-values were adjusted for false discovery rates (FDR; Benjamini & Hochberg, 1995). Significant values (p <.05) are in **Bold.** Item labels can be found in Table S1.

Table S3

Local Independence Matrix for Dichotomous (Upper Triangle) and Polytomous (Lower Triangle) Scoring and Item Fit Statistics for IM

																					S-X2 (df, *p*)	
Item	21	22	23	24	25	26	27	28	29	30	31	32	33	34	35	36	37	38	39	40	Dichotomous	Polytomous
21		0.15	-0.15	-0.02	-0.21	-0.06	-0.10	0.07	-0.20	-0.11	-0.17	0.02	-0.16	0.03	-0.15	-0.11	-0.06	-0.02	-0.18	0.08	06.643 (10, .842)	184.544 (186.400, .525)
22	0.19		-0.03	-0.09	0.01	-0.12	-0.04	-0.01	-0.04	-0.07	-0.04	-0.10	-0.05	-0.16	-0.01	-0.22	0.03	-0.11	-0.07	-0.01	14.007 (11, .628)	186.241 (182.900, .420)
23	-0.05	0.00		-0.02	-0.26	-0.05	-0.17	-0.03	-0.18	0.03	-0.14	-0.01	-0.07	-0.01	-0.14	0.08	-0.20	0.02	-0.05	-0.02	04.068 (09, .906)	166.426 (157.000, .290)
24	0.01	-0.09	-0.07		0.01	-0.04	0.05	-0.04	-0.05	-0.07	0.01	0.05	-0.01	0.03	-0.05	-0.05	0.10	-0.19	0.02	0.00	17.451 (12, .532)	103.398 (131.100, .963)
25	-0.17	0.02	0.07	-0.08		0.03	-0.11	-0.07	-0.09	0.06	-0.13	0.03	0.04	0.02	-0.09	-0.04	-0.17	0.03	-0.08	-0.06	13.078 (11, .633)	164.056 (185.400, .867)
26	-0.04	-0.18	-0.02	-0.02	-0.08		-0.13	-0.14	0.01	0.04	0.10	-0.07	0.10	-0.10	0.08	-0.18	0.04	-0.16	-0.02	-0.21	10.645 (12, .700)	202.793 (201.800, .467)
27	-0.07	-0.03	-0.11	0.00	-0.07	-0.16		0.08	-0.12	-0.07	-0.14	-0.01	-0.05	0.01	-0.14	-0.09	-0.01	0.03	-0.05	0.23	10.054 (11, .700)	210.641 (203.300, .348)
28	0.10	0.00	-0.03	-0.12	0.00	-0.22	0.11		0.08	-0.05	-0.13	-0.15	-0.02	-0.15	-0.03	-0.24	0.00	-0.18	0.02	0.07	06.717 (11, .864)	225.737 (204.700, .152)
29	-0.22	-0.04	-0.14	-0.02	-0.04	-0.01	-0.09	0.06		0.01	0.00	-0.12	-0.02	-0.01	-0.10	0.12	-0.15	-0.03	-0.05	-0.06	07.718 (11, .842)	170.235 (183.700, .753)
30	-0.10	-0.15	0.00	-0.08	0.10	0.11	-0.10	-0.12	0.00		0.09	-0.06	-0.01	-0.05	0.03	-0.14	-0.01	-0.11	0.00	-0.22	10.816 (12, .700)	197.825 (183.600, .229)
31	-0.24	-0.05	-0.07	-0.05	-0.07	0.12	-0.12	-0.08	0.04	0.09		0.02	-0.01	-0.01	-0.11	0.13	-0.02	-0.09	-0.12	-0.11	10.534 (11, .700)	**235.909 (173.700, .001)**
32	-0.11	-0.17	-0.02	-0.04	0.00	-0.10	-0.02	-0.20	-0.07	-0.11	0.00		0.05	-0.09	0.00	-0.13	0.03	-0.17	0.02	-0.09	12.195 (11, .633)	139.314 (132.100, .343)
33	-0.24	-0.16	-0.10	-0.02	-0.03	0.14	-0.03	-0.05	0.01	-0.01	0.00	0.17		0.03	0.06	-0.06	0.00	-0.04	-0.01	0.04	13.767 (11, .633)	159.980 (170.100, .696)
34	0.07	-0.14	0.05	-0.01	0.04	-0.10	-0.04	-0.21	-0.03	0.00	-0.03	-0.10	0.00		0.02	-0.13	-0.07	-0.07	-0.01	-0.16	14.870 (12, .628)	181.000 (200.800, .836)
35	-0.18	0.00	-0.05	0.00	-0.09	0.05	-0.20	-0.07	-0.15	0.01	-0.12	0.03	-0.02	0.06		0.03	-0.06	-0.01	-0.13	-0.11	14.530 (11, .628)	160.625 (177.900, .816)
36	-0.05	-0.02	0.13	-0.26	0.06	-0.14	-0.01	-0.22	0.07	0.02	0.15	-0.14	-0.11	-0.02	-0.02		-0.12	-0.27	0.01	-0.07	15.265 (10, .532)	**231.704 (177.000, .006)**
37	-0.09	-0.02	-0.21	0.07	-0.15	0.01	-0.10	0.02	-0.14	-0.01	-0.03	0.11	0.00	-0.08	-0.10	-0.12		0.10	-0.06	-0.05	21.315 (12, .267)	195.914 (183.600, .257)
38	-0.05	-0.01	0.06	-0.26	0.06	-0.18	0.01	-0.13	0.03	-0.06	-0.04	-0.14	-0.10	0.07	0.04	0.12	0.01		-0.04	-0.15	23.007 (10, .110)	176.034 (162.800, .234)
39	-0.12	-0.01	-0.14	0.12	-0.11	-0.01	-0.01	-0.02	-0.05	0.03	-0.13	0.01	-0.02	-0.02	-0.12	-0.04	-0.04	-0.02		0.09	24.557 (11, .110)	**241.613 (196.700, .017)**
40	0.02	-0.01	-0.01	-0.07	0.05	-0.29	0.32	0.14	0.00	-0.16	-0.16	-0.13	0.01	-0.16	-0.12	-0.04	-0.03	-0.06	0.04		11.784 (11, .633)	229.133 (195.200, .054)

Note. As an indication of local dependence, a cut-off of .30 minus the average correlation was used as a critical value for *Q* 3 (for more information see Christensen et al., 2017). *p* -values were adjusted for false discovery rates (FDR; Benjamini & Hochberg, 1995). Significant values ($p < .05$) are in **Bold.** Item labels can be found in Table S1.

Table S4

Local Independence Matrix for Polytomous Scoring and Item Fit Statistics for SDE Without Item Pair 3-17

Item	1	2	4	5	6	7	8	9	10	11	12	13	14	15	16	18	19	S-X2 (df, *p*)
1																		100.728 (104.90, .888)
2	-0.05																	164.925 (159.10, .888)
4	0.00	-0.09																166.502 (157.50, .888)
5	-0.15	0.06	0.00															171.367 (141.00, .846)
6	0.01	-0.15	-0.08	-0.14														165.224 (147.10, .888)
7	-0.02	0.00	0.02	-0.16	0.00													136.488 (159.20, .943)
8	0.07	0.02	-0.08	0.03	-0.05	0.00												140.660 (169.70, .943)
9	-0.23	0.06	-0.05	-0.35	0.01	-0.11	-0.04											161.869 (159.90, .888)
10	-0.04	-0.12	-0.28	-0.17	0.07	-0.08	-0.04	0.09										142.389 (146.70, .888)
11	-0.13	-0.02	0.04	-0.22	0.04	0.04	-0.05	-0.08	0.04									155.524 (164.00, .888)
12	-0.07	-0.08	-0.15	0.04	-0.11	0.10	-0.03	-0.01	-0.07	0.00								151.356 (160.90, .888)
13	0.00	-0.05	-0.04	-0.12	0.07	-0.05	-0.08	-0.05	0.07	-0.02	0.00							143.468 (146.60, .888)
14	-0.02	-0.08	-0.01	0.03	-0.10	0.02	0.03	-0.01	-0.11	0.03	-0.04	-0.03						119.332 (133.50, .892)
15	-0.08	-0.04	0.03	-0.36	0.10	-0.10	0.01	-0.18	0.09	-0.18	-0.08	-0.10	0.03					160.342 (161.90, .888)
16	-0.05	0.05	-0.11	0.03	-0.13	-0.11	-0.03	0.06	-0.02	-0.07	-0.07	0.10	0.02	-0.13				164.522 (153.50, .888)
18	0.04	-0.07	-0.13	-0.04	-0.09	0.09	0.03	0.01	-0.14	0.00	-0.09	-0.12	-0.12	0.00	-0.12			131.422 (116.90, .888)
19	-0.11	-0.02	-0.03	-0.23	0.03	-0.02	-0.04	-0.02	0.13	-0.11	0.05	0.02	-0.10	0.02	0.13	0.07		161.073 (164.90, .888)
20	0.10	-0.14	-0.25	0.13	-0.25	-0.04	-0.06	-0.04	-0.27	-0.05	-0.16	0.04	-0.12	-0.05	-0.01	-0.13	-0.16	139.110 (153.70, .892)

Note. As an indication of local dependence, a cut-off of .30 minus the average correlation was used as a critical value for $Q3$ (for more information see Christensen et al., 2017). p-values were adjusted for false discovery rates (FDR; Benjamini & Hochberg, 1995). Significant values ($p \leq .05$) are in **Bold**. Item labels can be found in Table S1.

Table S5

Factor Loadings, Discrimination, Threshold, and Difficulty Parameters for SDE for Dichotomous and Polytomous Scoring

	Dichotomous scoring												Polytomous scoring											
																				Threshold 1				
Item	Factor		*a1*	97.5% CI		*a2*	97.5% CI		*d*	97.5% CI *d*		*b*	Factor		*a1*	97.5% CI		*a2*	97.5% CI		*d*	97.5% CI *d*		*b*
nr.	1	2		*2.5*	*97.5*		*2.5*	*97.5*		*2.5*	*97.5*		1	2		*2.5*	*97.5*		*2.5*	*97.5*		*2.5*	*97.5*	
1	.32		0.57	0.32	0.81				-0.72	-0.96	-0.58	1.28	.33		0.60	0.38	0.83				4.73	3.92	5.55	-7.87
3	.63		1.39	0.92	1.86				-2.48	-2.93	-2.03	1.78												
5	.60		1.27	0.91	1.63				-0.43	-0.65	-0.21	0.34	.60		1.27	0.94	1.61				5.31	4.42	6.21	-4.17
7	.33		0.59	0.31	0.85				-1.09	-1.29	-0.88	1.83	.22		0.38	0.16	0.60				3.62	3.12	4.13	-9.53
9	.61		1.31	0.92	1.70				1.57	-1.88	-2.16	-1.20	.47		0.91	0.65	1.17				2.89	2.53	3.26	-3.17
11	.58		1.22	0.76	1.67				-2.69	-3.14	-2.23	2.21	.36		0.66	0.44	0.89				2.22	1.93	2.50	-3.34
13	.32		0.58	0.34	0.83				-0.83	-1.03	-0.64	1.42	.16		0.28	0.08	0.48				1.90	1.66	2.15	-6.75
15	.62		1.33	0.92	1.74				-1.87	-2.22	-1.53	1.41	.44		0.83	0.58	1.08				2.45	2.13	2.76	-2.96
17	.74		1.86	1.31	2.42				-1.77	-2.17	-1.36	0.95												
19	.40		0.75	0.48	1.02				-0.81	-1.01	-0.61	1.08	.23		0.40	0.18	0.61				2.78	2.43	3.12	-7.02
2		.51				1.01	0.69	1.33	-1.36	-1.62	-1.11	1.35		.44				0.82	0.62	1.03	3.79	3.29	4.30	-4.61
4		.63				1.51	1.08	1.93	-1.34	-1.64	-1.04	0.89		.59				1.25	1.00	1.50	3.52	3.08	3.97	-2.82
6		.59				1.23	0.80	1.66	-2.42	-2.83	-2.01	1.97		.48				0.94	0.71	1.16	2.56	2.24	2.88	-2.74
8		.35				0.63	0.39	0.87	-0.14	-0.32	0.04	0.22		.17				0.30	0.12	0.48	2.60	2.28	2.92	-8.71
10		.59				1.24	0.84	1.64	-2.02	-2.37	-1.67	1.62		.56				1.15	0.90	1.39	2.88	2.52	3.24	-2.51
12		.53				1.07	0.76	1.39	-0.85	-1.08	-0.63	0.79		.47				0.89	0.68	1.11	3.96	3.42	4.50	-4.43
14		.43				0.80	0.54	1.06	0.22	0.03	0.40	-0.27		.36				0.67	0.46	0.87	3.32	2.90	3.75	-4.99
16		.31				0.56	0.33	0.79	-0.43	-0.61	-0.25	0.78		.23				0.40	0.21	0.59	5.73	4.34	7.12	-14.28
18		.54				1.08	0.76	1.40	-0.87	-1.10	-0.64	0.80		.48				0.92	0.70	1.14	3.54	3.09	3.99	-3.85
20		.75				1.91	1.35	2.46	-1.76	-2.17	-1.36	0.92		.65				1.44	1.17	1.72	3.76	3.28	4.24	-2.61

Note. *a* is the discrimination parameter (or slope). For polytomous scoring, given that each item has 7 item categories, there are 6 thresholds (i.e., difficulty parameter, *b*) creating these 7 categories. The mirt package in R only provides estimates for intercepts which can be transformed into threshold values for each item using the following formula (-*d* /*a*), where *d* is the intercept value for the corresponding response category and *a* is the slope for the item. Darker notation indicate greater factor loadings (< .40; .40 - .55; ≥.55) and discrimination parameters (< .65; .65 - 1.34; >1.34). Factor 1 is Enhancement, Factor 2 Denial. Item labels can be found in Table S1.

Table S5 Continued

	Polytomous scoring																			
	Threshold																			
	2				3				4				5				6			
Item	*d*	97.5% CI		*b*	*d*	97.5% CI *d*		*b*	*d*	97.5% CI *d*		*b*	*d*	97.5% CI *d*		*b*	*d*	97.5% CI *d*		*b*
nr.		*2.5*	*97.5*			*2.5*	*97.5*			*2.5*	*97.5*			*2.5*	*97.5*			*2.5*	*97.5*	
1	3.86	3.32	4.41	-6.43	2.75	2.41	3.09	-4.57	0.93	0.74	1.13	-1.55	-0.77	-0.96	-0.58	1.28	-2.88	-3.25	-2.52	4.80
3																				
5	3.33	2.86	3.79	-2.61	2.11	1.77	2.44	-1.65	0.90	0.66	1.14	-0.71	-0.43	-0.65	-0.21	0.34	-2.54	-2.91	-2.17	2.00
7	2.01	1.76	2.27	-5.30	0.89	0.70	1.07	-2.34	-0.06	-0.23	0.11	0.15	-1.04	-1.23	-0.85	2.73	-2.60	-2.93	-2.28	6.85
9	2.06	1.78	2.35	-2.26	1.18	0.95	1.40	-1.29	-0.25	-0.45	-0.06	0.28	-1.37	-1.60	-1.13	1.50	-2.85	-3.22	-2.48	3.13
11	0.65	0.46	0.84	-0.98	-0.43	-0.62	-0.25	0.65	-1.53	-1.76	-1.30	2.30	-2.32	-2.62	-2.03	3.50	-4.15	-4.77	-3.54	6.26
13	1.38	1.17	1.58	-4.88	0.87	0.69	1.05	-3.08	0.07	-0.10	0.23	-0.23	-0.78	-0.96	-0.60	2.75	-2.03	-2.29	-1.78	7.21
15	1.52	1.28	1.76	-1.84	0.72	0.52	0.92	-0.87	-0.54	-0.73	-0.34	0.65	-1.58	-1.83	-1.34	1.92	-3.22	-3.64	-2.81	3.90
17																				
19	1.60	1.38	1.83	-4.04	0.91	0.72	1.09	-2.29	0.07	-0.10	0.24	-0.17	-0.74	-0.92	-0.56	1.86	-2.02	-2.28	-1.76	5.09
2	1.98	1.72	2.24	-2.40	0.65	0.46	0.85	-0.79	-0.40	-0.59	-0.21	0.49	-1.27	-1.49	-1.06	1.55	-2.50	-2.82	-2.19	3.05
4	2.03	1.74	2.32	-1.62	0.63	0.41	0.84	-0.50	-0.43	-0.64	-0.21	0.34	-1.15	-1.39	-0.92	0.92	-2.87	-3.24	-2.50	2.30
6	0.89	0.68	1.10	-0.95	-0.27	-0.47	-0.08	0.29	-1.46	-1.70	-1.23	1.56	-2.19	-2.48	-1.90	2.34	-3.51	-3.96	-3.06	3.75
8	1.82	1.58	2.06	-6.11	1.18	0.99	1.38	-3.97	0.51	0.34	0.68	-1.70	-0.12	-0.29	0.05	0.40	-1.23	-1.43	-1.04	4.14
10	1.20	0.97	1.44	-1.05	-0.15	-0.35	0.06	0.13	-1.15	-1.38	-0.92	1.00	-1.91	-2.19	-1.64	1.67	-3.71	-4.19	-3.23	3.24
12	2.12	1.84	2.39	-2.37	0.92	0.72	1.13	-1.03	0.12	-0.07	0.31	-0.14	-0.78	-0.98	-0.58	0.87	-2.35	-2.65	-2.05	2.63
14	2.44	2.14	2.74	-3.67	1.63	1.40	1.86	-2.45	0.77	0.58	0.96	-1.16	0.22	0.04	0.40	-0.33	-0.92	-1.11	-0.72	1.37
16	2.79	2.45	3.14	-6.97	1.67	1.44	1.90	-4.16	0.75	0.57	0.94	-1.88	-0.42	-0.59	-0.24	1.03	-2.14	-2.40	-1.87	5.33
18	1.82	1.56	2.07	-1.98	0.76	0.56	0.97	-0.83	-0.16	-0.36	0.03	0.18	-0.82	-1.02	-0.62	0.89	-2.02	-2.29	-1.75	2.19
20	2.01	1.71	2.31	-1.39	0.68	0.45	0.91	-0.47	-0.56	-0.79	-0.33	0.39	-1.47	-1.73	-1.20	1.02	-3.16	-3.58	-2.75	2.19

Note . a is the discrimination parameter (or slope). For polytomous scoring, given that each item has 7 item categories, there are 6 thresholds (i.e., difficulty parameter, *b*) creating these 7 categories. The mirt package in R only provides estimates for intercepts which can be transformed into threshold values for each item using the following formula (-*d* /*a*), where *d* is the intercept value for the corresponding response category and *a* is the slope for the item. Factor 1 is Enhancement, Factor 2 Denial. Darker notation indicate greater factor loadings (< .40; .40 - .55; ≥.55) and discrimination parameters (< .65; .65 - 1.34; >1.34). Item labels can be found in Table S1.

Table S6

Factor Loadings, Discrimination, Threshold, and Difficulty Parameters for IM for Dichotomous and Polytomous Scoring

	Dichotomous scoring												Polytomous scoring											
																					Threshold 1			
Item	Factor Loading		*a1*	97.5% CI *a1*		*a2*	97.5% CI *a2*		*d*	97.5% CI *d*		*b*	Factor Loading		*a1*	97.5% CI *a1*		*a2*	97.5% CI *a2*		*d*	97.5% CI *d*		*b*
nr.	1	2		*2.5*	*97.5*		*2.5*	*97.5*		*2.5*	*97.5*		1	2		*2.5*	*97.5*		*2.5*	*97.5*		*2.5*	*97.5*	
21		.69				1.61	1.62	2.06	-1.51	-1.84	-1.18	0.94		.64				1.41	1.15	1.66	3.24	2.83	3.65	-2.30
23		.69				1.61	1.17	2.05	0.78	0.52	1.04	0.46		.53				1.06	0.83	1.29	4.40	3.77	5.02	-4.16
25		.60				1.27	0.93	1.61	0.15	-0.07	0.36	-0.11		.49				0.94	0.73	1.15	4.29	3.69	4.90	-4.55
27		.52				1.05	0.72	1.38	-1.59	-1.87	-1.31	-1.50		.47				0.91	0.70	1.11	2.12	1.84	2.39	-2.34
29		.51				1.07	0.75	1.38	-1.19	-1.44	-0.95	1.10		.50				0.99	0.77	1.21	1.03	0.82	1.25	-1.05
**31**		.44				0.83	0.57	1.09	-0.14	-0.32	0.05	-0.16		.47				0.91	0.69	1.13	1.48	1.25	1.71	-1.63
33		.21				0.37	0.06	0.68	-2.00	2.27	-1.74	5.41		.32				0.57	0.37	0.76	0.30	0.12	0.48	-0.53
35		.53				1.05	0.73	1.37	-1.38	-1.64	-1.12	-1.29		.58				1.21	0.97	1.45	1.50	1.24	1.75	-1.24
37		.43				0.81	0.54	1.08	-0.89	-1.10	-0.63	1.09		.52				1.05	0.81	1.28	0.90	0.69	1.11	-0.86
**39**		.47				0.90	0.59	1.21	-1.66	-1.93	-1.39	-1.81		.45				0.87	0.67	1.07	1.96	1.70	2.22	-2.27
22	.51		1.01	0.64	1.38				-2.05	-2.38	-1.72	2.03	.34		0.62	0.42	0.81				2.87	2.52	3.23	-4.66
24	.35		0.63	0.23	1.04				-2.66	-3.03	-2.28	4.22	.50		0.99	0.73	1.24				-0.18	-0.38	0.02	0.18
26	.46		0.88	0.59	1.17				-0.92	-1.14	-0.70	1.05	.49		0.95	0.73	1.18				2.60	2.28	2.93	-2.74
28	.52		1.03	0.67	1.39				-1.77	-2.07	-1.47	1.72	.47		0.90	0.68	1.12				2.23	1.94	2.51	-2.48
30	.31		0.55	0.32	0.79				-0.02	-0.20	0.15	0.04	.26		0.46	0.27	0.65				1.90	1.65	2.15	-4.15
32	.52		1.03	0.65	1.40				-0.21	-2.39	-1.72	0.20	.53		1.06	0.80	1.31				0.32	0.12	0.52	-0.30
34	.40		0.74	0.48	1.00				-0.56	-0.75	-0.37	0.76	.35		0.63	0.43	0.83				1.33	1.11	1.54	-2.09
**36**	.63		1.39	0.99	1.79				0.34	0.11	0.56	-0.24	.45		0.86	0.63	1.09				2.72	2.38	3.06	-3.16
38	.60		1.27	0.90	1..63				0.42	0.21	0.65	-0.33	.40		0.75	0.53	0.97				1.98	1.72	2.25	-2.64
40	.44		0.83	0.51	1.15				-1.60	-1.86	-1.34	1.93	.38		0.70	0.50	0.91				2.47	2.16	2.77	-3.52

Note. *a* is the discrimination parameter (or slope). For polytomous scoring, given that each item has 7 item categories, there are 6 thresholds (i.e., difficulty parameter, *b*) creating these 7 categories. The mirt package in R only provides estimates for intercepts which can be transformed into threshold values for each item using the following formula (-*d*/*a*), where *d* is the intercept value for the corresponding response category and *a* is the slope for the item. Factor 1 is Enhancement, Factor 2 Denial. Darker notation indicate greater factor loadings (< .40; .40 - .55; ≥.55) and discrimination parameters (< .65; .65 - 1.34; >1.34). Items **31, 36, 39** where indicated as misfitting (sign. S-X2 statistics) for polytomous scoring. Item labels can be found in Table S1.

Table S6 Continued

	Polytomous scoring																			
	Threshold																			
	2				3				4				5				6			
Item	*d*	97.5% CI *d*		*b*	*d*	97.5% CI *d*		*b*	*d*	97.5% CI *d*		*b*	*d*	97.5% CI *d*		*b*	*d*	97.5% CI *d*		*b*
nr.		*2.5*	*97.5*			*2.5*	*97.5*			*2.5*	*97.5*			*2.5*	*97.5*			*2.5*	*97.5*	
21	1.73	1.45	2.00	-1.23	0.40	0.17	0.62	-0.28	-0.65	-0.87	-0.42	0.46	-1.40	-1.65	-1.14	0.99	-3.15	-3.54	-2.75	2.24
23	3.36	2.93	3.78	-3.17	2.14	1.86	2.43	-2.03	1.26	1.03	1.49	-1.19	0.60	0.39	0.80	-0.56	-0.91	-1.12	-0.69	0.86
25	2.64	2.32	2.97	-2.80	1.64	1.40	1.88	-1.74	0.76	0.56	0.96	-0.81	0.11	-0.08	0.30	-0.12	-1.18	-1.40	-0.96	1.25
27	1.02	0.81	1.23	-1.13	-0.06	-0.25	0.13	0.06	-0.95	-1.15	-0.74	1.04	-1.52	-1.75	-1.29	1.68	-2.78	-3.12	-2.44	3.07
29	0.22	0.03	0.42	-0.22	-0.47	-0.67	-0.28	0.48	-0.89	-1.09	-0.68	0.90	-1.16	-1.38	-0.94	1.18	-1.74	-1.99	-1.49	1.77
**31**	0.82	0.62	1.03	-0.91	0.38	0.19	0.57	-0.42	0.04	-0.15	0.23	-0.05	-0.19	-0.38	0.00	0.21	-0.79	-0.99	-0.59	0.87
33	-0.46	-0.64	-0.28	0.81	-1.21	-1.41	-1.00	2.12	-1.72	-1.95	-1.48	3.02	-2.07	-2.33	-1.81	3.64	-2.54	-2.85	-2.23	4.47
35	0.30	0.09	0.51	-0.25	-0.38	-0.58	-0.17	0.31	-1.03	-1.25	-0.80	0.85	-1.44	-1.68	-1.19	1.19	-2.30	-2.60	-2.00	1.91
37	0.11	-0.09	0.30	-0.10	-0.37	-0.57	-0.17	0.36	-0.79	-1.00	-0.58	0.75	-0.98	-1.20	-0.76	0.94	-1.53	-1.77	-1.28	1.46
**39**	0.87	0.67	1.07	-1.00	-0.12	-0.30	0.07	0.13	-1.04	-1.24	-0.83	1.20	-1.62	-1.86	-1.39	1.87	-3.00	-3.37	-2.64	3.47
22	1.16	0.96	1.37	-1.89	-0.04	-0.21	0.14	0.06	-1.06	-1.25	-0.86	1.72	-1.84	-2.08	-1.59	2.98	-3.18	-3.58	-2.78	5.16
24	-1.10	-1.32	-0.87	1.11	-1.71	-1.97	-1.46	1.74	-2.28	-2.59	-1.98	2.32	-2.85	-3.20	-2.49	2.89	-4.36	-5.00	-3.73	4.43
26	1.22	1.00	1.44	-1.28	0.37	0.18	0.57	-0.39	-0.23	-0.42	-0.04	0.24	-0.92	-1.13	-0.71	0.97	-2.59	-2.92	-2.27	2.73
28	0.69	0.50	0.89	-0.77	-0.14	-0.33	0.05	0.16	-1.03	-1.24	-0.82	1.15	-1.68	-1.92	-1.43	1.87	-2.91	-3.27	-2.55	3.24
30	1.31	1.11	1.52	-2.86	0.98	0.79	1.17	-2.13	0.37	0.20	0.55	-0.82	-0.04	-0.22	0.13	0.10	-0.68	-0.86	-0.50	1.48
32	-0.59	-0.80	-0.38	0.56	-1.15	-1.38	-0.93	1.09	-1.62	-1.87	-1.37	1.54	-2.04	-2.32	-1.76	1.94	-3.13	-3.52	-2.73	2.96
34	0.58	0.39	0.76	-0.91	0.16	-0.02	0.34	-0.25	-0.35	-0.53	-0.17	0.55	-0.56	-0.74	-0.37	0.88	-1.29	-1.51	-1.08	2.04
**36**	1.87	1.61	2.12	-2.17	1.22	1.01	1.44	-1.42	0.66	0.47	0.86	-0.77	0.29	0.10	0.48	-0.34	-0.66	-0.86	-0.47	0.77
38	1.48	1.25	1.71	-1.97	1.07	0.87	1.28	-1.43	0.80	0.60	0.99	-1.06	0.36	0.17	0.54	-0.48	-0.36	-0.54	-0.17	0.47
40	0.94	0.75	1.14	-1.35	-0.02	-0.20	0.17	0.02	-0.94	-1.14	-0.75	1.35	-1.57	-1.80	-1.34	2.24	-3.24	-3.65	-2.83	4.62

Note. *a* is the discrimination parameter (or slope). For polytomous scoring, given that each item has 7 item categories, there are 6 thresholds (i.e., difficulty parameter, *b*) creating these 7 categories. The mirt package in R only provides estimates for intercepts which can be transformed into threshold values for each item using the following formula ($-d/a$), where *d* is the intercept value for the corresponding response category and *a* is the slope for the item. Factor 1 is Enhancement, Factor 2 Denial. Darker notation indicate greater factor loadings (< .40; .40 - .55; ≥.55) and discrimination parameters (< .65; .65 - 1.34; >1.34). Items **31, 36, 39** where indicated as misfitting (sign. S-X2 statistics) for polytomous scoring. Item labels can be found in Table S1.

Figure S1

Item Characteristic Curve (ICC) and Item Information Curve (IIC) for SDE Items Dichotomous (Left Rows) and Polytomous Scoring (Right Rows) of the BIDR 40-item Original Version

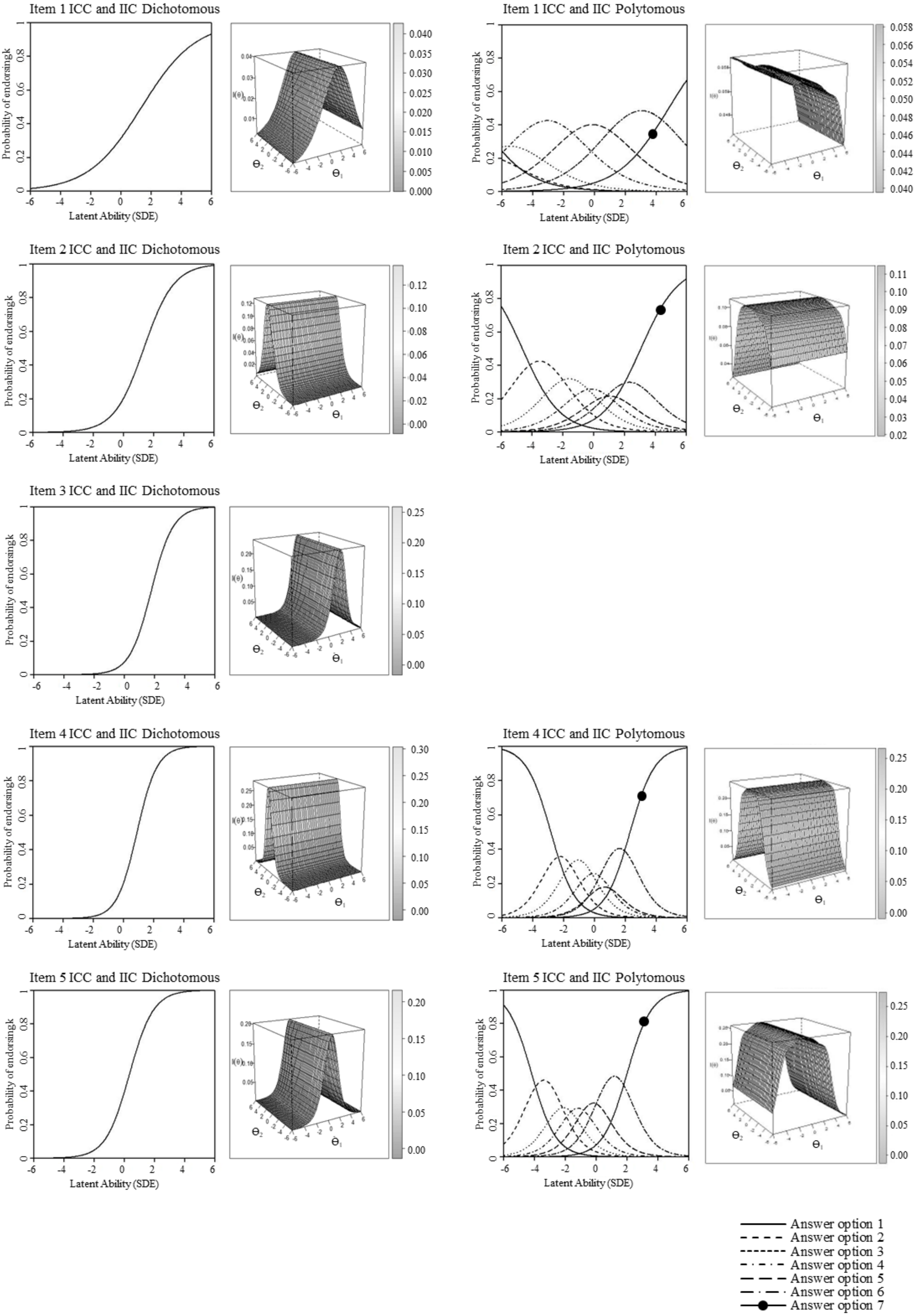

Figure S1 Continued

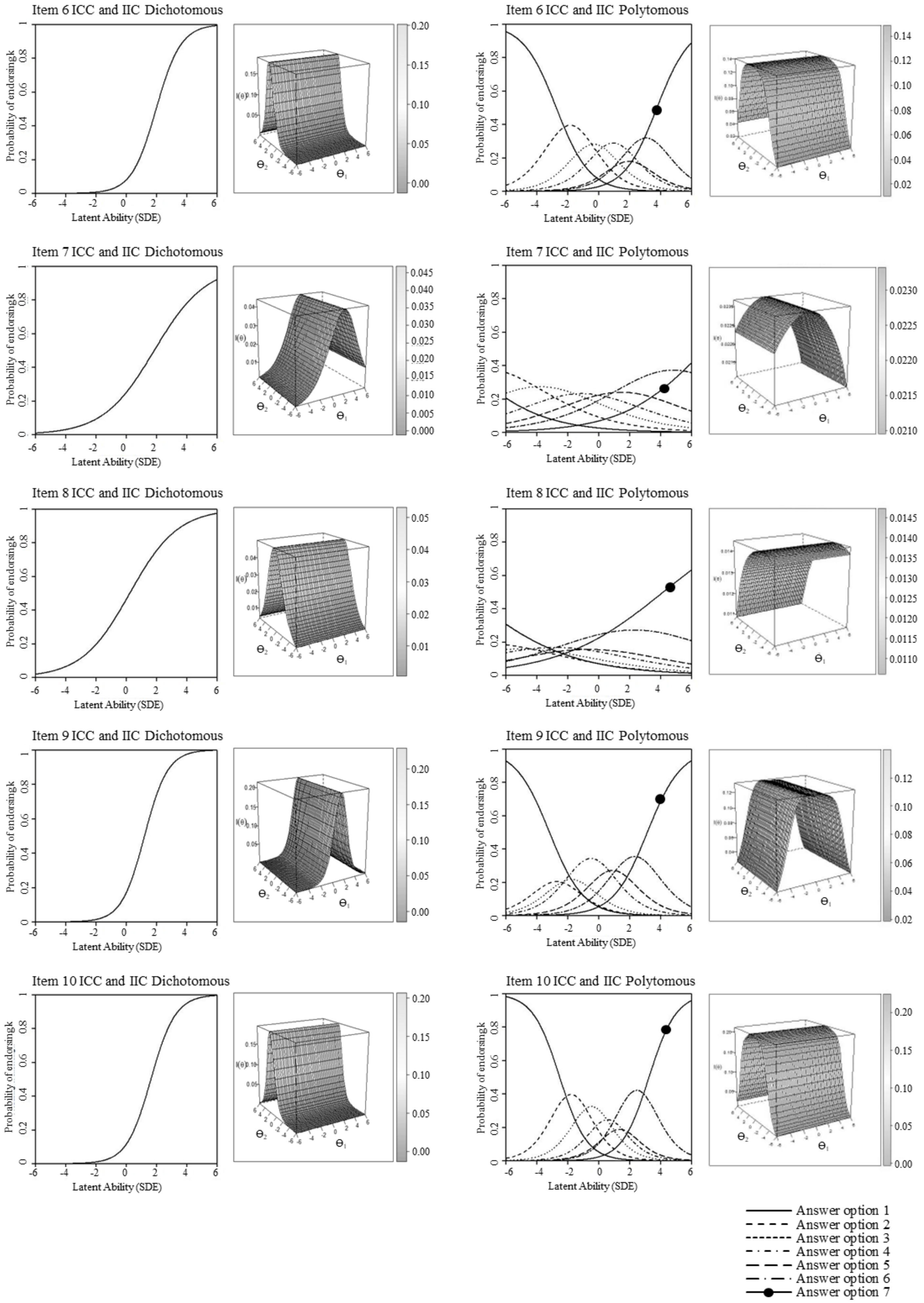

Figure S1 Continued

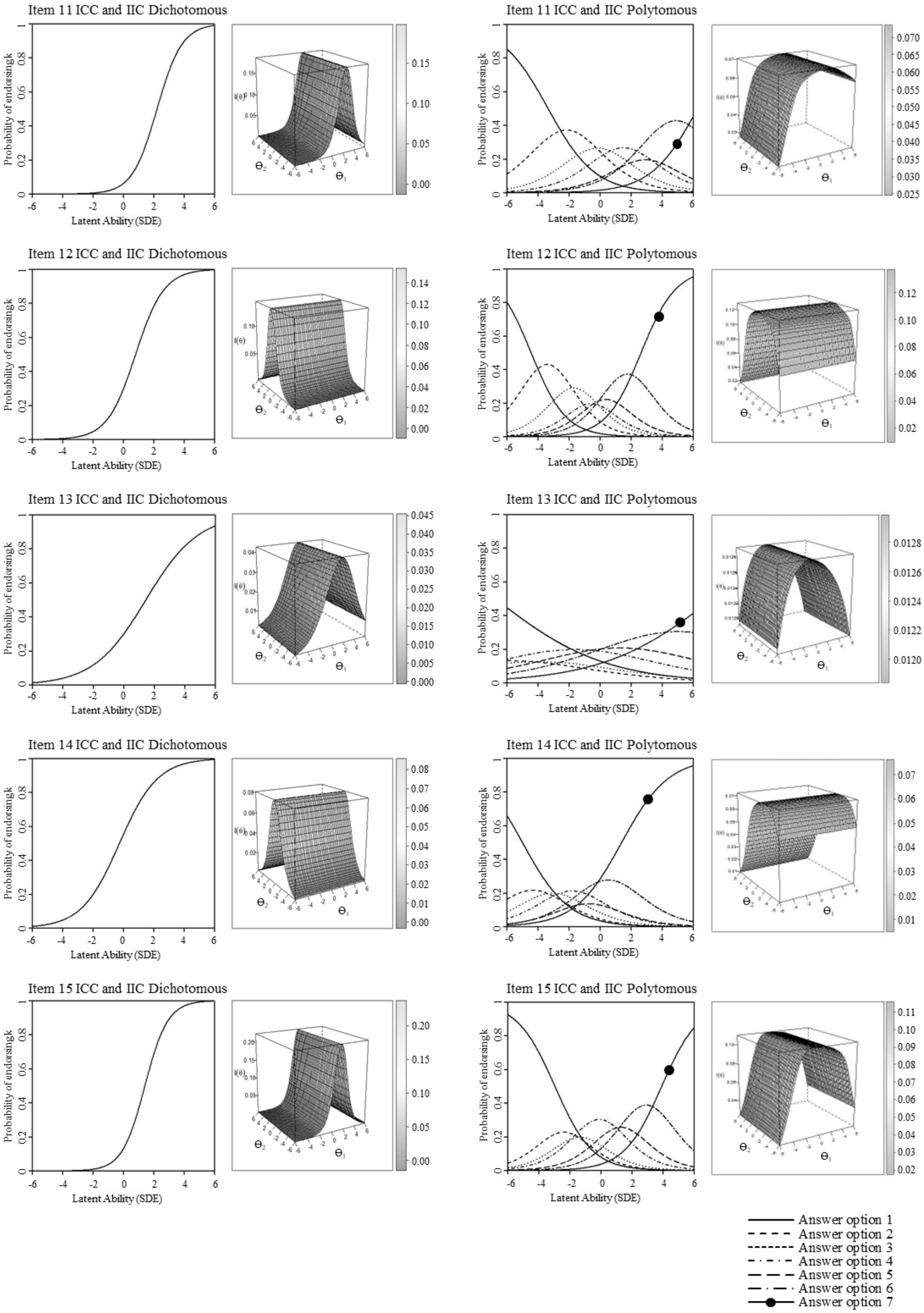

Figure S1 Continued

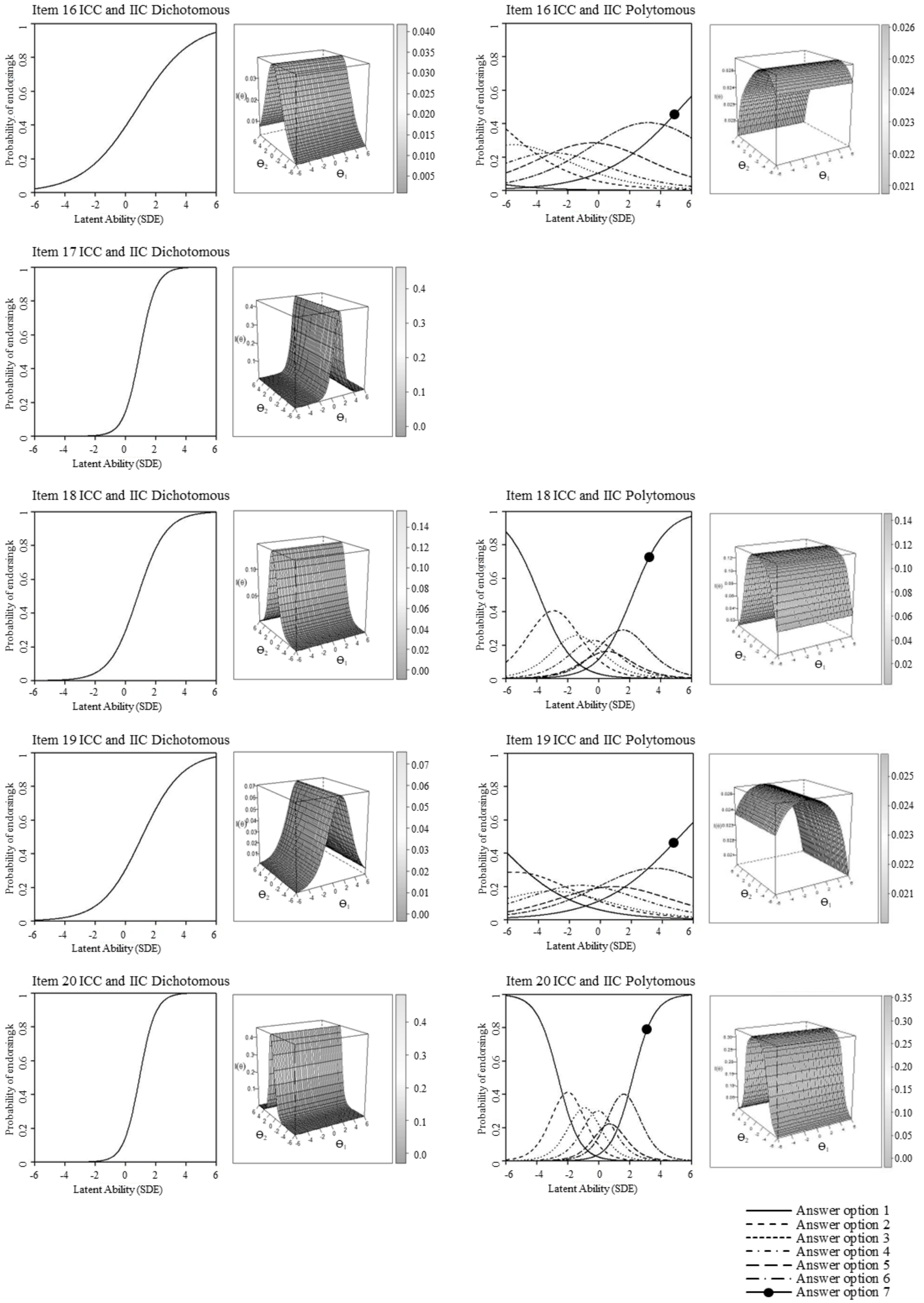

Figure S2

Item Characteristic Curve (ICC) and Item Information Curve (IIC) for IM Items Dichotomous (Left Rows) and Polytomous Scoring (Right Rows) of the BIDR 40-item Original Version

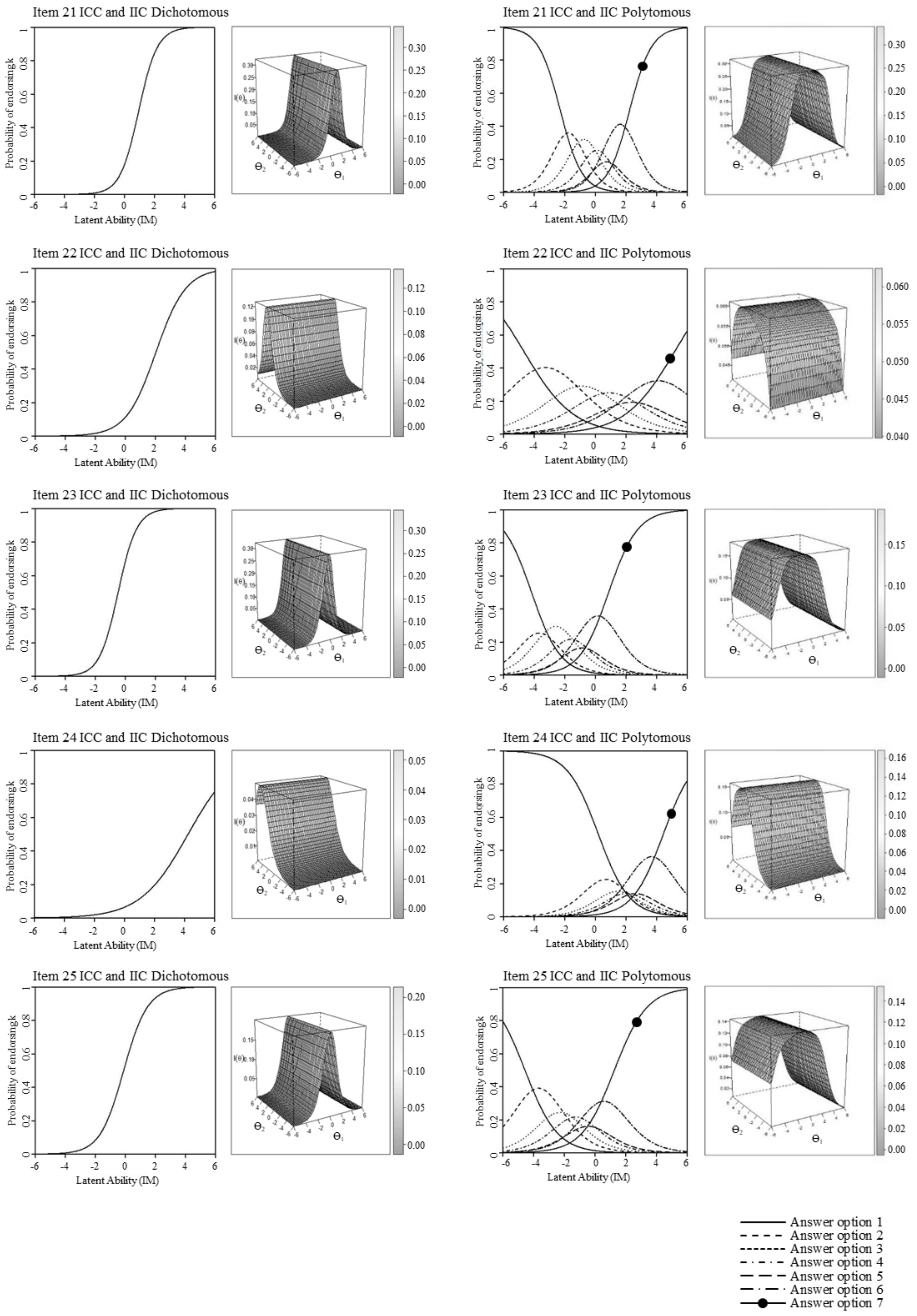

Figure S2 Continued

Item 26 ICC and IIC Dichotomous

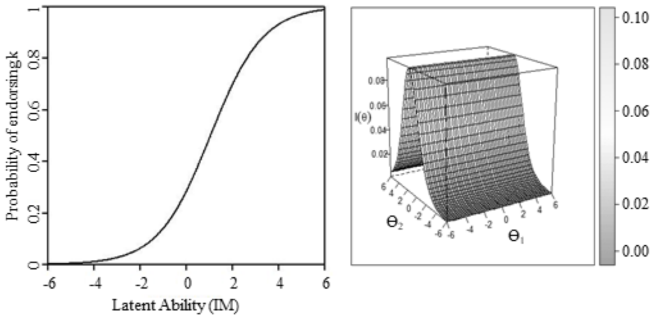

Item 26 ICC and IIC Polytomous

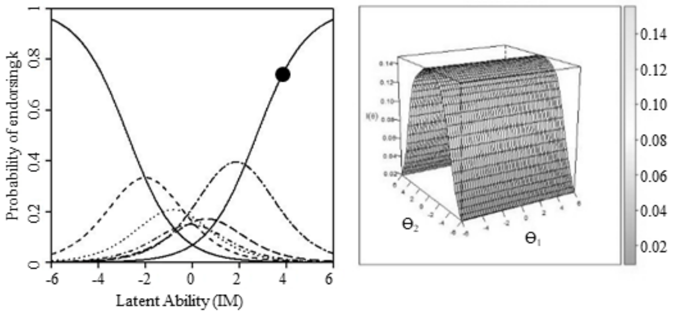

Item 27 ICC and IIC Dichotomous

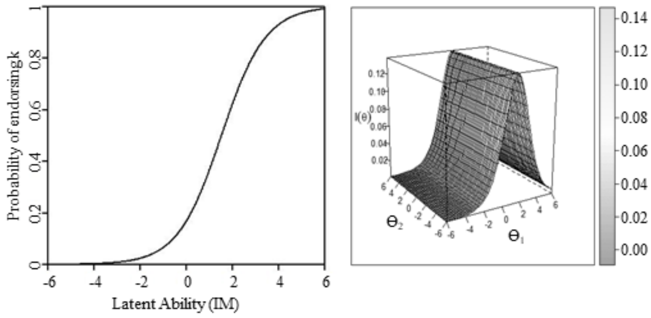

Item 27 ICC and IIC Polytomous

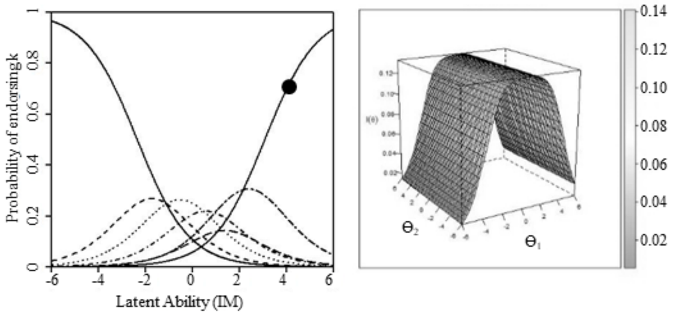

Item 28 ICC and IIC Dichotomous

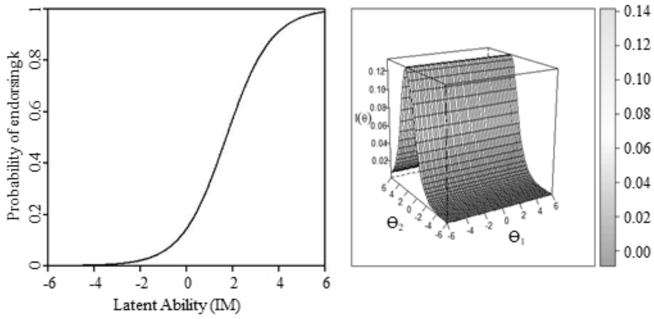

Item 28 ICC and IIC Polytomous

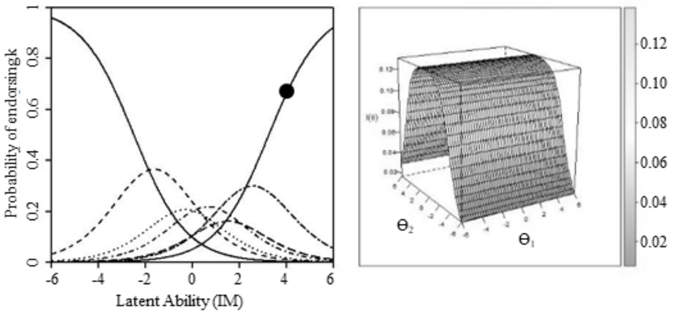

Item 29 ICC and IIC Dichotomous

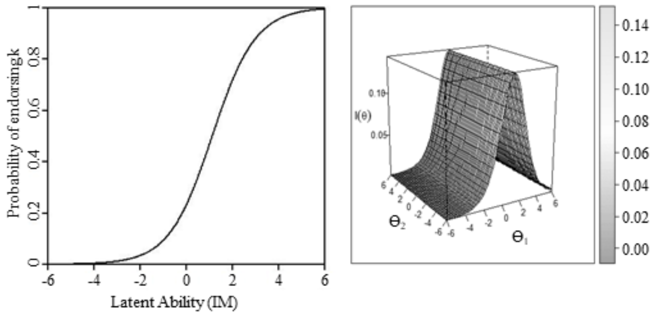

Item 29 ICC and IIC Polytomous

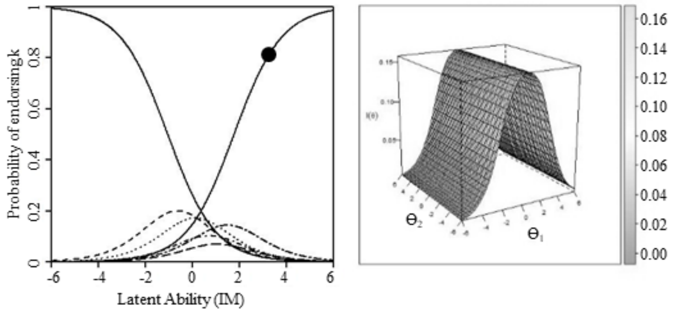

Item 30 ICC and IIC Dichotomous

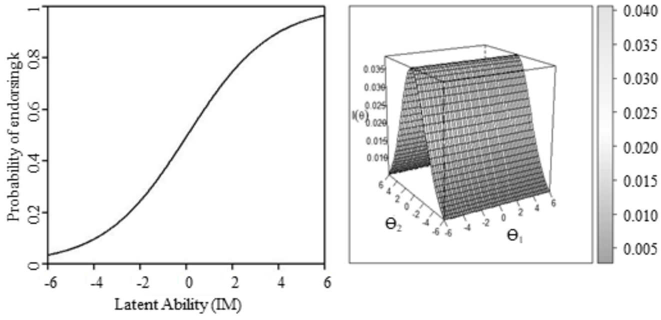

Item 30 ICC and IIC Polytomous

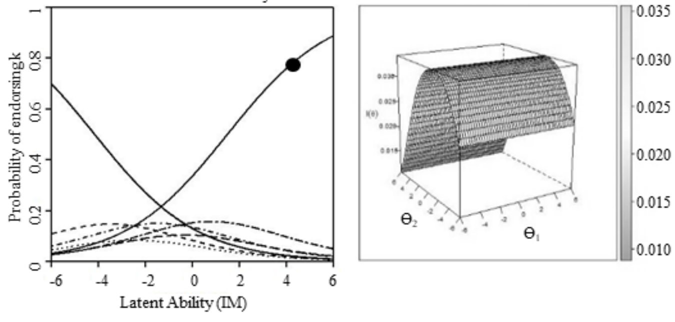

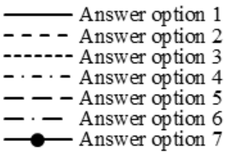

Figure S2 Continued

Item 31 ICC and IIC Dichotomous

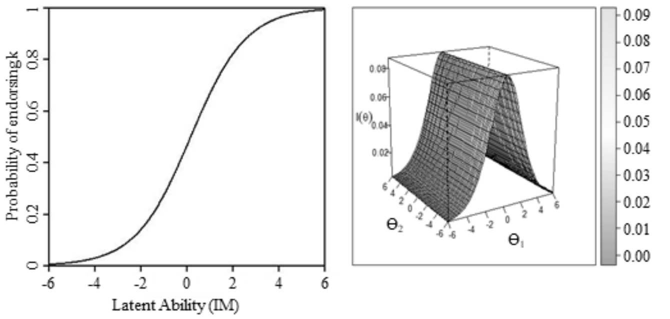

Item 31 ICC and IIC Polytomous

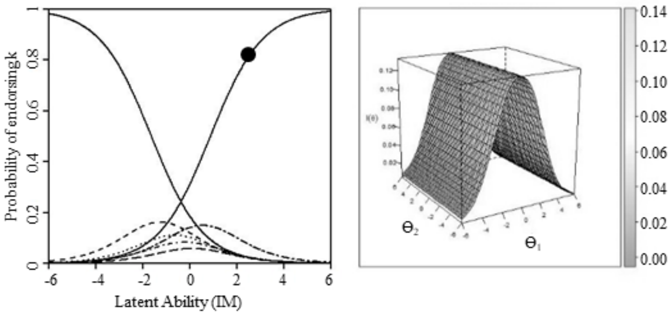

Item 32 ICC and IIC Dichotomous

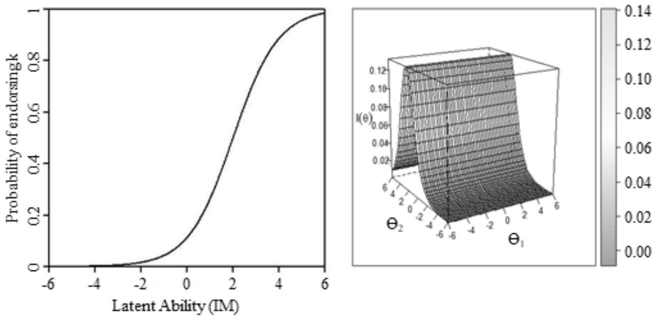

Item 32 ICC and IIC Polytomous

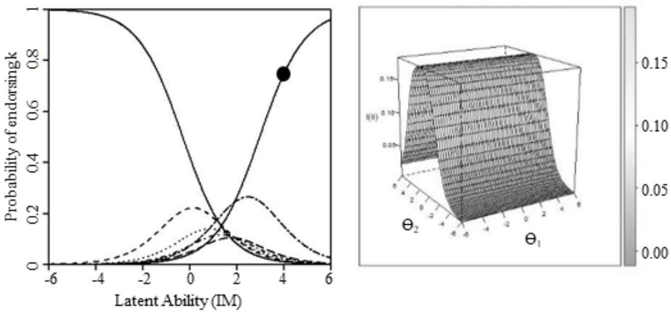

Item 33 ICC and IIC Dichotomous

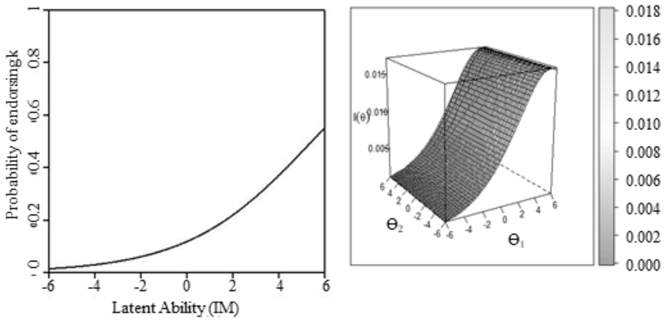

Item 33 ICC and IIC Polytomous

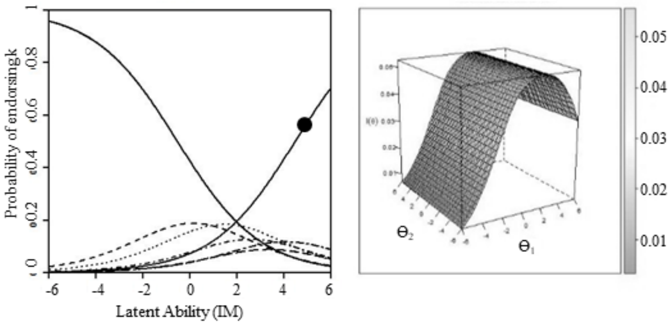

Item 34 ICC and IIC Dichotomous

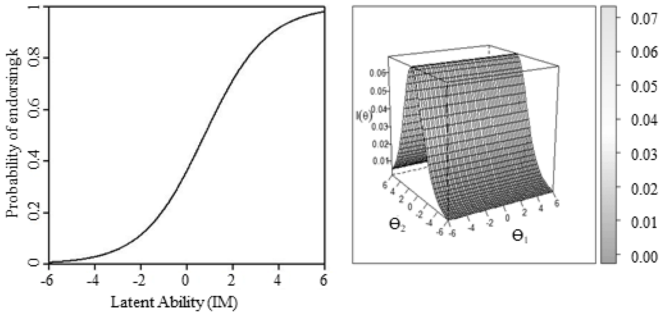

Item 34 ICC and IIC Polytomous

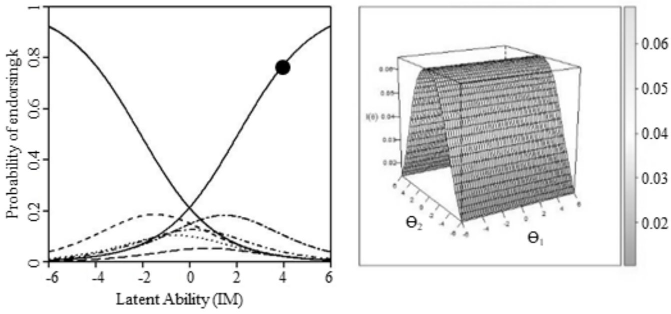

Item 35 ICC and IIC Dichotomous

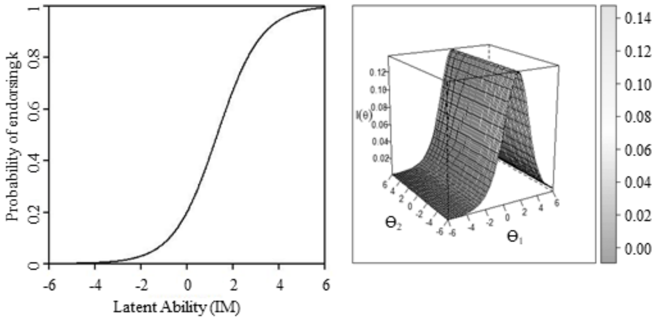

Item 35 ICC and IIC Polytomous

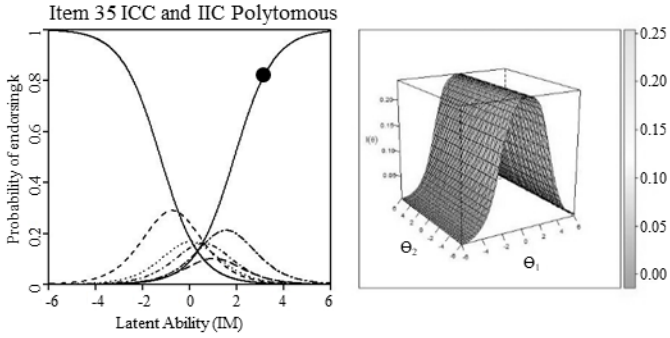

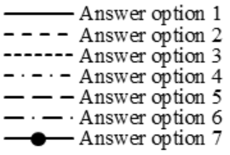

Figure S2 Continued

Item 36 ICC and IIC Dichotomous

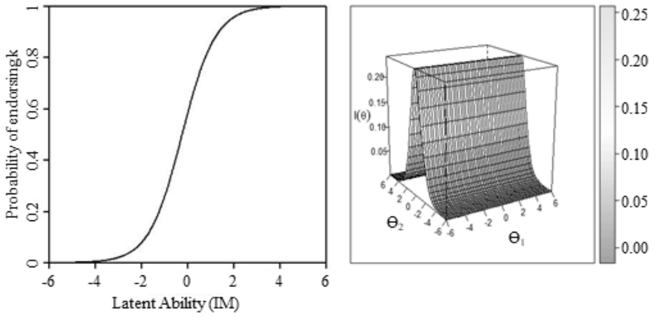

Item 36 ICC and IIC Polytomous

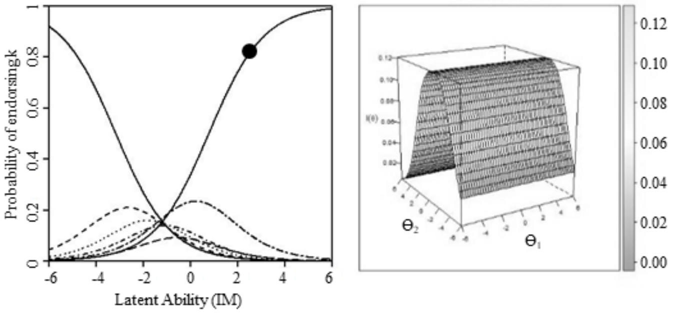

Item 37 ICC and IIC Dichotomous

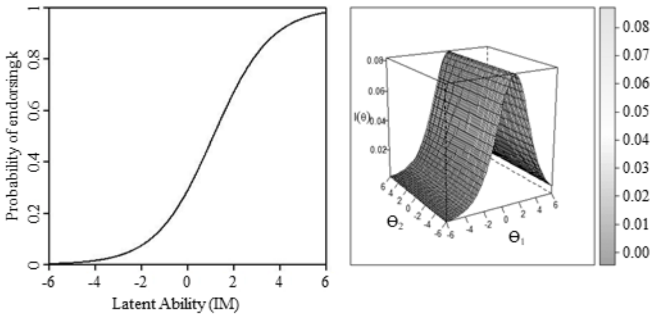

Item 37 ICC and IIC Polytomous

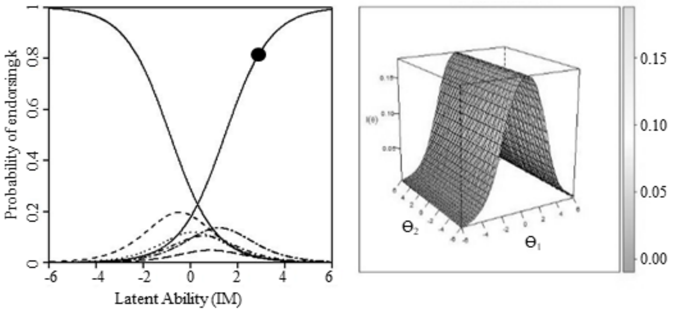

Item 38 ICC and IIC Dichotomous

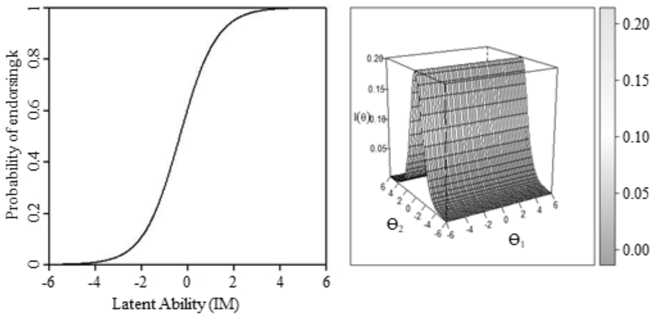

Item 38 ICC and IIC Polytomous

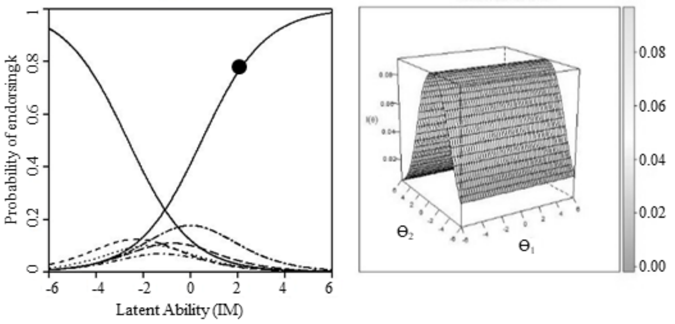

Item 39 ICC and IIC Dichotomous

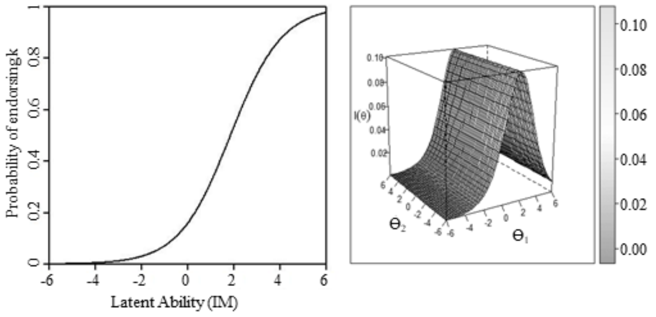

Item 39 ICC and IIC Polytomous

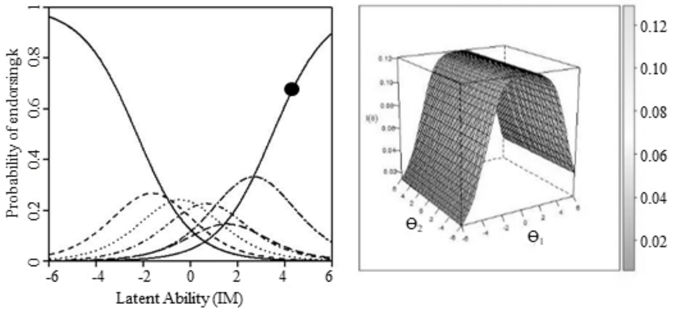

Item 40 ICC and IIC Dichotomous

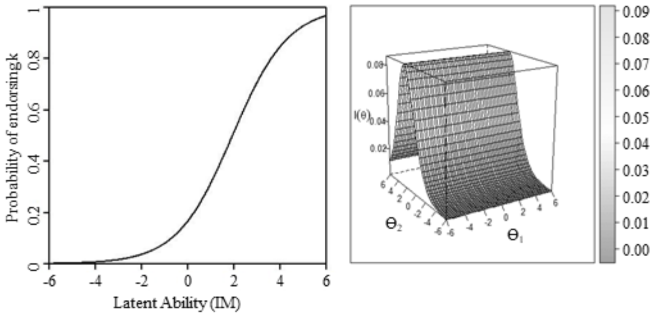

Item 40 ICC and IIC Polytomous

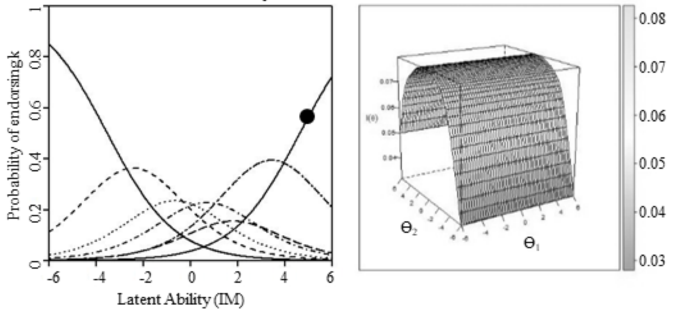

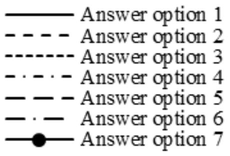

2.2. Omega Coefficient, Test-Retest Correlations and Correlations between BIDR forms Study 1. Mean and standard deviations of the Original BIDR (Version 6) and short forms Studies 1-3.

This file is supplementary to the main text, and it contains the exact statistics regarding psychometric properties of the original BIDR (Version 6) and the short forms BIDR-DP12 and BIDR-D20. In addition it provides the reader with complementary information regarding the mean and standard deviations of the original BIDR (Version 6), and the BIDR-DP12 and BIDR-D20 of Studies 1-3.

Table S7

Omega Coefficient, Test-Retest Correlations and Correlations Between SDE and IM Original Version and Short Forms Time 1 and 2

	Item	Measure	ω [95%CI] time 1	ω [95%CI] time 2	1	1.1	1.2	2	2.1	2.2	3	4	5
SDE	1	BIDR original Dichotomous	.84 [.82, .86]	.86 [.82, .91]	**.79**	.85	.82	.86	.67	.67	.76	.65	.86
	1.1	Denial	.79 [.77, .82]	.80 [.73, .86]	.84	**.70**	.42	.77	.86	.36	.84	.76	.76
	1.2	Enhancement	.78 [.75, .81]	.83 [.78, .89]	.80	.38	**.71**	.68	.27	.81	.43	.34	.68
	2	BIDR original Polytomous	.68 [.65, .72]	.77 [.70, .84]	.81	.70	.62	**.85**	.79	.78	.70	.78	.77
	2.1	Denial	.66 [.62, .70]	.73 [.65, .82]	.64	.82	.22	.79	**.77**	.27	.74	.90	.62
	2.2	Enhancement	.64 [.59, .68]	.68 [.58, .78]	.61	.25	.79	.74	.22	**.80**	.36	.35	.58
	3	BIDR-**D**P12	.78 [.75, .81]	.76 [.68, .83]	.81	.83	.49	.71	.72	.36	**.68**	.83	.87
	4	BIDR-D**P**12	.66 [.62, .71]	.69 [.59, .79]	.62	.70	.30	.79	.89	.32	.80	**.77**	.72
	5	BIDR-D20	.81 [.79, .84]	.81 [.74, .87]	.88	.71	.73	.76	.59	.60	.86	.68	**.80**
	5.1	Denial	.73 [.70, .77]	.72 [.63, .81]	.75	.81	.40	.67	.71	.31	.90	.77	.82
	5.2	Enhancement	.77 [.74, .80]	.74 [.65, .82]	.72	.39	.84	.61	.28	.70	.54	.38	.83
IM	6	BIDR original Dichotomous	.84 [.82, .86]	.86 [.81, .90]	.35	.42	.17	.28	.37	.05	.38	.37	.32
	6.1	Denial	.78 [.75, .80]	.76 [.68, .83]	.32	.42	.11	.28	.42	.00	.37	.40	.29
	6.2	Enhancement	.72 [.69, .76]	.82 [.76, .87]	.29	.29	.21	.20	.20	.10	.27	.20	.25
	7	BIDR original Polytomous	.77 [.74, .79]	.76 [.69, .84]	.18	.29	.01	.24	.35	.00	.27	.37	.19
	7.1	Denial	.70 [.66, .74]	.66 [.55, .77]	.18	.31	-.03	.24	.39	-.05	.28	.41	.19
	7.2	Enhancement	.60 [.55, .65]	.68 [.58, .78]	.15	.18	.07	.18	.19	.07	.18	.22	.14
	8	BIDR-**D**P12	.72 [.69, .76]	.83 [.78, .89]	.33	.40	.16	.28	.38	.04	.37	.37	.30
	9	BIDR-D**P**12	.61 [.56, .66]	.68 [.57, .79]	.25	.34	.06	.28	.39	.03	.33	.40	.24
	10	BIDR-D20	.80 [.77, .82]	.80 [.73, .86]	.34	.41	.17	.26	.35	.04	.37	.34	.30
	10.1	Denial	.75 [.71, .78]	.74 [.65, .82]	.29	.40	.09	.24	.38	-.02	.35	.36	.26
	10.2	Enhancement	.65 [.61, .70]	.62 [.51, .73]	.29	.28	.22	.18	.19	.11	.26	.18	.24

Note. Test-retest correlations are diagonal and bold. Under the diagonal are correlations at time 1 (N = 577), above the diagonal are correlations at time 2 (N = 87). SDE = Self-Deceptive Enhancement, IM = Impression Management, BIDR-DP12 = short version of the BIDR that can be used Dichotomous or Polytomous, BIDR-D20 = 20 item short version that can only be used Dichotomous.

Table S7 Continued

	Item	Measure	5.1	5.2	6	6.1	6.2	7	7.1	7.2	8	9	10	10.1	10.2
SDE	1	BIDR original Dichotomous	.70	.75	.39	.37	.32	.30	.25	.28	.37	.33	.39	.45	.19
	1.1	Denial	.83	.46	.39	.41	.28	.31	.32	.22	.37	.28	.41	.50	.15
	1.2	Enhancement	.35	.83	.25	.17	.25	.18	.08	.23	.22	.25	.23	.22	.17
	2	BIDR original Polytomous	.66	.65	.30	.31	.23	.33	.28	.30	.27	.33	.34	.39	.14
	2.1	Denial	.72	.32	.39	.46	.22	.41	.45	.25	.35	.35	.40	.51	.10
	2.2	Enhancement	.30	.72	.09	.04	.11	.12	.02	.19	.08	.18	.14	.12	.10
	3	BIDR-**D**P12	.91	.55	.39	.41	.28	.33	.34	.24	.41	.39	.42	.46	.21
	4	BIDR-D**P**12	.77	.44	.42	.45	.27	.48	.48	.32	.37	.43	.43	.48	.17
	5	BIDR-D20	.87	.82	.42	.37	.35	.36	.29	.33	.36	.35	.40	.42	.23
	5.1	Denial	**.65**	.45	.40	.39	.31	.35	.33	.28	.38	.34	.41	.43	.22
	5.2	Enhancement	.39	**.73**	.29	.20	.28	.24	.13	.28	.20	.24	.26	.25	.16
IM	6	BIDR original Dichotomous	.35	.19	**.75**	.85	.87	.87	.72	.72	.78	.73	.88	.78	.68
	6.1	Denial	.36	.13	.86	**.78**	.50	.76	.86	.41	.79	.74	.80	.89	.38
	6.2	Enhancement	.22	.21	.81	.43	**.61**	.73	.40	.84	.58	.53	.72	.47	.78
	7	BIDR original Polytomous	.25	.05	.85	.76	.66	**.81**	.84	.81	.63	.74	.75	.65	.57
	7.1	Denial	.28	.03	.74	.86	.36	.88	**.80**	.40	.60	.72	.63	.70	.28
	7.2	Enhancement	.15	.08	.72	.41	.84	.82	.47	**.70**	.45	.51	.62	.38	.69
	8	BIDR-**D**P12	.33	.16	.77	.82	.46	.62	.65	.40	**.76**	.88	.86	.84	.57
	9	BIDR-D**P**12	.31	.09	.70	.74	.43	.73	.74	.49	.87	**.76**	.79	.74	.54
	10	BIDR-D20	.32	.19	.90	.79	.72	.74	.65	.62	.81	.71	**.73**	.87	.78
	10.1	Denial	.31	.12	.77	.88	.40	.65	.73	.36	.85	.73	.86	**.72**	.41
	10.2	Enhancement	.21	.21	.72	.40	.87	.58	.33	.73	.47	.42	.79	.39	**.65**

Note. Test-retest correlations are diagonal and bold. Under the diagonal are correlations at time 1 (N = 577), above the diagonal are correlations at time 2 (N = 87). SDE = Self-Deceptive Enhancement, IM = Impression Management, BIDR-DP12 = short version of the BIDR that can be used Dichotomous or Polytomous, BIDR-D20 = 20 item short version that can only be used Dichotomous.

Table S8
Mean and Standard Deviations of the BIDR-DP12 and BIDR-D20

			Study 1		Study 2	Study 3
			Time 1	Time 2		
		Range	***M (SD)***		***M (SD)***	***M (SD)***
**SDE**	BIDR original Dichotomous	0-40	5.74 (3.47)	5.95 (3.76)	5.40 (3.35)	7.41 (3.51)
	Denial	0-20	3.14 (2.18)	3.35(2.28)	2.74 (1.95)	3.60 (2.24)
	Enhancement	0-20	2.57 (2.01)	2.61 (2.16)	2.66 (2.11)	3.81 (2.02)
	BIDR original Polytomous	7-140	86.14 (11.98)	87.05 (13.08)	85.33 (11.56)	89.78 (12.25)
	Denial	7-70	43.38 (8.11)	44.60 (8.43)	41.65 (7.81)	43.29 (8.15)
	Enhancement	7-70	42.65 (7.39)	42.70 (7.99)	43.68 (7.61)	46.49 (6.68)
	BIDR-**D**P12	0-7	1.81 (1.69)	1.87 (1.66)	1.53 (1.46)	2.25 (1.78)
	BIDR-D**P**12	7-49	28.83 (6.47)	29.63 (6.30)	27.70 (6.14)	29.28 (7.16)
	BIDR-D20	0-20	2.38 (2.10)	2.44 (2.12)	2.14 (1.95)	3.49 (2.27)
	Denial	0-10	1.15 (1.24)	1.26 (1.28)	0.93 (1.11)	1.51 (1.43)
	Enhancement	0-10	1.22 (1.28)	1.17 (1.23)	1.21 (1.28)	1.98 (1.35)
**IM**	BIDR original Dichotomous	0-40	6.32 (3.49)	6.21 (3.67)	6.75 (4.01)	5.98 (3.59)
	Denial	0-20	3.24 (2.14)	3.06 (2.12)	3.41 (2.34)	3.32 (2.08)
	Enhancement	0-20	3.09 (1.93)	3.11 (2.14)	3.34 (2.22)	2.66 (1.97)
	BIDR original Polytomous	7-140	77.83 (16.90)	78.65 (16.45)	82.97 (16.87)	74.65 (16.25)
	Denial	7-70	39.24 (10.40)	39.25 (9.66)	41.36 (10.37)	39.63 (9.52)
	Enhancement	7-70	38.62 (9.06)	39.10 (9.56)	41.61 (9.42)	35.02 (8.77)
	BIDR-**D**P12	0-5	1.81 (1.33)	1.69 (1.48)	1.83 (1.44)	2.20 (1.45)
	BIDR-D**P**12	5-35	21.85 (5.33)	21.62 (5.62)	22.15 (5.48)	23.03 (5.50)
	BIDR-D20	0-20	3.56 (2.18)	3.48 (2.42)	3.68 (2.37)	4.04 (2.41)
	Denial	0-10	1.93 (1.41)	1.99 (1.68)	1.99 (1.47)	2.12 (1.36)
	Enhancement	0-10	1.63 (1.20)	1.48 (1.19)	1.69 (1.47)	1.92 (1.36)

Note . *N* study 1 time 1 = 577, *N* study 1 time 2 = 87, *N* study 2 = 719, *N* study 3 = 100. SDE = Self-Deceptive Enhancement, IM = Impression Management, BIDR-DP12 = short version of the BIDR that can be used Dichotomous or Polytomous, BIDR-D20 = 20 item short version that can only be used Dichotomous.

2.3. IRT results of the BIDR-D20 Study 2.

This file contains the exact output for the IRT analyses (i.e. model fit, local dependence and item fit, estimator parameters, ICCs and IICs) of Study 2.

All files are meant to be helpful for properly interpreting the output, understand and evaluate the conclusions made in the full paper as well as making our results reproducible.

Table S9

Fit Statistics for the IRT models for SDE and IM Dichotomous Only Scoring

	Dichotomous 2PL			
	SDE		IM	
Model	one factor	two factors	one factor	two factors
-2LL	-3420.396	**-3405.840**	-3929.912	**-3914.701**
M2 (df)	116.222 (35)*	81.988 (34)*	113.625 (35)*	80.821 (34)*
RMSEA2 (CI.90%)	.057 (.046, .068)	**.044 (.032, .057)**	.056 (.045, .068)	**.044 (.032, .056)**
SRMSR	.057	**.047**	.048	**.043**
AIC	6880.79	**6853.68**	7899.82	**7871.40**
BIC	6972.35	**6949.82**	7991.38	**7967.54**
saBIC	6908.84	**6883.13**	7927.88	**7900.27**
Estimated parameters	20	21	20	21
$-2LL_{change}$	14.556 (1), $p < .001$		15.211 (1), $p < .001$	

Note. -2LL = log-likelihood test, RMSEA2 = bivariate Root Mean Square Error of Approximation, SRMSR = Standardized Root Mean Square Residual, AIC = Akaike Information Criterion, BIC = Bayesian Information Criterion, and saBIC = sample size adjusted BIC. SDE = Self-Deceptive Enhancement, IM = Impression Management. The one factor scale represents the unidimensional construct SDE or IM, Two factors indicate items of SDE and IM divided in Denial and Enhancement based on reversed and non-reversed items. *Statistically significant model misfit at $p < .001$. **Bold** indicates when model fit index indicates the better fitting model.

Table S10

Local Independence Matrix for Dichotomous Scoring and Item Fit Statistics for SDE

Item nr.	Items	3	5	9	15	17	4	6	10	18	20	S-X2 (df, *p*) Dichotomous
3	I don't care to know what other people really think of me.		-.17	-.12	-.14	-.30	.00	-.02	.09	.10	-.11	9.937 (5,.540)
5	I always know why I like things.			-.26	-.34	-.38	.02	-.08	-.02	-.07	.11	4.284 (4, .643)
9	I am fully in control of my own fate.				-.28	-.32	.08	-.06	-.02	.06	-.04	7.565 (4, .540)
15	I am a completely rational person.					-.13	-.01	.09	.01	-.08	-.01	4.636 (5, .646)
17	I am very confident of my judgments						-.08	.08	-.02	.02	.00	3.252 (4, .646)
4	I have not always been honest with myself.(r)							-.17	-.21	-.20	-.37	1.367 (5, .928)
6	When my emotions are aroused, it biases my thinking.(r)								-.22	-.14	-.38	5.630 (5, .643)
10	It's hard for me to shut off a disturbing thought.(r)									-.11	-.32	1.993 (5, .928)
18	I have sometimes doubted my ability as a lover.(r)										-.29	5.255 (5, .643)
20	I don't always know the reasons why I do the things I do.(r)											6.540 (4, .540)

Note. As an indication of local dependence, a cut-off of .30 minus the average correlation was used as a critical value for *Q* 3 (for more information see Christensen et al., 2017). *p* -values were adjusted for false discovery rates (FDR; Benjamini & Hochberg, 1995). (r) indicates reversed coding.

Table S11

Local Independence Matrix for Dichotomous Scoring and Item Fit Statistics for IM

Item nr.	Items	21	23	25	29	35	22	28	32	36	38	S-X2 (df, *p*) Dichotomous
21	I sometimes tell lies if I have to.(r)		-.37	-.34	-.22	-.24	.10	.04	-.06	.02	-.09	5.622 (5, .575)
23	There have been occasions when I have taken advantage of someone.(r)			-.35	-.26	-.23	-.03	-.05	.00	.01	.06	12.411 (4, .150)
25	I sometimes try to get even rather than forgive and forget.(r)				-.21	-.10	-.05	-.05	.06	-.04	.07	3.172 (5, .686)
29	I have received too much change [..] without telling him or her.(r)					-.08	-.05	.09	-.03	.06	-.07	3.928 (6, .686)
35	I have done things that I don't tell other people about.(r)						.01	.00	.03	-.05	.02	10.402 (6, .545)
22	I never cover up my mistakes.							-.12	-.23	-.25	-.26	8.225 (6, .575)
28	When I hear people talking privately, I avoid listening.								-.25	-.32	-.23	7.461 (6, .575)
32	I have never dropped litter on the street.									-.17	-.18	4.625 (6, .686)
36	I never take things that don't belong to me.										-.44	4.651 (4, .575)
38	I have never damaged a library book [..] without reporting it.											3.990 (5, .686)

Note. As an indication of local dependence, a cut-off of .30 minus the average correlation was used as a critical value for $Q3$ (for more information see Christensen et al., 2017). *p*-values were adjusted for false discovery rates (FDR; Benjamini & Hochberg, 1995).

Table S12

Factor Loadings, Discrimination, Threshold, and Difficulty Parameters for the BIDR-D20

Item No.	Items	Factor Loading		*a1*	97.5% CI *a1*		*a2*	97.5% CI *a2*		*d*	97.5% CI *d*		*b*
		1	2		*2.5*	*97.5*		*2.5*	*97.5*		*2.5*	*97.5*	
SDE													
3	I don't care to know what other people really think of me.	.53		1.07	0.71	1.42				-1.99	-2.29	-1.69	1.86
5	I always know why I like things.	.63		1.39	0.99	1.79				-0.79	-1.01	-0.56	0.57
9	I am fully in control of my own fate.	.59		1.25	0.88	1.61				-1.36	-1.62	-1.10	1.09
15	I am a completely rational person.	.60		1.27	0.87	1.67				-1.85	-2.17	-1.54	1.46
17	I am very confident of my judgments	.69		1.63	1.13	2.12				-1.69	-2.04	-1.34	1.04
4	I have not always been honest with myself.		.56				1.15	0.77	1.53	-1.76	-2.06	-1.47	1.54
6	When my emotions are aroused, it biases my thinking.		.65				1.45	0.93	1.97	-2.83	-3.34	-2.32	1.95
10	It's hard for me to shut off a disturbing thought.		.59				1.23	0.80	1.66	-2.36	-2.75	-1.98	1.92
18	I have sometimes doubted my ability as a lover.		.43				0.81	0.52	1.10	-1.16	-1.36	-0.95	1.43
20	I don't always know the reasons why I do the things I do.		.69				1.62	1.09	2.14	-1.72	-2.08	-1.35	1.06
IM													
21	I sometimes tell lies if I have to.		.77				2.02	1.48	2.57	-1.20	-1.51	-0.88	0.59
23	There have been occasions when I have taken advantage of someone.		.76				1.97	1.46	2.48	0.68	0.42	0.94	0.46
25	I sometimes try to get even rather than forgive and forget.		.62				1.34	1.01	1.67	0.02	-0.18	0.21	-0.11
29	I have received too much change [..] without telling him or her.		.51				1.01	0.74	1.29	-0.94	-1.14	-0.74	1.10
35	I have done things that I don't tell other people about.		.49				0.96	0.68	1.24	-1.32	-1.54	-1.10	-1.29
22	I never cover up my mistakes.	.60		1.27	0.89	1.64				-2.01	-2.33	-1.69	1.58
28	When I hear people talking privately, I avoid listening.	.61		1.32	0.94	1.70				-1.85	-2.16	-1.54	1.40
32	I have never dropped litter on the street.	.57		1.18	0.83	1.53				-1.99	-2.30	-1.69	1.69
36	I never take things that don't belong to me.	.71		1.73	1.25	2.21				0.44	0.21	0.68	-0.26
38	I have never damaged a library book [..] without reporting it.	.65		1.44	1.06	1.82				0.43	0.22	0.64	-0.30

Note. *a* is the discrimination parameter (or slope). The mirt package in R only provides estimates for intercepts which can be transformed into threshold values for each item using the following formula ($-d/a$), where *d* is the intercept value for the corresponding response category and *a* is the slope for the item. Factor 1 = Enhancement, Factor 2 = Denial. Darker notation indicate greater factor loadings (< .40; .40 - .55; ≥ .55) and discriminantion parameters (< .65; .65 - 1.34; >1.34).

Figure S3

Item Characteristic Curve (ICC) and Item Information Curve (IIC) for SDE Items Dichotomous Scoring of the BIDR-D20

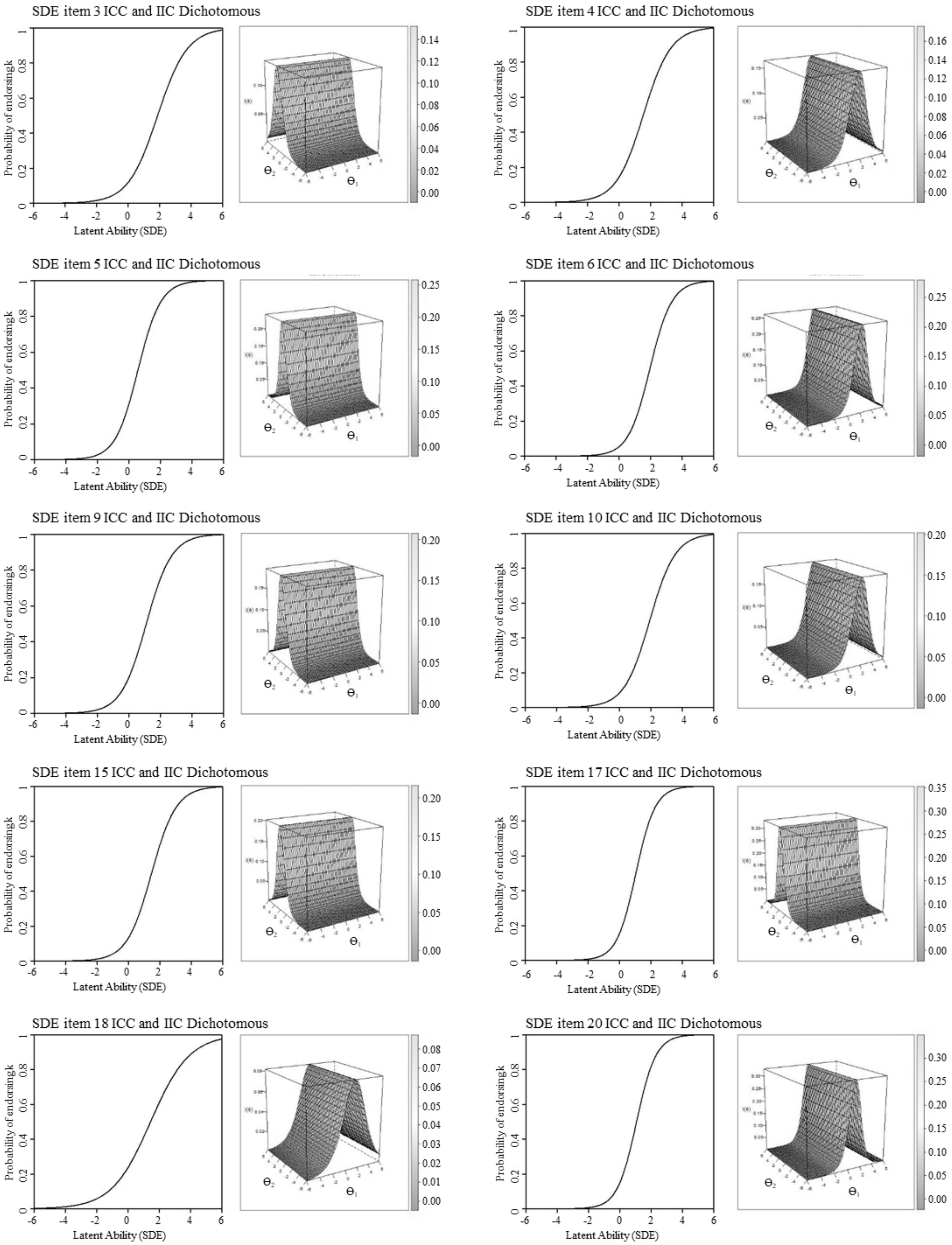

Figure S4

Item Characteristic Curve (ICC) and Item Information Curve (IIC) for IM Items Dichotomous Scoring of the BIDR-D20

IM item 21 ICC and IIC Dichotomous

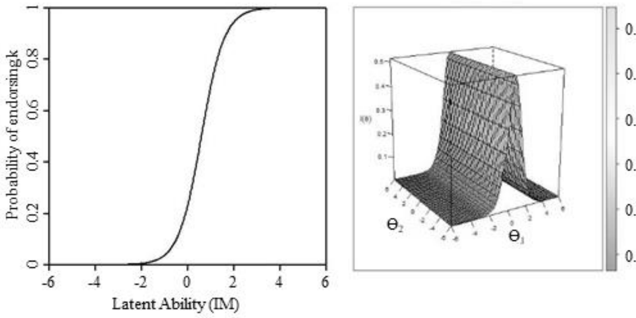

IM item 22 ICC and IIC Dichotomous

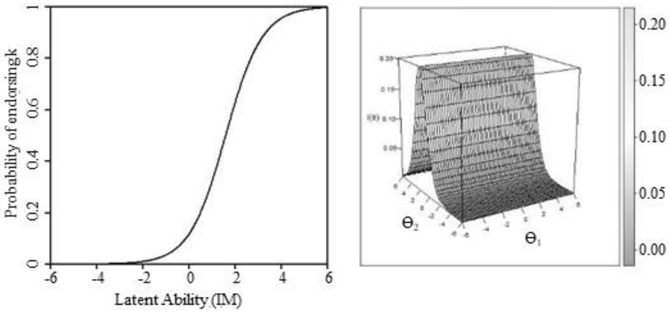

IM item 23 ICC and IIC Dichotomous

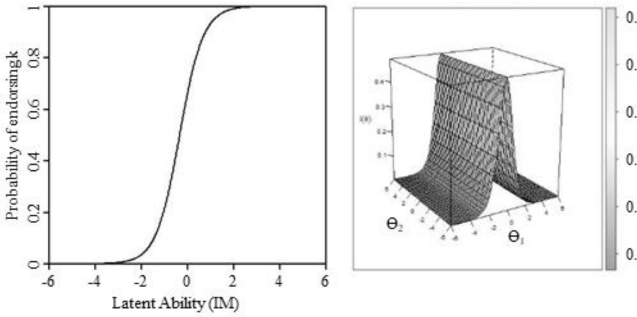

IM item 25 ICC and IIC Dichotomous

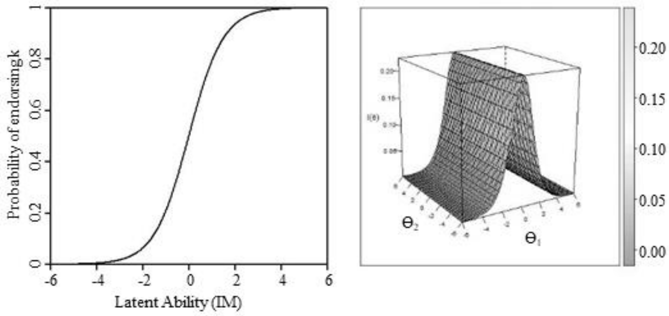

IM item 28 ICC and IIC Dichotomous

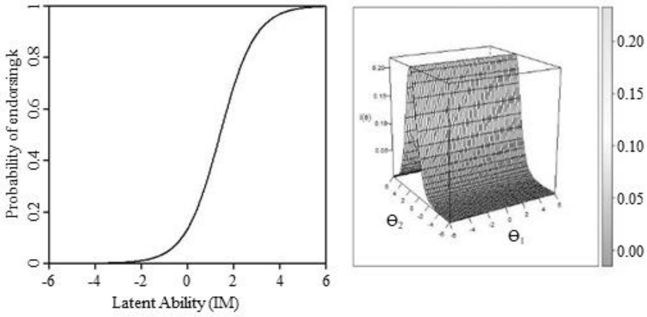

IM item 29 ICC and IIC Dichotomous

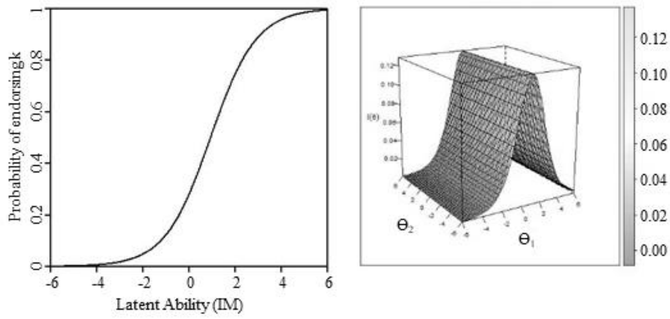

IM item 32 ICC and IIC Dichotomous

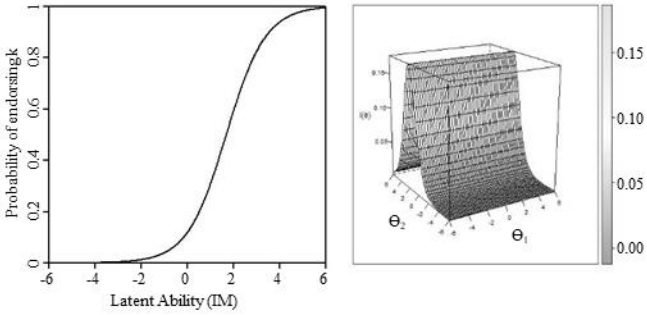

IM item 35 ICC and IIC Dichotomous

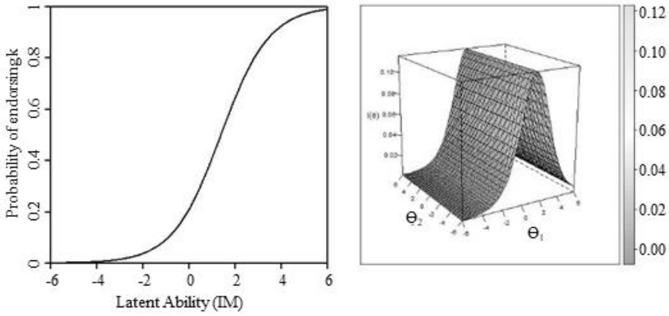

IM item 36 ICC and IIC Dichotomous

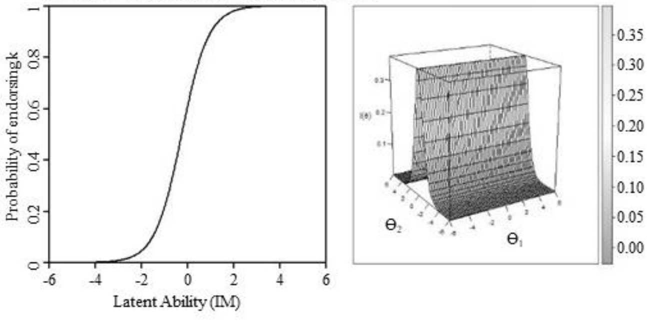

IM item 38 ICC and IIC Dichotomous

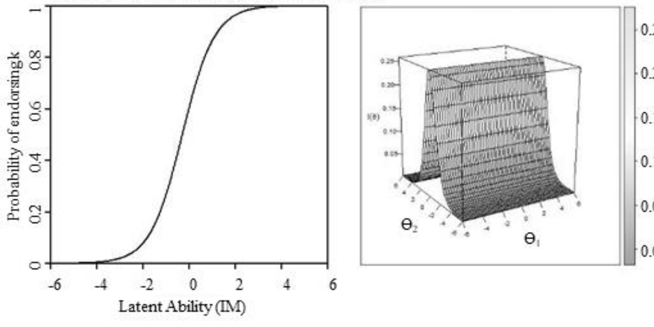

Supplement: Supplementary file 1 [file Supplementary_file_1.pdf]
